# Single-cell multimodal profiling of pan-cancer cell lines uncovers gene regulatory principles underlying intrinsic cell states and environmental features

**DOI:** 10.1038/s41467-026-75360-7

**Published:** 2026-07-23

**Authors:** Zihan Xu, Aileen Ugurbil, Joshua Kwan, Chloe Schaefer, Abdulraouf Abdulraouf, Ziyu Lu, Erting Tang, Wei Zhou, Junyue Cao

**Affiliations:** 1 Laboratory of Single Cell Genomics and Population Dynamics, The Rockefeller University, New York, NY USA; 2 The David Rockefeller Graduate Program in Bioscience, The Rockefeller University, New York, NY USA; 3 The Tri-Institutional M.D.-Ph. D. Program, New York, NY USA; 4 Pritzker School of Molecular Engineering, University of Chicago, Chicago, IL USA

**Keywords:** Cancer genomics, Epigenomics, Cancer epigenetics

## Abstract

Cancer arises from genetic and epigenetic alterations that reshape chromatin, transcriptional regulation, and malignant cell states. To chart cancer-intrinsic regulatory programs, we build a pan-cancer single-cell atlas of 60 cancer cell lines spanning 16 tissue origins and 20 cancer types, comprising 240,957 snRNA-seq and 223,347 snATAC-seq profiles. Integrative analyses reveal cell-state heterogeneity, core gene-regulatory networks, and a conserved EMT axis transcending tissue of origin; copy-number analysis identifies transcription factor amplification and hyperactivation as drivers of state reprogramming. Comparing cutaneous melanoma with acral melanoma, a rare subtype underrepresented in previous studies, uncovers a universal inflammation-suppressive program in acral and an inflamed landscape in cutaneous melanoma, with JAK-STAT activity as the central discriminator. Integrating data across models and patient cohorts links tumor-intrinsic regulation to microenvironmental composition and therapeutic response. By profiling rare alongside common subtypes, this atlas offers a resource for mapping pan-cancer and subtype-specific regulatory programs shaping cell-state plasticity.

## Abstract (web summary)

‘The characterization of cancer intrinsic regulatory landscape remains elusive. Here, the authors generate a pan-cancer single cell transcriptomic and epigenomic atlas and identify subtype-specific gene-regulatory programs that shape cancer cell-state plasticity.

## Introduction

Gene regulation shapes cellular behavior, state transitions, and lineage development. Across chromatin and RNA layers, transcription factors (TFs) bind enhancers and promoters to modulate chromatin state and tune downstream gene expression^1^. Reconstructing gene regulatory networks (GRNs), which capture the interconnected regulatory architecture through which TFs coordinate gene expression programs, can therefore provide mechanistic insight into the regulation of cellular phenotypes^2,3^. Cancer cell states are shaped by extensive epigenetic and transcriptional remodeling that drives their aberrant behaviors^4^, yet the core regulatory programs shared across diverse malignancies remain poorly defined. Large-scale cancer genomics studies have cataloged molecular features, such as DNA mutations and bulk expression profiles across cancer types^5,6^, but bulk profiling masks regulatory heterogeneity among malignant cells and between malignant and non-malignant cells within tumors. Although recent single-cell studies have begun to resolve cellular heterogeneity in cancer^7–9^, many have focused on individual cancer types, single modalities, such as gene expression, or relatively small sample cohorts, restricting the ability to reconstruct regulatory circuits across epigenetic and transcriptional layers.

Cancer cell lines provide a uniquely tractable system in which tumor-intrinsic programs can be isolated from microenvironmental influences, enabling precise dissection of core GRNs across heterogeneous cancer lineages. While recent single-cell atlases of cancer cell lines have revealed extensive molecular heterogeneity^10–12^, most studies rely on droplet-based platforms that are restricted to a single molecular modality or limited cell numbers per experiment, constraining the ability to map chromatin–gene regulatory relationships across diverse cancer states. The development of EasySci platforms^13–15^, which provide an integrated combinatorial indexing solution compatible with multimodal profiling and high-throughput sample multiplexing, has opened new opportunities for dissecting cancer gene-regulatory dynamics at scale.

In this work, we use EasySci to construct a pan-cancer single-cell atlas comprising 60 human cancer cell lines, representing 16 tissue origins and 20 cancer types, and more than 460,000 transcriptomic and chromatin accessibility profiles. Through integrative analysis of these multimodal data, we uncover both lineage-specific and pan-cancer epithelial–mesenchymal transition (EMT) axes. We further demonstrate how TF amplification alters cell state through regulatory hyperactivation, and identify a striking regulatory divergence between acral and cutaneous melanoma that is concordant with their distinct original microenvironmental features, therapeutic responses and patient outcomes. Together, these findings reveal shared cell states across cancer contexts and demonstrate that subtype-defining cancer-intrinsic regulatory logic captured in vitro can predict clinical outcomes, thereby highlighting the robustness of cancer cell-intrinsic regulatory programs observed in vitro and their relevance to global tumor phenotypes and clinical behaviors.

## Results

### Single-cell multi-omic atlas of diverse human cancer cell lines

**Fig. 1:**
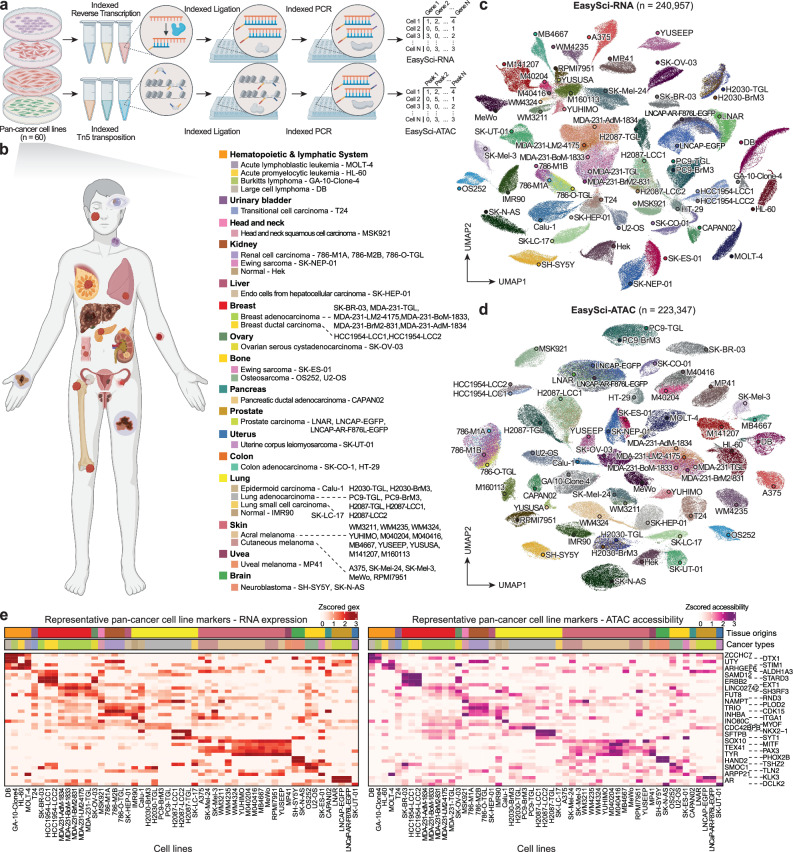
EasySci enables parallel transcriptome and chromatin accessibility profiling across diverse cancer cell lines. **a** Schematic of the EasySci-RNA and EasySci-ATAC library preparation workflows. Cancer cell lines were profiled separately for transcriptome and chromatin accessibility using combinatorial indexing strategies. Schematic in (**a**) is created in BioRender. Xu, Z. (2026) https://BioRender.com/l5c3t3d. **b** Overview of the 60 profiled cell lines, representing 20 cancer types and two non-malignant cell lines, a fetal lung fibroblast line (IMR-90) and an embryonic kidney epithelial cell line (HEK293). Notably, the panel included 11 AM cell lines. Schematic in (**b**) is created in BioRender. Ugurbil, A. (2026) https://BioRender.com/pm39zgk. **c**,**d** UMAP visualization of 240,957 single cells from 60 cell lines profiled by EasySci-RNA (**c**) and 223,347 single cells from 60 cell lines profiled by EasySci-ATAC (**d**), colored by cell line identity, with each cluster annotated by its corresponding cell line name. **e** Representative marker genes (*n* = 40) show concordant RNA expression and ATAC gene-body accessibility patterns specific to each cancer’s tissue of origin (*n* = 53).

To uncover cell-state heterogeneity and GRNs underlying distinct cancer cell states, we applied EasySci^13^ for single-cell RNA-seq and ATAC-seq analysis across sixty human cancer cell lines spanning twenty cancer types, including skin cutaneous melanoma (CM), acral melanoma (AM), breast cancer, colon adenocarcinoma, lung cancer, kidney renal cell carcinoma, hepatocellular carcinoma, and tumors associated with the hematopoietic and lymphatic systems, among others (Fig. 1a, b and Supplementary Data 1). Based on combinatorial indexing, EasySci enables parallel sample barcoding through indexed reverse transcription of different samples in separate wells of a plate. Subsequent split-pool barcoding exponentially expands the cell barcode space, enabling profiling of millions of cells in a single experiment. These features simplify multi-sample profiling and minimize technical batch effects^15^. Compared with previous single cell multi-omic analysis of cancer cell atlas that mostly focused on breast cancer and colorectal cancer^10^, our panel includes multiple cell lines for AM (WM3211, WM4235, WM4324, YUHIMO, M040204, M040416, MB4667, YUSEEP, YUSUSA, M141207, and M160113), CM (A375, SK-Mel-24, SK-Mel-3, MeWo, RPMI7951), and lung adenocarcinoma (H2030-TGL, H2030-BrM3, PC9-TGL, PC9-BrM3, H2087-TGL, H2087-LCC1, and H2087-LCC2), thus enabling the identification of gene regulatory circuits shared among cells of the same tumor types.

Nuclei were isolated from each cancer cell line and distributed into indexed wells for reverse transcription (EasySci-RNA) or transposition (EasySci-ATAC), allowing the cell identity to be directly retrieved based on each cell’s barcode. Following library preparation and sequencing, we recovered 240,957 single-nucleus transcriptomes (median 3784 nuclei per cell line; median 2351 unique transcripts per cell; Supplementary Fig. 1a, b, e) and 223,347 single-nucleus chromatin-accessibility profiles (median 3768 nuclei per cell line; median 7192 unique fragments per cell; Supplementary Fig. 1c, d, f).

UMAP embeddings of RNA or ATAC profiles showed that cells clustered primarily by cancer type and tissue of origin (Fig. 1c, d). In addition, representative marker genes of tissues exhibited concordant RNA expression and gene-body accessibility activations in subsets of cancer cell lines (Supplementary Data 2, 3), such as TYR and PAX3 in skin melanoma cells, which support melanogenesis and melanocytic/neural crest lineage identity^16^; NKX2-1 and SFTPB in lung carcinoma cells, which define lung epithelial identity and surfactant production^17,18^; STARD3 and ERBB2 in HER2-positive breast cancer cells, which mark the HER2-amplified locus and HER2 signaling^19^; KLK3 and AR in prostate cancer cells, which encode prostate-specific antigen and the androgen receptor TF, respectively^20^; and PHOX2B and HAND2 in neuroblastoma cells, which define sympathetic neuronal lineage identity^21^ (Fig. 1e). These results indicate that, despite being cultured under similar in vitro conditions, the cancer cell lines retain the gene expression and epigenetic signatures of their tissues of origin.

### Pan-cancer GRN analysis defines EMT-linked cell states shared across lineages

**Fig. 2:**
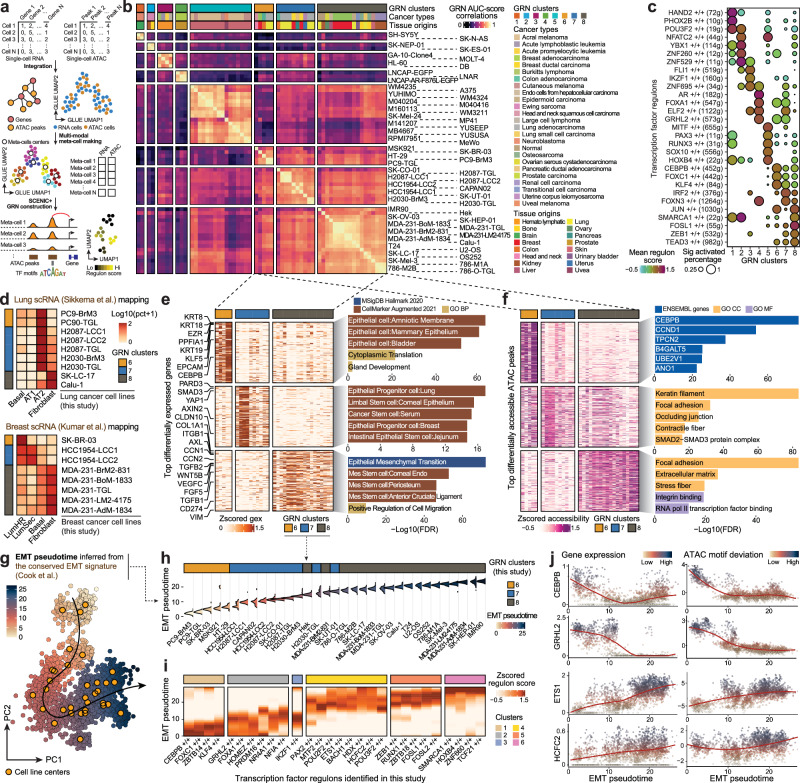
Pan-cancer gene-regulatory networks delineate intra- and cross-lineage molecular states de novo. **a** Schematic of pan-cancer GRN construction. Single-cell RNA-seq and ATAC-seq profiles were integrated, neighboring cells were aggregated into multimodal meta-cells, and GRNs were inferred by linking TFs to enhancers and nearby target genes. Regulon activity scores were then computed across meta-cells. **b** Hierarchically clustered heatmap showing correlations of TF-regulon AUCell scores across cell lines (*n* = 59), with annotations for GRN clusters, cancer types, and tissue origins. **c** Dot heatmap showing representative TF regulons (*n* = 29) activated across GRN clusters (*n* = 8). Dot size indicates the proportion of cell lines with significant regulon activation, and color indicates the mean gene-program expression score within each cluster. **d** Heatmaps showing the proportion of lung cancer (top, *n* = 9) and breast cancer (bottom, *n* = 8) cell lines mapped to phenotypically similar normal cell types (*n* = 4 for lung; *n* = 4 for breast) from external single-cell reference atlases. **e**,**f** Heatmaps showing top differentially expressed genes (*n* = 500 each group) (**e**) and differentially accessible ATAC peaks (*n* = 500 each group) (**f**) across cell lines in GRN clusters 6–8 (*n* = 5 for cluster 6, *n* = 10 for cluster 7, *n* = 18 for cluster 8), with functional-term enrichments shown on the right. **g** A PCA plot showing meta-cell distribution along the EMT trajectory inferred from a conserved EMT signature. Meta-cells (*n* = 2591, from 33 cell lines) are colored by inferred pseudotime, cell-line centers are highlighted, and the trajectory trend line was fitted using Slingshot. **h** A ridge plot showing EMT pseudotime distributions across cell lines in GRN clusters 6–8, with the top annotation indicating orthogonal-clustering identity inferred from our study. **i** Hierarchically clustered heatmap of gene program scores for EMT-associated TF regulons (*n* = 28) across the trajectory (n bin = 40). **j** Scatter plots with fitted trend lines showing coordinated gene expression and TF motif deviation across EMT pseudotime; points are colored by expression or motif deviation. Source data are provided as a Source Data file.

To dissect the transcriptional regulatory features across these phenotypically diverse cancer cells, we sought to identify gene cis-regulatory elements (CREs) and potential upstream TF regulators (Methods). EasySci-RNA and EasySci-ATAC profile transcriptomes and chromatin accessibility in separate cells, so no single cell carries both measurements. Given the balanced coverage across cell lines and RNA/ATAC modalities (Supplementary Fig. 1g), we performed computational integration across modalities using a meta-cell strategy. In this approach, single-cell transcriptomes and chromatin accessibility profiles were first integrated into a shared low-dimensional space, after which transcriptionally and epigenetically similar neighboring cells were identified and grouped to form meta-cells. Each meta-cell therefore represents the multi-modal profile of a local neighborhood of cells. By aggregating counts from locally similar cells, this approach links transcriptomic and chromatin states, reduces dropout-driven sparsity at each measurement point, and preserves cell-state heterogeneity at the meta-cell level (Fig. 2a, Supplementary Fig. 2). We then used these multi-modal meta-cells to identify ATAC peak–gene expression associations, perform TF motif scanning, and define regulons, here referring to groups of target genes co-regulated by a TF, to construct a pan-cancer GRN (Fig. 2a). Following rigorous data curation, consistent with prior studies^10,12^, we observed that cell-cycle status dominated intra-cell line heterogeneity at the multimodal level in most cases, whereas more discrete cell states were also robustly detected in specific cell lines (Supplementary Fig. 2a–j). In the pan-cancer GRN construction, we identified 85,670 linkages between 61,259 ATAC peaks and 13,295 target genes, with a median of five CREs regulating a gene (Supplementary Fig. 3a–c). Downsampling analyses further supported the importance of sufficient cell coverage and the robustness of network construction (Supplementary Fig. 3d–f).

To classify cell lines by their regulatory programs, we focused on activating regulons, defined as those in which TF expression is positively associated with target gene expression, and the accessibility of TF motif-containing cis-regulatory elements is also positively associated with target gene expression (Supplementary Fig. 3g, Supplementary Data 4, 5). Scoring pan-cancer cell lines with these 92 activating TF regulons, followed by hierarchical clustering, identified eight clusters of cancer cell lines with distinct gene regulatory programs (Fig. 2b), in which 5 (Clusters 1–5) were defined by the activity of tissues- and lineage-specific TFs. Cluster 1, consisting of SH-SY5Y and SK-N-AS neuroblastoma cell lines, exhibited strong activation of PHOX2B and HAND2 regulation, both well-established core factors in neurogenesis and neuroblastoma^21^. Cluster 2, consisting of the Ewing sarcoma cell lines SK-ES-01 and SK-NEP-01, showed high activity of YBX1, which has been reported to be essential for the malignant behavior of sarcoma^22^. Cluster 3 contained four hematopoietic and lymphatic system malignancy cell lines, including three lymphocytic cell lines (GA-10-clone4, MOLT-4, DB) and one promyelocytic leukemia cell line (HL-60), and was characterized by IKZF1, an essential TF in lymphocytic differentiation^23^. Notably, its aberrant activation in HL-60 reflected impaired myeloid differentiation^24^. Cluster 4, composed of prostate cancer cell lines, exhibited strong activation of AR and FOXA1, the master regulators of androgen signaling and prostate lineage identity^20^, while cluster 5, composed of melanoma cell lines, showed high activity of key melanocytic lineage-determining TFs, including MITF, SOX10, and PAX3^16^.

In contrast, clusters 6–8 contained a diverse mixture of cancer cell lines from distinct tissues and cancer types (Fig. 2b and Supplementary Data 6), including those from breast, lung, skin, head and neck, kidney, bone, colon, ovary, and uterus. This strongly suggests the existence of universal cell states that share similar gene-regulatory features beyond lineage specificity. The activation of TF regulons displayed a continuous transition across these clusters (Fig. 2c and Supplementary Data 7). For example, the activity of TFs that peaked in cluster 6, such as CEBPB and GRHL2, declined in clusters 7 and 8, while most TF regulons were highly active in cluster 8, including FOSL1, ZEB1 and TEAD3, which have been characterized to be involved in cancer dedifferentiation and EMT^25^, were relatively inactive in clusters 6 and 7. Furthermore, compared with clusters 6 and 7, cell lines in cluster 8 exhibited both the largest number of active TF regulons and the strongest overall transcriptional activation (Supplementary Fig. 3h–j), recapitulating the hypertranscriptional property of aggressive cancers^26,27^ and the stemness-associated transcriptional state^28^.

To further dissect the phenotypes of these pan-cancer-conserved cell states, we implemented a nearest-neighbor-based annotation label-transfer strategy. In brief, query cells from lung and breast cancer cell lines within clusters 6–8 were projected into a shared transcriptional space with reference single-cell RNA-seq atlases of normal lung^29^ and breast tissues^30^. Cell-type annotations were then assigned to query cancer cells based on the labels of their nearest neighbors among the reference cells (Fig. 2d, Methods). Consistent with the developmental scheme in which alveolar type 2 (AT2) cells serve as progenitors that differentiate into terminal alveolar type 1 (AT1) cells, and with recent findings^31^ identifying AT2 as the origin of lung adenocarcinoma, most lung cancer cell lines in clusters 6 and 7 originated from AT2 cells. In contrast, cluster 8 cells were mapped almost completely to a fibroblast phenotype (Fig. 2d, top). In the mammary tissue, luminal hormone-responsive (LumHR) cells represent a differentiated hormone-sensing state, whereas luminal secretory (LumSec) cells retain a more progenitor-like identity. A similar phenotypic transition was observed in breast cancer cell line mapping: SK-BR-3 mapped mainly to LumHR, consistent with a recent finding identifying LumHR cells as the origin of ER⁺ breast cancer^32^, while HCC1954 mapped to both LumHR and LumSec, reflecting higher plasticity. In contrast, triple-negative MDA-MB lines in cluster 8 exhibited a complete mesenchymal phenotype (Fig. 2d, bottom). Overall, the in vitro cancer cell lines from both tissues retained transcriptomic features of their cell-type of origin and, strikingly, their phenotypic transition across clusters 6–8 recapitulated the progression from specialized epithelial cells to a fibroblast-like mesenchymal state.

At the pan-cancer level, analysis of cluster-specific activated genes and ATAC peaks further supported these phenotypic assignments (Methods). Functional enrichment of differentially expressed genes confirmed that clusters 6–8 correspond to epithelial, intermediate, and mesenchymal states along the EMT trajectory (Fig. 2e). Consistently, accessible CREs in cluster 8 were enriched near genes associated with mesenchymal functions, such as stress fiber formation for stronger migratory ability of mesenchymal cells^33^. In contrast, the epithelial state showed strong chromatin activation at regulatory regions near genes, such as CEBPB. Notably, ATAC peaks that were significantly activated in cluster 7 showed partial activation in clusters 6 and 8, underscoring its role as an intermediate state along the EMT transition trajectory (Fig. 2f).

To further confirm the generalizability and robustness of our epigenetically and transcriptionally defined EMT-like states, we used a recently defined conserved EMT signature derived from diverse cell lines treated with pro-EMT cytokines^34^. This signature captures genes consistently induced across multiple EMT-promoting conditions and was used here as an orthogonal validation (Methods). Cells in our study aligned along a continuous trajectory defined by the published conserved EMT signature derived from distinct cancer cell lines^34^ (Fig. 2g), and their pseudotime distributions closely paralleled the GRN-based clustering independently defined by our epigenetic and gene expression association analysis (Fig. 2h). Further associating TF regulon activity to EMT progression revealed both established and new EMT regulators (Fig. 2j, Methods). In addition to more characterized EMT inducers (e.g., ZEB1^25^ and FOSL1^35,36^), ETS1 and HCFC2 also showed coordinated activation at the chromatin and transcriptional levels, accompanied by increased expression of their targets across pan-cancer EMT. Conversely, GRHL2, CEBPB, and FOXC1 showed epithelial-state-enriched expression and activity, with regulon activities negatively associated with EMT progression. These findings are consistent with previous studies in specific cancer models implicating GRHL2 in EMT suppression in breast, lung, and ovarian cancer, as well as CEBPB and FOXC1 in breast cancer^37–39^, and our analysis extends these cancer-type-specific observations to a broader pan-cancer context (Fig. 2j).

To validate the robustness of the regulatory phenotypes identified in our dataset, we trained a support vector machine model using the expression of pan-cancer GRN target genes to predict single-cell phenotype labels (Supplementary Fig. 3k). When applied to an external cancer cell-line single-cell transcriptomic dataset generated by Zhu et al. ^10^, this model successfully recovered pan-cancer EMT states in both overlapping cell lines (Supplementary Fig. 3l) and previously unseen cell lines annotated by Zhu et al. as occupying more differentiated epithelial or more mesenchymal states (Supplementary Fig. 3m), demonstrating that the transcriptional states and the gene-regulatory programs we define are reproducible across platforms.

Beyond inter-cell-line EMT plasticity, we also observed intra-cell-line plasticity, with cells from the same cell line spanning a limited range of EMT-associated states (Fig. 2h). We further confirmed that this reflected structured dynamics within each cell line, as EMT-high and EMT-low centers were significantly separated in the low-dimensional manifolds of most cell lines (Supplementary Fig. 4a–d). For example, in H2030-TGL (Supplementary Fig. 4d), cells in a more mesenchymal state formed a discrete cluster that could not be explained by technical or quality-related factors during preprocessing (Supplementary Fig. 2g–j). The ATAC-derived regulatory differences between these two clusters were also consistent with the EMT regulators identified by the pan-cancer GRN analysis (Supplementary Fig. 4e, f).

In summary, we defined core GRNs across diverse cancer cell types and identified both lineage-specific and lineage-independent EMT states, governed by conserved epigenetic and transcriptomic features.

### Copy number variation modulates the epithelial-mesenchymal cell state continuum

**Fig. 3:**
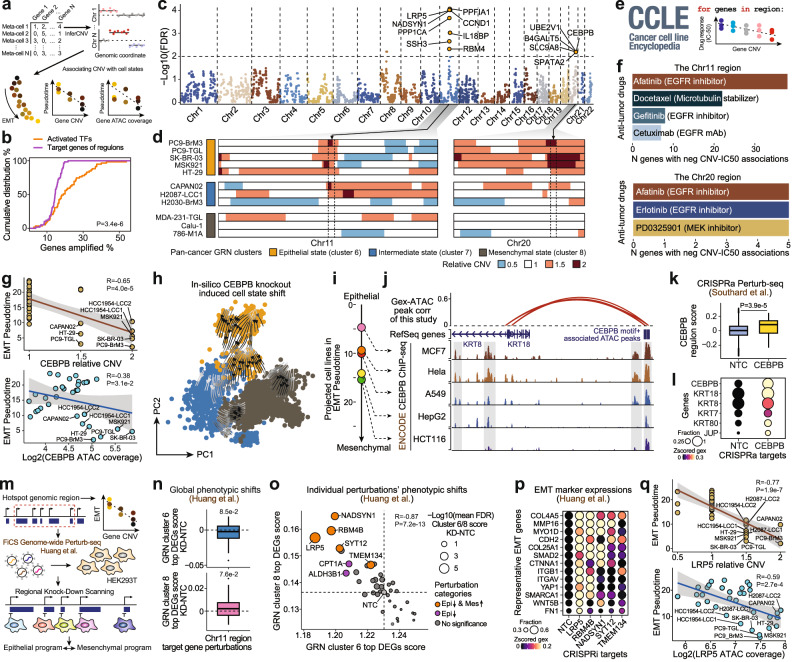
Pan-cancer copy-number analysis nominates cell states regulators. **a** Schematic of CNV calling and copy-number–EMT association analysis. **b** Cumulative distribution of amplified genes among activated TFs or activated-regulon targets across cancer cell lines; two-sided K-S test p-value is reported. **c** Gene-level CNV–EMT state associations across autosomes. Multiple-test-corrected FDR values from two-sided Pearson correlation p-values are displayed. **d** chr11 and chr20 CNV heatmaps in representative epithelial (*n* = 5), intermediate (*n* = 3), and mesenchymal (*n* = 3) cell lines; dashed lines mark EMT-associated CNV regions. **e** Schematic of CNV–drug-sensitivity association analysis using CCLE. **f** Top drugs with negative CNV–IC50 associations. **g** Negative associations of EMT pseudotime with CEBPB CN or normalized ATAC coverage. Shading denotes 95% confidence intervals; Pearson correlation coefficients and two-sided p-values are shown. N cell lines = 33. **h** PCA plot showing state-shift vectors after in silico CEBPB knockdown (metacell *n* = 2591, from 33 cell lines). **i** Median EMT pseudotime of external cell lines (N cells: MCF7 = 409, HeLa = 787, A549 = 1451, HepG2 = 1115, HCT116 = 543). **j** ENCODE CEBPB ChIP-seq tracks showing CEBPB-bound peaks proximal to KRT8 and KRT18. **k** CEBPB regulon scores in NTC (*n* = 13,673) and CEBPB CRISPRa RPE1 cells (*n* = 62); two-sided Wilcoxon test p-value is reported. **l** Z-scored expression of epithelial markers (*n* = 6) in NTC and CEBPB CRISPRa RPE1 cells. **m** Schematic of perturbation analysis. **n** Relative EMT state transitions after chr11-region gene knockdown (N perturbations = 39) compared with NTC; one-sided Wilcoxon test p-values are reported. **o** Significance and effect size of EMT state transitions for individual perturbations (*n* = 39). Multiple-test-corrected FDR values from two-sided Wilcoxon test p-values are reported. **p** Relative expression of EMT markers (*n* = 13) across perturbations significantly shifting EMT state (*n* = 5). N cells each perturbation from left to right: 218,838, 343, 261, 184, 246, 314. **q** Negative associations of LRP5 CN or normalized ATAC coverage with EMT pseudotime. Shading denotes 95% confidence intervals; Pearson correlation coefficients and two-sided p-values are shown. N cell lines = 33. Box plots show median, IQR, and 1.5× IQR whiskers. Source data are provided as a Source Data file.

As copy number variation (CNV) has been extensively recognized as a key driver of cancer cell behavior and is strongly associated with tumor response to therapies and patient prognosis^40^, we asked whether it also contributes to the cross-lineage cell-state alterations. We inferred genome-wide amplification and deletion patterns at the meta-cell level using InferCNV^41^ (Fig. 3a, Methods, Supplementary Data 8). As expected, no significant subclones were detected within individual cell lines, consistent with their continuous clonal evolution in culture. Cell lines with closer lineage proximity also displayed more similar CNV landscapes (Supplementary Fig. 5). In agreement with previous reports, the karyotypically normal diploid cell line IMR90 showed minimal CNVs (Supplementary Fig. 5a), and our inferCNV profiles were highly concordant with whole-exome- and whole-genome-sequencing-derived CNV patterns from independent studies^42–44^ (Supplementary Fig. 5c–e). By systematically examining the proportion of amplified genes among TFs with regulon activation and among the member genes of these regulons across all cell lines, we found that TFs were amplified more frequently than their downstream targets (Fig. 3b), suggesting that TF amplification and hyperactivation could represent a common mechanism governing cancer cell state heterogeneity.

To investigate the CNV landscape in the EMT process, we analyzed the significance of gene-level CNV-EMT pseudotime associations along genomic coordinates (Fig. 3a). In total, we identified 107 genes whose CNVs were significantly associated with pan-cancer EMT status, notably with exclusively negative correlations, indicating that amplification of these loci aligns with an epithelial cell state (Fig. 3c). As expected for genes affected by regional genetic alterations, these genes were concentrated in two specific genomic regions on chromosomes 11 and 20, respectively, which we refer to here as genomic hotspot loci (Fig. 3c, Supplementary Fig. 6a–h). Because ATAC chromatin accessibility reflects local DNA abundance, we used ATAC signal as an orthogonal proxy to validate transcriptome-derived CNV–EMT associations (Methods, Supplementary Fig. 6a-h, Supplementary Data 9). This approach highlighted 65 genes on chromosome 11 (e.g., PPFIA1, CCND, LRP5, NADSYN1, PPP1CA) and eight genes on chromosome 20 (e.g., CEBPB, B4GALT5, UBE2V1), where amplification correlated negatively with EMT status (Fig. 3c). Amplification of these regions occurred predominantly in cell lines within the epithelial states, whereas no amplification or deletion was detected in mesenchymal states (Fig. 3d). The finding is consistent with our prior analysis of differentially accessible ATAC peaks across pan-cancer GRN clusters (Fig. 2f). For example, peaks specifically activated in GRN cluster 6 were enriched for genes located within these regions, such as CCND1 and PTPN2 in the chromosome 11 hotspot, and CEBPB and UBE2V1 in the chromosome 20 hotspot (Fig. 2f, Supplementary Fig. 6a–h).

We next asked whether amplification of these genomic loci impacts drug response, given that cancer cell state is known to influence drug sensitivity^3,45^. Leveraging Cancer Cell Line Encyclopedia (CCLE)^46^, we associated CNV profiles of genes within these regions with IC50 values of anti-cancer drugs across large panels of cancer cell lines (Fig. 3e). By analyzing the number of genes whose amplification correlated with enhanced sensitivity (‘lower IC50’), we identified Afatinib as the top drug whose efficacy increased with amplification of most genes in both hotspot regions, followed by other EGFR and downstream inhibitors (Fig. 3f). This aligns with the established role of EGFR signaling as a key inducer of the EMT phenotype in cancer, and with prior evidence that EMT broadly confers resistance to EGFR pathway inhibitors^47–49^. Similarly, docetaxel, another drug previously reported to be resistant in EMT^50^, showed specific associations with a subset of genes in the chromosome 11 region (Fig. 3f).

Interestingly, CEBPB, a gene encoding a TF located in the chromosome 20 hotspot, was identified to be linked with the epithelial state of the EMT spectrum (Fig. 3c). When examining the association between inferred EMT pseudotime and the chromatin accessibility and gene expression-based CNV of CEBPB, it showed consistently increased ATAC accessibility and copy number amplification in epithelial-state lines, suggesting its hyperactivation as a potential mechanism preventing mesenchymal transition (Fig. 3g). As CEBPB was identified as a key TF within the pan-cancer GRN, we first used an in silico knockout strategy to examine how CEBPB ablation may affect cell state (Methods). In brief, SCENIC+ models gene expression based on the expression of inferred TF regulators and simulates perturbation-induced changes in gene expression by setting CEBPB expression to zero. This process was iterated to simulate the propagation of perturbation effects through the network. The simulated transcriptomes were then projected into the EMT PCA space to infer cell-state shifts. Consistent with our hypothesis, in silico CEBPB knockout induced a strong shift toward the mesenchymal state, specifically in epithelial-state cells (Fig. 3h). To test the generalizability of our analysis, we integrated paired CEBPB ChIP-seq^51^ and transcriptome profiles from external cancer cell lines (MCF-7, HeLa, A549, HepG2, and HCT116)^10^. By mapping these cell lines onto our inferred EMT trajectory (Methods), we estimated their EMT pseudotime and observed progressively reduced CEBPB binding at epithelial gene loci identified as CEBPB targets in our pan-cancer GRN, including KRT8 and KRT18^34^ (Fig. 3i, j). Finally, we leveraged CRISPRa Perturb-seq data from RPE1 epithelial cells with TF overactivation^52^ to test whether CEBPB activation is sufficient to enhance this regulatory program. Consistently, CEBPB activation led to increased activity of the CEBPB regulon identified from our pan-cancer atlas (Fig. 3k), including epithelial genes KRT18, KRT8, KRT7, KRT80, and JUP (Fig. 3l). Together, these orthogonal validations support a role for CEBPB in maintaining the epithelial identity of cancer cells.

In contrast, another hotspot region on chromosome 11 comprised a large cluster of genes but lacked major transcriptional regulators (Supplementary Fig. 6a). To distinguish potential drivers from passenger genes, we reanalyzed a published genome-wide CRISPRi Perturb-seq dataset^53^ that profiled epithelial cells with individual gene silenced, in which most genes within this region were perturbed (Fig. 3m and Supplementary Fig. 6j). To examine the potential contribution of these genes to EMT-associated state changes, we extracted top marker genes representing cell lines in GRN clusters 6 and 8 and defined them as epithelial and mesenchymal features, respectively. We then compared these phenotypic programs across individual gene perturbations relative to control cells. Mimicking the region-level negative CNV–EMT association, the aggregate perturbation effects showed an overall loss of epithelial features and activation of mesenchymal features (Fig. 3n). At the individual perturbation level, while most perturbations showed no observable effect (*n* = 31) or only partial effects on the epithelial program (*n* = 2), perturbation of LRP5, RBM4B, NADSYN1, SYT12, and TMEM134 (*n* = 5) induced both significant activation of the mesenchymal program and downregulation of the epithelial program relative to NTC cells (Fig. 3o). Strong induction of representative mesenchymal markers, including FN1, CDH2, and MMP16, further supported causal roles of these genes in maintaining the epithelial state (Fig. 3p). Notably, in our discovery dataset, these genes whose perturbation significantly altered epithelial and/or mesenchymal features in the Perturb-seq validation analysis (*n* = 7) exhibited stronger ATAC accessibility–EMT associations than the remaining genes in the region (Fig. 3q, Supplementary Fig. 6k).

In summary, our systematic pan-cancer CNV characterization highlights TF amplification, followed by their hyperactive transcriptional regulation, as a major mechanism shaping cancer cell states. CNV–EMT association analyses also indicate mechanistic heterogeneity: while CEBPB amplification reinforces epithelial identity via direct transcriptional regulation, regional amplifications also likely involve multiple genes with heterogeneous individual effects, whose combined dosage ultimately shapes the cell-state outcome. These distinct routes converge on cell-state modulation but also likely carry divergent implications for drug sensitivity.

### Subtype-resolved gene regulatory network in acral and cutaneous melanoma

We next assessed whether similar regulatory patterns are preserved in heterogeneous melanoma cells isolated from different patients, with a specific focus on AM. AM is a rare melanoma subtype with distinct epidemiology and prognosis^54^, but remains poorly characterized at the molecular level. To investigate its molecular signatures, we included diverse melanoma cell lines in our pan-cancer cell line cohort, which can be anatomically classified as uveal melanoma (*n* = 1), AM (*n* = 10 after quality control), and CM cell lines (*n* = 5) (Fig. 1b). Consistent with their melanocyte origin, 14 of 15 cell lines showed highly similar gene-regulatory features within the pan-cancer GRN, forming cluster 5 (Fig. 2b) and exhibiting stable lineage identity with high MITF and SOX10 activity (Fig. 2c).

**Fig. 4:**
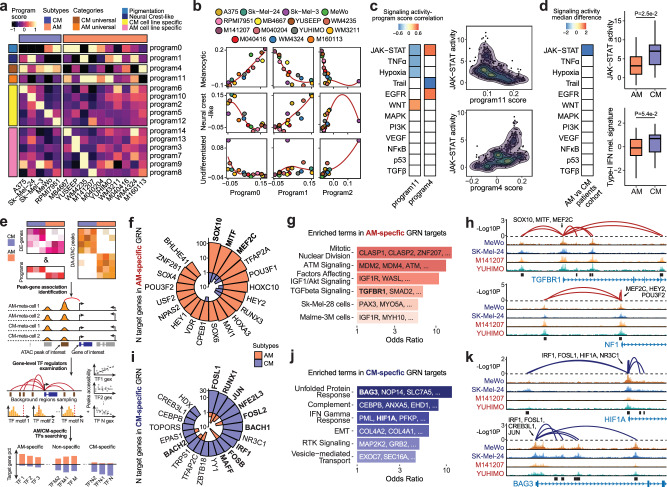
Integrated analyses define distinct gene-regulatory principles across melanoma subtypes despite phenotypic diversity. **a** Heatmap showing mean scores of gene programs (*n* = 15) deconvolved from melanoma cell lines (*n* = 15). **b** Scatter plots showing the ONMF-derived gene programs-established melanoma phenotypic signatures association. Each dot represents the mean score for a cell line; colors denote individual melanoma cell lines (*n* = 15). Loess trend curves were fitted. **c** Heatmap showing correlations between signaling activities (*n* = 12) inferred from meta-cell transcriptomes and AM/CM universal program scores across melanoma cell lines (left). Scatter plots with kernel density overlays show JAK–STAT activity versus program scores across meta-cells (*n* = 1015) (right). **d** Heatmap showing differences in signaling activities (*n* = 12) across AM (*n* = 42) and CM (*n* = 15) patients in the validation cohort (left). Only signaling activities with significant subtype differences are shown. Box plots show JAK–STAT activity and type-I IFN melanoma signature scores by subtype (right). Two-sided Wilcoxon test *p*-values are reported. **e** Schematic of melanoma subtype-specific GRN and TF analyses. **f** A rose plot showing AM-specific TFs and the number of their target genes within each DEG-refined subtype-specific program. TFs emphasized in the main text are bolded. **g** Bar plot showing functional-term enrichment among AM-specific TF target genes; representative genes are labeled. **h** Representative genome tracks showing pseudobulk-normalized ATAC coverage around TGFBR1 and NF1, genes universally upregulated in AM. Expression-associated peaks are boxed, CRE–gene linkages are shown as arches, and the highest points of arches indicate the permutation-based p-value of the association, and significant TF regulators of each CRE–gene pair are indicated. Example CM and AM cell lines are labeled in blue and red, respectively. **i** Rose plot showing CM-specific TFs and target-gene counts within each DEG-refined subtype-specific program. **j** Bar plot showing functional-term enrichment among CM-specific TF target genes; representative genes are labeled. **k** Representative genome tracks showing pseudobulk-normalized ATAC coverage around HIF1A and BAG3, genes universally upregulated in CM. Expression-associated peaks, CRE–gene linkages, and significant TF regulators are shown as in (**h**). Box plots show median, IQR, and 1.5× IQR whiskers. Source data are provided as a Source Data file.

To examine the molecular differences between AM and CM, we performed Orthogonal Non-negative Matrix Factorization (oNMF)^55^ in combination with differential expression analysis^56^ on the dataset (Methods, Supplementary Data 10). Extensive benchmarking demonstrated the robustness of the decomposed gene programs (Supplementary Fig. 7a–d). Differentially expressed genes between AM and CM recapitulated their most recognized divergence: CM displayed transcriptomic features of UV exposure, whereas AM, typically arising from sun-shielded skin, lacked such signatures (Supplementary Fig. 7e, f). Among the 15 oNMF-derived programs, 11 programs were strongly activated in individual cell lines (Fig. 4a), reflecting pronounced inter-cell line heterogeneity (Fig. 1c, d). Major phenotypic states of melanoma^57^, irrespective of subtype, were captured in this cohort: Program 0, enriched for pigmentation genes, such as MITF, TYR, OCA2, DCT, strongly associated with the melanocytic differentiation but inversely correlated with melanoma dedifferentiation (Fig. 4b), whereas Program 1, enriched for neural crest–associated genes, such as NGFR, CDH2, GDNF, GFRA1, mirrored the predefined neural crest-like state (Fig. 4b). Consistent with these associations, SK-Mel-3, the melanoma cell line with the lowest Program 0 score (Fig. 4b), exhibited the strongest expression of the undifferentiated signature and was the only melanoma cell line clustered within the cross-lineage mesenchymal cluster (cluster 8) in the pan-cancer GRN (Fig. 2b). Importantly, we observed that Programs 4 and 11 exhibited consistent activation in either CM or AM cells, revealing the existence of subtype-specific transcriptional modules distinguishing cutaneous and AM (Supplementary Fig. 7g). By overlapping the gene programs with genes differentially expressed between AM and CM, we identified the final AM- and CM-universal features (AM-universal genes, *n* = 1155; CM-universal genes, *n* = 1505) (Supplementary Fig. 7h, Supplementary Data 11). These signatures successfully separated AM and CM patients (Supplementary Fig. 7i). Moreover, as with cross-cancer EMT plasticity, subtle heterogeneity along an AM-like to CM-like spectrum was also observed among single cells within individual melanoma cell lines (Supplementary Fig. 7j–m).

By examining signaling activities across melanoma cell lines, although several pathway activities correlated with one of the DEG-refined subtype-universal gene programs, JAK–STAT signaling uniquely showed significant, but opposite associations in Programs 4 and 11 (Fig. 4c). Specifically, we found JAK-STAT signaling negatively correlates with the AM-universal program (R = −0.52, FDR = 3.68e-72) and positively with the CM-universal program (R = 0.53, FDR = 1.34e-76), which indicates an attenuated inflammatory phenotype in AM. Scoring bulk RNA-seq from a patient cohort containing both AM and CM^58^ further validated our observations: only JAK-STAT activity exhibited a significant reduction in AM relative to CM (Fig. 4d). Moreover, the score of a Type-I Interferon (IFN) signature, defining a subset of melanoma cells from patient samples^58^, was consistently lower in AM (Fig. 4d), indicating a universal inflammatory dampened state in this subtype.

We next explored the regulatory logic underlying these subtype distinctions by integrating refined AM- and CM-universal gene programs with subtype-specific differentially accessible ATAC peaks (AM-specific peaks, *n* = 63,510; CM-specific peaks, *n* = 40,862). This enables the construction of melanoma subtype-specific CRE–gene linkages and the identification of potential master TF regulators (Fig. 4e, Supplementary Fig. 7n, o, Methods, Supplementary Data 12, 13). Among 326 TFs identified to regulate at least 1 gene in melanoma, 21 were classified as AM-specific and 30 as CM-specific (Supplementary Fig. 7p, q).

Consistent with our prior characterization, melanocytic TFs predominated in the AM-specific GRN (Fig. 4f), which are known to suppress inflammatory gene programs^59,60^. Target genes of these TFs were enriched for the IGF pathway (Fig. 4g), previously implicated in specifically promoting AM pathogenesis^61^. Enrichment of ATM signaling further reflected pervasive genomic instability in AM, in contrast to the UV radiation-induced point mutations characteristic of CM^62–64^, consistent with our comparison of CNV patterns across melanoma subtypes (Supplementary Fig. 7r). The convergence of these features with transcriptomic signatures of MITF-dependent melanocytic cell lines, such as SK-Mel-28^65^ and Malme-3M^66^ (Fig. 4g), further supports the role of these TFs in shaping the gene-regulatory landscape of AM. For instance, TGFBR1, encoding the key receptor of the TGF-β pathway known to suppress intracellular inflammatory activity^67^, exhibited AM-specific upregulation through melanocytic TFs-associated CREs; NF1, a negative regulator of MAPK signaling, was activated through MEF2C-associated CREs in AM (Fig. 4h), adding a transcriptional regulatory layer to the mutual suppression between melanocytic and MAPK activities in melanoma^68^.

In contrast, the gene regulatory landscape of CM was characterized by extensive involvement of inflammatory TFs (Fig. 4i), dominated by AP-1 factors (FOSL1, JUN, FOSL2, FOSB), RUNX1, and CNC/bZIP factors (NFE2L3, BACH1, MAFF, BACH2) that share core motifs with AP-1. Notably, IRF1, an essential transcriptional effector of IFN stimulation and JAK-STAT signaling^69^, also emerged as one of the top CM-specific regulators (Fig. 4i), reinforcing the predominance of inflammatory transcriptional activity. Consistently, CM-specific target genes were enriched in inflammation-related functions, including the unfolded protein response and IFN responses (Fig. 4j). For example, the CRE associated with HIF1A and BAG3 displayed increased chromatin accessibility in several CM cell lines and were enriched for AP-1 and IRF1 motifs (Fig. 4k). Together, these findings suggest that AM exhibits a universal inflammation suppressive phenotype, while CM is represented by an inflamed phenotypic and gene regulatory landscape, between which JAK-STAT, along with downstream transcriptional response, might serve as a key player.

### From cancer-intrinsic programs to tumor microenvironment remodeling and prediction of immunotherapy response

**Fig. 5:**
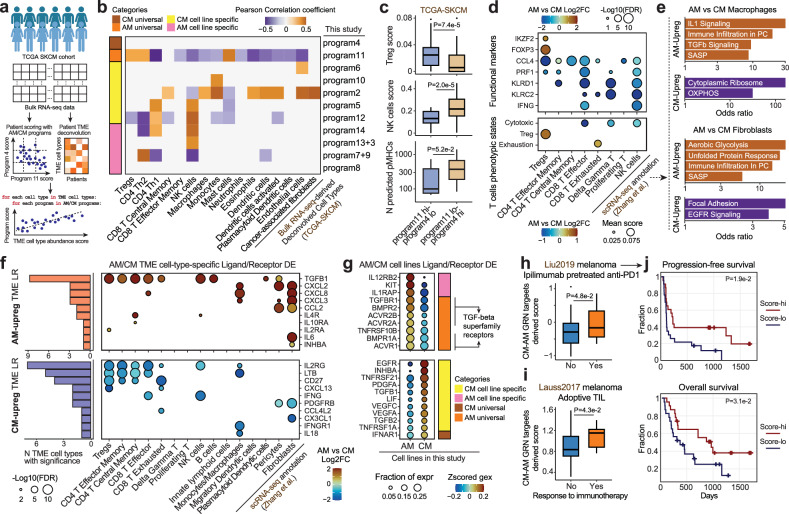
Subtype-resolved melanoma gene-regulatory signatures map TME states and immunotherapy sensitivity. **a** Schematic of analyses using TCGA-SKCM. **b** Correlations between melanoma gene-program scores (*n* = 11) and deconvolved TME cell-type scores (*n* = 16) across patient samples (*n* = 149). **c** Treg and NK-cell scores, and predicted MHC-binding peptide counts in TCGA-SKCM patients. Program 11 hi–program four lo patients, *n* = 46; Program 11 lo–program four hi patients, *n* = 56. For pMHC analysis, only samples with whole-genome sequencing were used: Program 11 hi–program four lo patients, *n* = 6; Program 11 lo–program four hi patients, *n* = 16. Two-sided Wilcoxon test p-values are reported. **d** Relative changes in T-cell functional markers (*n* = 7) and published T-cell signature scores (*n* = 3) across T-cell populations (*n* = 8) in AM (n patients = 6) versus CM (n patients = 3). Multiple-test-corrected FDR values from two-sided Wilcoxon test p-values are reported. **e** Top enriched terms among AM versus CM DEGs in macrophages and fibroblasts. **f** Relative expression changes of ligand/receptor genes (n AM-upregulated = 10, n CM-upregulated = 10) across TME cell types (*n* = 15) between AM (n patients = 6) and CM (n patients = 3). Numbers of cell types with significant differences are shown on the left. Multiple-test-corrected FDR values from two-sided Wilcoxon test p-values are reported. **g** Z-scored expression of significantly differentially expressed ligand/receptor genes (n AM-upregulated = 10, n CM-upregulated = 11) between AM (*n* = 10) and CM (*n* = 5) cell lines. **h**, Biomarker scores from CM-specific minus AM-specific target genes in ipilimumab-pretreated anti–PD-1 patient RNA-seq, comparing responders and non-responders (*n* = 17 responders; *n* = 29 non-responders). The two-sided Wilcoxon test p-value is displayed. **i** Same biomarker scores in pretreatment adoptive TIL therapy data, comparing responders and non-responders (*n* = 10 responders; *n* = 15 non-responders). The two-sided Wilcoxon test p-value is displayed. **j** Kaplan–Meier curves showing progression-free and overall survival for patients with high versus low biomarker scores (*n* = 23 high-score; *n* = 23 low-score). The two-sided Wald p-value for the signature term from univariate Cox regression is reported. Box plots show median, IQR, and 1.5× IQR whiskers. Source data are provided as a Source Data file.

Extensive studies have highlighted the central role of cancer cells in reprogramming their surrounding cellular niche through complex intercellular communication^70–72^. These adaptive changes in the tumor microenvironment (TME) can, in turn, substantially affect cancer cell behavior and therapeutic responses^73^. Given that inflammatory programs involve both intracellular and intercellular activities, we hypothesized that these key melanoma-intrinsic programs observed in vitro may be associated with distinct features of the original TME. To test this hypothesis, we explored bulk RNA-seq profiles from the TCGA SKCM cohort, in which the measured transcriptomes reflect both tumor-intrinsic and infiltrating TME compartments. We assessed associations between melanoma program scores, representing tumor-intrinsic states, and deconvolved TME cell-type scores, representing the microenvironmental compartment. This analysis revealed broad negative correlations between the AM-universal Program 11 and multiple immune-supportive populations across patients, including cytotoxic NK cells (R = −0.39, FDR = 1.5e − 5), CD8⁺ T effector memory cells (R = −0.25, FDR = 8.6e − 3), CD4⁺ Th1 cells (R = −0.63, FDR = 8.0e − 16), antigen-presenting dendritic cells (R = −0.39, FDR = 1.5e − 5), and endothelial cells (R = −0.43, FDR = 1.1e − 6), which facilitate tumor immune infiltration (Fig. 5b, c, Supplementary Data 14)^74^. In contrast, Tregs, a major immunosuppressive cell type in the TME, showed a positive association with Program 11 (R = 0.32, FDR = 4.1e − 4). These patterns are concordant with a previous single-cell study reporting reduced effector T cell infiltration and increased Tregs among tumor-infiltrating lymphocytes from AM patients^75^. These associations suggest that melanoma subtype-specific cancer-intrinsic programs detected in vitro are linked to distinct TME features in patient tumors. Moreover, after stratifying TCGA SKCM samples by AM- and CM-universal program scores (Fig. 5c), we found significantly lower predicted MHC-I neoantigen burden^76^ in AM-like samples (Program 11-high, Program 4-low) than in CM-like samples (Program 11-low, Program 4-high). This is consistent with the high UV-driven tumor mutation burden of CM^77^, and the relatively immunosuppressive TME landscape associated with AM.

To evaluate potential confounding of bulk melanoma subtype-associated program scores by tumor purity or TME composition, we performed additional analyses using patient melanoma single-cell RNA-seq data^58^ and TCGA-SKCM cohort (Supplementary Fig. 8). Both Programs 4 and 11 were strongly enriched in melanoma cells relative to non-malignant TME compartments (Supplementary Fig. 8a). TCGA-SKCM samples generally showed high tumor purity^78^ (Supplementary Fig. 8b), and the bulk scores of these cancer-intrinsic programs were positively associated with estimated tumor purity (Supplementary Fig. 8c). Moreover, residual Program 11 gene scores showed no change in TME cell types between AM and CM patients (Supplementary Fig. 8d), supporting the interpretation of Program 11 as a melanoma-cell-derived program.

To further validate the suppressive microenvironment of AM at a molecular phenotype level, we further analyzed published single-cell RNA-seq of TME of both AM and CM patient samples^58^ (Supplementary Fig. 9a–c). Across cell types, T cell populations exhibited the most pronounced transcriptomic changes (Supplementary Fig. 9d, Supplementary Data 15). By comparing the expression of functional markers in T cell populations between AM and CM patients, we observed a global attenuation of antitumor immunity in AM (Fig. 5d). For example, Tregs in AM showed higher expression of IKZF2 and FOXP3, consistent with enhanced suppressive function. In contrast, PRF1 and IFNG, which encode core cytotoxic effector molecules and cytokines, showed markedly lower expression in CD8 effector T cells and NK cells in AM. By further quantifying phenotypic changes using recurrent T cell state signatures^79^, we found that Tregs and exhausted CD8 T cells from AM patients showed increased immunosuppressive/exhaustion-associated programs, whereas CD8 effector T cells, γδ T cells, and NK cells showed reduced cytotoxic programs (Fig. 5d). Other major cellular lineages in the TME also exhibited convergent immunosuppressive features. For example, macrophages from AM patients showed enrichment of IL1 signaling and senescence-associated secretory phenotype (SASP) programs, consistent with a tumor-associated macrophage (TAM)-like phenotype that may contribute to an immunosuppressive TME^80^. Fibroblasts from AM patients also showed an increased SASP secretion program, together with reduced focal adhesion and EGFR signaling, suggesting a shift toward an immunosuppressive cancer-associated fibroblast (CAF)-like state rather than a classic CAF phenotype^81^. Overall, these cell-type-specific transcriptomic profiles of the AM TME complement our melanoma gene program–TME composition inference and further substantiate a suppressive microenvironment aligned with the uninflamed melanoma cell state as a defining characteristic of AM.

To uncover candidate mediators linking cancer cells and TME, we compared ligand/receptor expression between AM and CM across both patients’ TME and melanoma cell lines (Fig. 5e, f). In TME, TGFB1 emerged as the most consistently upregulated ligand/receptor gene in AM, activated across nine TME cell types, whereas others were mostly cell-type-specific (Fig. 5e). Across melanoma cell lines, TGF-β superfamily receptors exhibited universally activated expression in AM versus CM, and were identified as members of the AM universal gene program (Program 11). Among them, TGFBR1, encoding the receptor of TGF-β1, and downstream effectors, such as SMAD2, were actively induced through the AM-specific GRN (Fig. 4j–k). Analysis of paired ligand/receptor expressions across compartments via CellPhoneDB^82^ showed a consistent trend, highlighting the potential central role of TGF-β in reprogramming TME and AM (Supplementary Fig. 9e, f). Consistently, TGF-β signaling is well established as a major suppressive axis in the TME: it inhibits cytotoxic programs of CD8⁺ T cells and NK cells, impedes CD4⁺ Th1 differentiation, downregulates MHC-II in dendritic cells, and enhances Treg activity through FOXP3 induction^83^.

Conversely, in CM, the most consistently upregulated ligands/receptors within the TME were IL2RG, LTB, and CD27 across T-cell populations, highlighting an activated antitumor milieu. The cancer cell compartment exhibited a parallel interactome landscape: while most cytokines upregulated in CM were cell-line-specific, particularly within the highly dedifferentiated SK-Mel-3 (Supplementary Data 16), IFNAR1, which encodes the Type-I IFN receptor, was the only significantly upregulated ligand/receptor within the CM universal gene program, consistent with CM’s inflamed TME, elevated intracellular JAK-STAT signaling activity, and an inflammatory GRN (Fig. 4i–k).

Finally, because TME is a strong predictor of immunotherapy outcomes, we evaluated whether melanoma subtype-specific gene-regulatory features could predict immunotherapy response. Using the Cancer Immunology Data Engine (CIDE)^84^, which integrates pretreatment RNA-seq profiles and clinical metadata from immunotherapy cohorts, we tested AM- and CM-universal gene signatures as biomarkers of therapeutic response. Although the full AM and CM universal programs did not distinguish responders from non-responders (Supplementary Fig. 10a, b), incorporating gene-regulatory information by refining these programs to target genes of AM- and CM-specific TFs (Fig. 4e–k) markedly improved performance. These refined GRN-based scores significantly separated responders from non-responders from the same cohorts (Fig. 5h, i). Moreover, because anti-CTLA-4 and anti-PD-1 therapies are standard immunotherapeutic approaches for melanoma^85^, we further examined the prognostic potential of our melanoma subtype signatures (Supplementary Fig. 10c). Consistently, full AM and CM universal programs did not distinguish treated patients’ prognosis (Supplementary Fig. 10d). In anti-CTLA-4-naive patients treated with anti-PD-1, none of the pre-established immune signatures significantly predicted prognosis by univariate Cox regression, with the strongest-performing signature, IMPRES^86^, showing two-sided Wald P values of 0.111 for PFS and 0.122 for OS (Supplementary Fig. 10e, f). In contrast, the GRN-based scores specifically stratified prognosis in anti-CTLA-4-pretreated patients subsequently treated with anti-PD-1, achieving predictive performance comparable to established immune signatures and stronger than tumor mutation burden (Fig. 5i). Validation in additional cohorts^87,88^ further supported the generalizability of this finding (Supplementary Fig. 10h–k). This association remained significant for overall survival in multivariable Cox regression adjusting for age and sex (Supplementary Fig. 10k). Together, these results underscore the close association between melanoma-intrinsic regulatory logic and therapy outcomes, particularly in the anti-CTLA-4 to anti-PD-1 treatment sequence.

Together, these analyses demonstrate that key melanoma gene regulatory modules are stably maintained in vitro, faithfully reflect tumor-TME co-adaptation in vivo, and link to immunotherapy responsiveness. Integrating single-cell and bulk transcriptomic data across model and patient contexts highlights how multi-omic dissection of cancer gene regulation can advance precision oncology.

## Discussion

The complexity of cancers has long hindered a unified understanding of their underlying molecular features and the development of targeted therapeutics. Here, we leveraged a comprehensive cancer cell line resource to dissect gene regulatory principles and convergent molecular features of cancer. Using our custom EasySci single-cell platform with highly parallel inter-sample processing capacity^13,89^, we performed single-cell transcriptomic and epigenetic profiling of 60 cell lines spanning 16 tissue origins and 24 cancer types. Compared with the previous study that profiled single-cell transcriptomes and chromatin accessibility across cancer cell lines^10^, we expanded RNA profiling by more than 10-fold and obtained over 4-fold more single-cell ATAC profiles, covering more tissue origins and cancer types and enabling a more unbiased characterization of intra- and inter-lineage variation.

Beyond prior single-modal examinations of transcriptomic phenotypes in cancer cells^12,90^, our cross-modal dataset enabled integrative analyses linking chromatin landscapes with transcriptomes to identify gene regulatory phenotypes driven by differential activation of TF-regulated gene expressions^91^. The observation that these GRN-based cancer cell line clusters either reflect tissue origin and cancer type or contain highly heterogeneous cell lines is consistent with previous pan-cancer integrative clustering on bulk patient samples across copy number, DNA methylation, mRNA, and miRNA profiles^92^, in which both single cancer-type-dominant and mixed clusters were identified. This reveals the coexistence of tissue-specific molecular programs reflective of developmental origins and convergent features shared across lineages. The diversity we observed in cell lines further supports the maintenance of key transcriptional phenotypes of cancer under in vitro conditions, highlighting their utility for drug-response measurement and mechanistic dissection^93^.

Regarding phenotypic convergence across cancers, extensive characterization of cross-lineage cancer cell line clusters supports their reflection of EMT^25,94^, reinforcing the existence of a universal transcriptional dedifferentiation program across epithelial cancers, with EMT representing the dominant cross-lineage phenotypic spectrum. Although the universality of EMT across cancer types has been widely debated^34,95,96^, our de novo identification of this phenotypic spectrum at the pan-cancer level provides strong supporting evidence. Moreover, while a few TFs, such as ZEB1, have long been recognized as essential EMT drivers^25^, recent evidence suggests a more complex mode of action involving cooperative regulation by multiple TFs, including AP-1, TEADs, and ZEB1^36^. The incomplete phenotypic reversal observed upon inhibition of single TFs further supports the involvement of additional factors^97,98^. Beyond ZEB1, FOSL1, and FOSL2 identified here, other strong EMT-associated TFs nominated from our study may expand this repertoire, offering potential avenues for combinatorial inhibition. Conversely, the reidentification of epithelial-state-associated TFs, including GRHL2, CEBPB, and FOXC1, further supports the convergence of EMT-associated regulatory programs across cancer types. While these factors have been implicated in EMT regulation in specific cancer contexts^37–39^, our pan-cancer single-cell analysis places them within a broader cross-lineage framework, highlighting recurrent epithelial-associated regulatory features shared across diverse cancer cell states. These analyses provide additional mechanistic insight into the regulatory dynamics underlying cancer-associated EMT.

Previous studies have widely identified TF amplification as a key event in carcinogenesis^99^; for example, MYC focal amplification through ecDNA formation enhances cellular fitness under microenvironmental stress^100^. Recent work exemplified how chromosomal amplification can alter cancer cell states through TF overactivation, as shown in a case study of SOX4 in glioblastoma subclones^9^. Here, we systematically identified preferential amplification of TFs, rather than their target genes, as a common phenomenon across cancers. Our association and validation analyses further identified CEBPB as a TF that promotes the epithelial state through copy-number amplification, thereby extending this TF-amplification-driven regulatory mechanism to more benign cell states. Moreover, our analyses nominated another set of genes whose amplification may constrain EMT through transcription-independent mechanisms. Similarly, a previous study identified tumor-suppressive amplification of chromosome 21 linked to hyperactivity of calcineurin suppressors^101^. Such mechanistic diversity underscores the need for caution in therapeutic design despite the convergence of cancer cell states^102^.

Given the abundance of melanoma cell lines in our cohort, we conducted extensive comparative analyses to identify gene regulatory features specific to AM compared with the more common  CM. Only limited comparisons have been made between these subtypes, primarily focusing on genomic alterations^62^ and their therapeutic outcomes^103^. Complementarily, we explored their differences in gene regulation. By deconvolving gene programs and constructing GRNs for each subtype, we found that JAK-STAT signaling activity and inflammatory transcriptional responses represent the primary distinctions. Notably, many TFs we identified as melanoma subtype-specific are also key regulators of melanoma phenotypic switching, including SOX10 and MITF in melanocytic differentiation and FOSL1 and JUN in the development of the undifferentiated state^104^. As our orthogonal gene program deconvolution presents these subtype-independent phenotypic states in parallel, the enrichment of these TFs within subtype-specific regulatory architectures strongly suggests their multifaceted roles in tuning coexisting phenotypes in melanoma.

Lastly, by extrapolating gene regulatory features derived from cancer cell lines to their corresponding TME characteristics, we found that the AM gene program is associated with an immune-suppressive TME composition, consistent with prior spatial transcriptomic studies of AM showing a globally low level of immune infiltration^105^. Through integrated analysis of patient-derived TME cell-type-specific transcriptomes and melanoma cell lines, we identified TGF-β1 as a potential key reprogramming factor shaping the AM TME, whereas CM was characterized by active anti-tumor immunity and a potentially heightened IFN response. As previously reported, TGF-β signaling robustly attenuates tumor response to PD-L1 blockade^106^, suggesting its potential as a therapeutic target in the context of AM. Furthermore, our subtype-specific GRN successfully classified patients by immunotherapy response, consistent with observations from large clinical cohorts showing that AM exhibits lower response rates and poorer prognosis than CM^103,107^.

Although we profiled 60 cell lines spanning 20 cancer types, our panel does not encompass the full diversity of human cancers. Several clinically important malignancies, such as pancreatic, gastric, and bladder carcinomas, were not represented, and therefore, the extent to which the regulatory programs identified here generalize to these contexts remains to be determined. Future studies incorporating broader cancer type coverage, additional genetic backgrounds, and primary tumor or patient-derived models will be important for defining which regulatory programs are broadly conserved versus lineage- or context-specific.

A second limitation is that our study profiles cancer cell lines cultured in isolation, which do not fully recapitulate the complex TME, including immune, stromal, endothelial, and fibroblast components, as well as the extracellular matrix. Therefore, our dataset is best interpreted as a resource for dissecting cancer cell-intrinsic regulatory programs. Nevertheless, cell lines provide a tractable and controlled system that minimizes confounding from variable tumor purity, inter-patient heterogeneity, and tissue composition, factors that can complicate analyses of primary tumors, especially for rare cancer subtypes with limited available specimens. The retention of key phenotypic states and regulatory programs in vitro supports the utility of these models for studying intrinsic cancer cell states. Although our analyses suggest that the AM- and CM-associated programs primarily reflect melanoma-cell-enriched transcriptional states, variation in tumor purity and TME composition may still contribute to program scoring in bulk tumors.

In addition, ligand-receptor inference should be interpreted as an exploratory analysis for hypothesis nomination. Our patient-level tumor-microenvironment inferences also relied primarily on a single AM single-cell cohort with limited clinical annotation, and larger, more completely annotated cohorts will be needed to assess how ethnicity, treatment history, and other clinical variables shape TME composition and cell states. Future integration with subtype-resolved single-cell, spatial transcriptomic, or imaging-based datasets will further distinguish malignant cell-intrinsic programs from microenvironment-associated components and strengthen mechanistic understanding of how specific TME phenotypes are established and maintained.

Overall, this single-cell multi-omics resource and our analyses comprehensively delineate pan-cancer gene regulatory principles and reveal diverse modes of altering cancer cell states. By uncovering the TME-coadapted gene regulatory landscape of melanoma cell lines in vitro, our findings highlight the fidelity of in vitro cancer models in recapitulating clinically relevant regulatory programs and point to the potential of transforming molecular multi-omics insights into clinical interpretation and translational application.

## Methods

### Ethics statement

This study was conducted in accordance with all relevant ethical regulations. All cell lines used in this study were obtained from established commercial or academic repositories. The study did not involve human participants, human specimens, animal experiments, or clinical data requiring institutional review board approval.

### Cell culture

For the AM cell lines: YUHIMO, YUSEEP, and YUSUSA were obtained from Yale University. M040204, M040416, M141207, and M160113 were obtained from the University of Zurich. WM3211, WM4325, and WM4324 were obtained from the Wistar Institute. MB4667 was obtained from the University of Colorado. The remaining cell lines (HCC1954-LCC1, HCC1954-LCC2, MDA-231-BrM2-831, MDA-231-LM2-4175, MDA-231-AdM-1834, MDA-231-BoM-1833, MDA-231-TGL, SK-BR-03, H2030-BrM3, H2030-TGL, H2087-TGL, H2087-LCC1, H2087-LCC2, PC9-BrM3, PC9-TGL, Calu-1, SK-LC-17, SH-SY5Y, SK-N-AS, 786-M1A, 786-M2B, 768-O-TGL, CAPAN02, LNAR, LNCAP-EGFP, LNCAP-EGFP-c.2-F876L, HT-29, SK-CO-01, SK-OV-03, SK-UT-01, SK-HEP-01, MSK921, OS252, SK-ES-01, and SK-NEP-01 cell lines) were obtained from the Antibody and Bioresource Core Facility at Memorial Sloan Kettering Cancer Center. IMR90 (CCL-186), MeWo (HTB-65), RPMI7951 (HTB-66), Sk-Mel-24 (HTB-71), Sk-Mel-3 (HTB-69), DB (CRL-2289), GA-10-Clone-4 (CRL-2393), MOLT-4 (CRL-1582), HL-60 (CCL-240), U2-OS (HTB-96), and MP41 (CRL-3297) cell lines were obtained from the American Type Culture Collection (ATCC). All cell lines were maintained at 37 °C with 5% CO₂, and cultured in the required culture media as shown in Supplementary Data 1. Cell lines were authenticated by checking the morphology and mapping sequencing reads from different cell lines to species-specific reference genomes. All cell lines used in this study were routinely tested for mycoplasma contamination using a PCR-based assay (Universal Mycoplasma Detection Kit, 30–1012 K, ATCC) and were mycoplasma-negative.

### EasySci RNA library preparation

Libraries were prepared following the published EasySci-RNA protocol^13^. Primer DNA was synthesized by IDT. In brief, nuclei were isolated from cultured cell lines and distributed at up to 20,000 nuclei per well across two 96-well plates in Nuclear Suspension Buffer. Reverse transcription used dual priming including 50 µM short-dT (/5Phos/ACGACGCTCTTCCGATCTNNNNNNNN[10 bp cell barcode]TTTTTTTTTTTTTTT) and 50 µM randomN (/5Phos/ACGACGCTCTTCCGATCTNNNNNNNN[10 bp cell barcode]NNNNNN) with Maxima H- Minus Reverse Transcriptase and a staged temperature ramp (55 °C denaturation followed by 4 °C–55 °C gradient). Post-RT, nuclei were pooled, washed, and redistributed for plate-specific barcoding by DNA ligation with pre-annealed ligation adapters (A*G*A*T*C*G*G*A*A*G*A*G*C*G*T*C*G*T*G*T*A*G*G*G*A*A*A*G*A*G*T*G*T*/3ddC/). Second-strand synthesis (NEB Ultra II Non-Directional) was performed, followed by 0.8× AMPure cleanup. Libraries were then tagmented with loaded Tn5, quenched with SDS/BSA, and indexed by addition of universal P5 primer (AATGATACGGCGACCACCGAGATCTACAC) and indexed P7 primer (CAAGCAGAAGACGGCATACGAGAT[10 bp i7]GTCTCGTGGGCTCGG), followed by amplification with NEBNext High-Fidelity 2× Master Mix (12–15 cycles determined by qPCR when applicable). Final libraries were purified, quantified by Qubit, size-checked on 2% agarose, and sequenced on Illumina NextSeq or NovaSeq (paired-end 100 bp; Index 1/2: 10 bp each).

### EasySci ATAC library preparation

Libraries were prepared following the published EasySci-ATAC protocol^13^. In brief, nuclei were isolated from cultured cell lines and approximately 5000 nuclei per reaction were subjected to indexed Tn5 transposition (N5 oligo: /5Phos/ACGACGCTCTTCCGATCT[6 bp N5 barcode]AGATGTGTATAAGAGACAG, N7 oligo: CGTGTGCTCTTCCGATCT[6 bp N7 barcode]AGATGTGTATAAGAGACAG) in 20 mM Tris‑HCl, 20 mM MgCl₂, and 20% DMF for 30 min at 37°C, followed by quenching with 1× EDTA stop buffer. Tagmented nuclei were pooled, redistributed across 96‑well plates, and barcoded via plate‑specific P5 ligation (A*G*A*T*C*G*G*A*A*G*A*G*C*G*T*C*G*T*G*T*A*G*G*G*A*A*A*G*A*G*T*G*T*/3ddC/). After pooling and cleanup, libraries were amplified with universal P5 (AATGATACGGCGACCACCGAGATCTACAC) and indexed P7 (CAAGCAGAAGACGGCATACGAGAT[10 bp i7]GTGACTGGAGTTCAGACGTGTGCTCTTCCGATCT) primers using NEBNext High‑Fidelity 2× PCR Master Mix. Final libraries were column-purified and gel-extracted, and sequenced on Illumina NextSeq or NovaSeq (100‑cycle kit; Read 1: 58 cycles, Read 2: 60 cycles, Index 1/2: 10 cycles each).

### Sequencing data processing

We used custom computational pipelines for processing EasySci-RNA and -ATAC data as previously described in ref. ^13^. In brief, for the RNA modality, UMI and cell barcode sequences from each read pair were extracted from specific locations within the reads. Adapter trimming on Read 2 was performed using Trim Galore with default settings to remove poly(A) sequences and low-quality bases from the 3′ end. The paired-end sequences were then aligned to the genome with STAR, and PCR duplicates were removed using both UMI and genomic coordinate information. Reads were subsequently split into single-cell SAM files, and gene expression counts were generated.

For the ATAC modality, N5, N7 Tn5 barcodes and cell barcodes were extracted, and paired-end reads were aligned with STAR after adapter trimming with Trim Galore. Only uniquely aligned read pairs were retained, and PCR duplicates were removed using Picard at the PCR-well level. BED files containing mapped fragment start and end sites, strand information, and cell barcodes were generated for downstream processing.

Scanpy (v1.11.0)^108^ was used for single-cell RNA-seq analysis. Scrublet (v0.2.3)^109^ was applied for doublet identification. To visualize RNA profiles of single cells, gene counts were normalized and log1p-transformed, followed by selection of the top 5000 most variable genes. Regression on total cell counts and subsequent scaling of HVG expression across cells were performed. The top 30 principal components (PCs) were then used to construct the nearest neighbor graph and the global UMAP.

SnapATAC2 (v2.8)^110^ was used for single-cell ATAC-seq fragment demultiplexing and downstream analyses. PCR-well-level BED files were indexed and used as inputs. Only cells with at least 1000 fragments and a TSS enrichment (TSSE) score ≥ 3 were retained. Peak calling was performed at the cell line level using the macs3 function, and peaks overlapping with the ENCODE human genome blacklist^111^ were removed. Significant peaks (q < 1e − 3) were iteratively merged. The single-cell ATAC peak count matrix was constructed using the make_peak_matrix function, and the global UMAP was computed using the top 30 spectral embeddings derived from the 300,000 most variable peaks.

### Single-cell RNA-ATAC integration

Single-cell RNA-ATAC integration was performed using GLUE (v0.3.2)^112^. Because our pan-cancer cell line cohort included multiple cell lines with high transcriptomic similarity, integration was carried out at the individual cell line level. In brief, for each cell line, a preprocessed AnnData object containing a raw single-cell gene expression count matrix with the top 3000 highly variable genes and PCA embedding, and an ATAC AnnData object containing the spectral embedding from the top 300,000 peaks were generated using Scanpy and SnapATAC2, respectively, and provided as input to GLUE. A guidance graph was constructed based on the genomic proximity of ATAC peaks to annotated genes using the reference genome annotation. To improve integration quality, Leiden clustering and UMAP visualization were performed. Clusters consisting predominantly of cells with low RNA UMI counts, low ATAC fragment counts, or low TSSE scores were removed, and a second round of integration was performed using the refined cell sets. Integration quality was evaluated both manually and computationally. Manual assessment was conducted by visualizing cell cycle scores in the GLUE-derived co-embedding UMAP, while integration consistency scores from GLUE were computed in parallel to provide a quantitative measure of integration accuracy.

### Multi-modal meta-cell identification

Multi-modal meta-cell identification was performed using SEACells (v0.3.2)^113^. For each cell line, the final GLUE co-embedding was used to construct kernels, and meta-cells containing at least five cells from both modalities, with each modality contributing 20–80% of the total number of cells, were retained for downstream analyses. The expected number of meta-cells per cell line was estimated as (total number of cells)/75. For each meta-cell, raw gene expression counts and ATAC peak counts from all assigned cells were aggregated. For cell lines with poor integration quality, specifically, those with co-embeddings that failed to capture clear cell cycle progression (often associated with low overall UMI counts), or with severe imbalance between RNA and ATAC cell numbers (log2(RNA cell number / ATAC cell number) > 3 or <–3), all cells were collapsed into a single meta-cell.

### Pan-cancer gene regulatory network construction and regulon-based analyses

Pan-cancer–level TF–cis-regulatory element–target gene triplets were identified using SCENIC+ (v1.0a2)^114^. In brief, meta-cell RNA profiles were preprocessed with Scanpy, and the raw count matrix was stored in the.raw slot. Meta-cell ATAC profiles were preprocessed using pyCisTopic^115^, and the top 35 topics were selected based on the diagnostic metrics implemented in the package. Candidate peaks were defined by combining (i) the top 3000 peaks with the maximum probability of belonging to each topic, (ii) peaks identified within each topic using Otsu thresholding, and (iii) highly variable peaks that were significant markers of each cell line. A custom cisTarget motif library was then built using candidate peaks with ±1 kb extension as background padding. Linkage identification and AUCell scoring at the meta-cell level were performed using the SCENIC+ Snakemake pipeline.

Given the more established role of TFs in transcriptional activation and the greater number of activation regulons, we retained only multimodal regulons showing positive associations between TFs and target genes, as well as between ATAC peak accessibility and target genes (+/+). For each cell line, the mean AUCell score of each regulon was calculated, and hierarchical clustering was performed to identify GRN clusters across cell lines.

To assess cell-line–specific regulon activation, Wilcoxon rank-sum tests were performed on regulon scores (computed with the score_genes function in Scanpy) between meta-cells from the focal cell line and the remaining meta-cells. For cell lines that had been pseudobulked into a single sample for GRN construction, RNA-only meta-cells were generated by randomly sampling 50 cells per meta-cell. Regulons were considered active in a given cell line if they met an FDR threshold of 0.01 and had a mean regulon score at least 0.1 greater than the background.

### Differential gene expression and differential peak accessibility analyses and functional enrichments

The pan-cancer cell line multi-modal marker examination was conducted using the rank_genes_groups function in Scanpy. Only genes expressed in at least 200 cells were retained for the analysis. Marker examinations on ATAC were performed similarly using the normalized, log1p-transformed gene activity, obtained by summarizing ATAC signal coverage across gene body regions. The top significant (FDR <1e-3) cell line markers with the highest log2 fold changes in both modalities were further intersected.

To further include the single-cell data sparsity into consideration and minimize the false positive discovery^116^, DESeq2^56^ was used to examine GRN-cluster-specific differentially-expressed genes at the meta-cell level. As there is no single universal differentially accessible peak identification method that consistently outperforms the rest of them, the marker_regions function in snapATAC2 was used to identify GRN-cluster-specific differentially accessible peaks. Only peaks with counts in at least 2.5% cells and showing absolute log fold change >= 0.25 were considered. Functional enrichment on DEGs was conducted using Enrichr^117^; GREAT was used for functional enrichments on genomic regions^118^.

### EMT trajectory inference and trajectory-associated regulon analysis

A curated gene list of conserved upregulated and downregulated genes during EMT, identified from cytokine-treated cell line panels^34^, was used to order cells in low-dimensional space. PCA on these genes across all RNA meta-cells was performed, and the root of the transition trajectory was determined by scoring these genes. Slingshot (v2.16.0)^119^ was then applied to infer pseudotime along the trajectory.

To identify pan-cancer regulons associated with EMT, generalized additive models (GAMs) were fitted using the gam() function from the mgcv R package, modeling regulon gene program scores as a function of meta-cell pseudotime. Each GAM was compared to a null model using the anova() function, and regulons with significantly improved fits were considered pseudotime-dependent. To evaluate directionality, pseudotime was divided into 30 bins, and the mean regulon scores were correlated with mean pseudotime across bins. Background distributions were generated by shuffling pseudotime across meta-cells. Regulons with Pearson correlation coefficients > 0.4 or < −0.4 and FDR < 0.01 were designated as positively or negatively associated, respectively. As an additional filter, the expression of the TF itself was modeled against pseudotime using GAM; only regulons whose TFs showed significant pseudotime dependence and had correlations > 0.4 between TF expression and regulon score were retained as final regulators.

ATAC motif deviations of TFs were computed using chromVAR (v1.30.1)^120^. Motif matches within ATAC peaks were identified using the matchMotifs() function, and motif deviations were computed relative to GC-matched background peaks using the computeDeviations() function. To ensure comprehensive coverage, position weight matrices (PWMs) of motifs included in the SCENIC + GRN construction but absent from the default JASPAR database were manually merged into a custom supplementary motif database for chromVAR.

### Copy number variation inference and association analyses

InferCNV (v1.24.0)^121^ was used to call CNVs at the meta-cell level based on RNA raw counts. We adopted a reference-free mode to avoid background overcorrection. Gene coordinate files were generated from the reference genome annotation used for read mapping, and RNA meta-cells were grouped by cell line identity. Genes were ordered by genomic position, and normalized expression values were smoothed across neighboring genes within each chromosome to identify broad regional expression shifts relative to the cohort-wide pseudo-reference baseline. Then the hidden Markov model (HMM) of inferCNV, the 6-HMM model, produced six CNV states: 0 = complete loss, 0.5 = single-copy loss, 1 = neutral, 1.5 = single-copy gain, 2 = two-copy gain, and 3 = more than two-copy gain. Bayesian post-filtering of HMM-defined CNV regions was performed using a default posterior normal-probability cutoff of 0.5, and genomic regions with CNVs supported by posterior probabilities > 0.5 were retained. Retained CNV events were further evaluated based on regional consistency and orthogonal ATAC/WGS/WES support.

To identify associations between EMT status and gene-level CNVs across pan-cancer cell lines, Pearson correlation coefficients were computed between pseudotime-inferred EMT status and gene copy number states derived from the HMM model. Multiple testing correction was performed across all genes using FDR.

To further validate “hotspot” regions associated with EMT, ATAC-seq coverage across gene bodies was extracted, and correlations with EMT status were computed in the same manner. Only genes that showed consistent and significant correlation trends at both the RNA-inferred CNV and ATAC coverage levels were considered.

### Validation analysis on CEBPB by in-silico knockout and ChIP-seq

SCENIC + ^114^ was used to simulate the perturbation effects of CEBPB. In brief, target genes of each regulon together with the RNA meta-cell count matrix were input into the train_gene_expression_models() function to construct random forest models, modeling the expression of target genes as a function of upstream regulators. CEBPB expression was then set to zero, and 10 iterations of perturbation simulation were performed using the simulate_perturbation() function. The resulting simulated transcriptomic states with CEBPB expression eliminated were projected onto the EMT PCA embedding of meta-cells using the plot_perturbation_effect_in_embedding() function.

To map additional cell lines (MCF7, HeLa, A549, HepG2, HCT116) onto the EMT trajectory, we reprocessed single-cell RNA-seq data for these lines^10^, which were selected because ENCODE^51^ provides CEBPB ChIP-seq profiles for them. To minimize potential biases from data sparsity differences, single cells from these datasets were randomly sampled and aggregated into meta-cells of 20 cells each, given their deeper sequencing depth. After normalization, meta-cell count matrices from the internal and external datasets were combined, retaining only genes from the conserved EMT gene sets described above. Genes were then scaled across all meta-cells. The get.knnx() function from the FNN R package was used to identify the two nearest neighbors of each external meta-cell within the scaled internal meta-cell matrix. The mean EMT pseudotime of these two neighbors was assigned to the external meta-cell, and the median transferred EMT pseudotime was used to represent the EMT status of each external cell line. Finally, CEBPB-to-background p-value signal tracks from ENCODE ChIP-seq experiments were visualized using the IGV tool (v2.19.6)^122^.

### Gene program deconvolution and signaling activity scoring on skin cancer cell lines

The oNMF module of D-SPIN (v1.4.0)^55^ was used to deconvolve orthogonal gene programs from single-cell RNA-seq profiles of skin cancer cell lines. Manual curation of gene program members was performed, and the number of programs chosen was determined based on those yielding the most biologically interpretable results. To further refine core gene programs, differential gene expression analyses on meta-cells were conducted in parallel using DESeq2. For programs 4 and 11 (AM/CM universal programs), member genes were intersected with significant DEGs between AM and CM cell lines (FDR < 0.01). For other AM/CM cell line–specific programs, member genes were intersected with significant DEGs comparing the given cell line to all other cell lines (FDR < 0.01). Meta-cell program scores were computed using the score_genes function in Scanpy.

Signaling activities of meta-cells were estimated using PROGENy (v1.17.3)^123,124^, which infers pathway activity by weighting predefined “footprint” genes. Hormone-associated pathways (estrogen and androgen) were excluded from analysis.

### Examination of AM/CM-specific transcriptional regulation

For finer-grained skin cancer subtype–specific GRN construction, we constrained the set of genes and ATAC peaks examined in FigR (v0.1.0)^125^. Only genes from DE-refined program 4 and program 11, and peaks showing significantly different accessibility between AM and CM cell lines (identified using SnapATAC2; only peaks detected in ≥1% of cells and with absolute log fold change ≥ 0.25 were considered) were used for peak–gene association. For each candidate gene, 100 permutations were performed to compare the observed peak–gene correlation with correlations generated from GC-matched background peaks. Genes with at least four associated peaks were further analyzed for TF regulation by testing (i) TF motif over-representation in the associated peaks and (ii) correlation of candidate TF expression with accessibility of these peaks. Only positive TF regulators whose expression significantly correlated with peak accessibility (p < 0.1) and whose motifs were significantly enriched in the same peaks (p < 0.1) were retained as master regulators of the peaks and their downstream target genes.

As inputs included only AM-specific genes (DE-refined program 11) and CM-specific genes (DE-refined program 4), we further quantified TF specificity. For each TF, the proportion of AM- or CM-specific target genes among all AM- or CM-specific genes targeted by all TFs was calculated. Proportion tests with FDR correction across all TFs were then conducted to identify AM- and CM-specific TFs exerting significantly stronger regulation on larger sets of target genes in a given skin cancer subtype.

### Bulk and single-cell RNA-seq TME analyses

Bulk RNA-seq count files from the melanoma cohort of TCGA^126^ were downloaded by selecting Program: TCGA, Project: TCGA-SKCM, and Primary Site: skin. Bulk RNA-seq–based tumor microenvironment (TME) immune cell type deconvolution results were obtained from TIMER2.0^127^. Patients’ bulk RNA-seq profiles were scored against DE-refined skin cancer gene programs using the AddModuleScore() function in Seurat^128^. Pearson correlations were computed between program scores and TME cell type abundances across patients, followed by FDR correction of correlation p-values. Predicted neoantigen counts derived from TCGA patient SNV profiles were obtained from ref. ^76^. Patients were grouped, and Wilcoxon tests were applied to compare metrics across groups.

Single-cell RNA-seq datasets profiling the TME of both AM and CM patients were obtained from ref. ^58^. Patient-level AnnData objects were concatenated, and standard single-cell processing was performed, including normalization, log1p transformation, highly variable gene selection, regression on gene number, PCA, nearest-neighbor graph construction, UMAP embedding, and Leiden clustering. Melanoma cell clusters characterized by high MITF expression and patient-specific clustering patterns were removed. TME cell types in MITF- or PTPRC+ clusters with contributions from multiple patients were retained for an additional round of processing and doublet removal using doubletdetection^129^. Clusters with doublet proportions >30% were excluded. Final cluster annotation was performed using a combination of representative marker gene expression and CellTypist^130^, an automated cell type annotation tool. Key immune cell phenotypic signatures were scored using starCAT (v1.0.9)^79^, and comparisons between AM and CM conditions were conducted using Wilcoxon tests followed by FDR correction. Differential expression results for key functional markers and ligand/receptor genes were extracted from global differential expression analyses of each TME cell type between AM and CM conditions. Ligand/receptor genes were selected from the KEGG database^131^ under the term “CYTOKINE CYTOKINE RECEPTOR INTERACTION”.

Patient immunotherapy response and survival data were obtained from the Cancer Immunology Data Engine (CIDE)^84^. AM target genes of AM-specific TFs were used as negative biomarkers, while CM target genes of CM-specific TFs were used as positive biomarkers. Biomarker scores for patients were computed from pretreatment transcriptomic data in the database and used for patient stratification.

### Statistics & reproducibility

The study profiled 60 human cancer cell lines representing 16 tissue origins and 20 cancer types, yielding 240,957 single-nucleus RNA-seq profiles and 223,347 single-nucleus ATAC-seq profiles after quality control. Median nuclei per cell line were 3784 (RNA) and 3768 (ATAC). No statistical methods were used to predetermine sample size. No data was excluded. The profiling experiment was conducted in six batches. Cancer cell lines were selected to represent diverse tissue origins and cancer types. No randomization was performed because the experiments profiled established cell lines without allocation to intervention groups. Nuclei from each cell line were processed independently for single-cell RNA-seq and ATAC-seq using combinatorial indexing, and computational analyses were performed uniformly across all samples. Blinding was not applicable to this study. Experiments involved profiling established cancer cell lines without subjective outcome assessment. Computational analyses were performed using objective statistical criteria. For external patient cohort analyses, group annotations were based on publicly available metadata.

### Reporting summary

Further information on research design is available in the Nature Portfolio Reporting Summary linked to this article.

## Supplementary information

Supplementary Information

Description of Additional Supplementary Information

Supplementary Data

Reporting Summary

Transparent Peer Review file

## Source data

Source Data

## Data Availability

The single-cell RNA and ATAC raw FASTQ data generated in this study have been deposited in the NCBI BioProject database under accession code PRJNA1354039. Processed gene count matrices, ATAC peak count matrices, and cell metadata generated in this study have been deposited in the NCBI Gene Expression Omnibus (GEO) database under accession code GSE311521. Public reference lung and breast tissue single-cell RNA-seq atlases data were retrieved via CZ Cell x Gene portal [https://cellxgene.cziscience.com/]. Public CNV and drug sensitivity data for cell lines were obtained from the Cancer Cell Line Encyclopedia [https://sites.broadinstitute.org/ccle/]. Public ChIP-seq data were obtained from ENCODE. The TF-ome CRISPRa Perturb-seq on RPE-1 cell line was obtained from Zenodo [https://zenodo.org/records/15213619]^52^. Public immunotherapy patient cohort data were collected by CIDE^84^. The conserved EMT signatures used in this study are available in the manuscript [https://www.nature.com/articles/s41467-020-16066-2]^34^. The FiCS HEK293T Genome-wide Perturb-seq data used in this study is available in the FigShare database [https://plus.figshare.com/articles/dataset/Processed_data_for_X-Atlas_Orion_Genome-wide_Perturb-seq_Datasets_via_a_Scalable_Fix-Cryopreserve_Platform_for_Training_Dose-Dependent_Biological_Foundation_Models/29190726]^53^. Subtype-resolution patients’ melanoma single-cell RNA-seq used in this study are available in the GEO database under accession code GSE215121^58^. The bulk RNA-seq of a SKCM cohort used in this study are available in the TCGA database [https://portal.gdc.cancer.gov/]^126^. Source data are provided with this paper, the remaining data are available within the Article, Supplementary Information or Source Data file. Source data are provided with this paper.

## Code availability

Analysis scripts used in this study are available at GitHub [https://github.com/JunyueCaoLab/Cancer_atlas].
