# Supplementary material for: Single-cell multimodal profiling of pan-cancer cell lines uncovers gene regulatory principles underlying intrinsic cell states and environmental features
Source: Nat Commun. 2026 Jul 23;17:6975. doi: 10.1038/s41467-026-75360-7 (PMC13396447; doi:10.1038/s41467-026-75360-7)
Supplement: Supplementary file 1 — Supplementary Information [file 41467_2026_75360_MOESM1_ESM.pdf]

Supplementary Information

Supplementary Figure 1 1
Supplementary Figure 2 3
Supplementary Figure 3 5
Supplementary Figure 4 7
Supplementary Figure 5 8
Supplementary Figure 6 10
Supplementary Figure 7 12
Supplementary Figure 8 14
Supplementary Figure 9 15
Supplementary Figure 10 17
Supplementary Reference 19

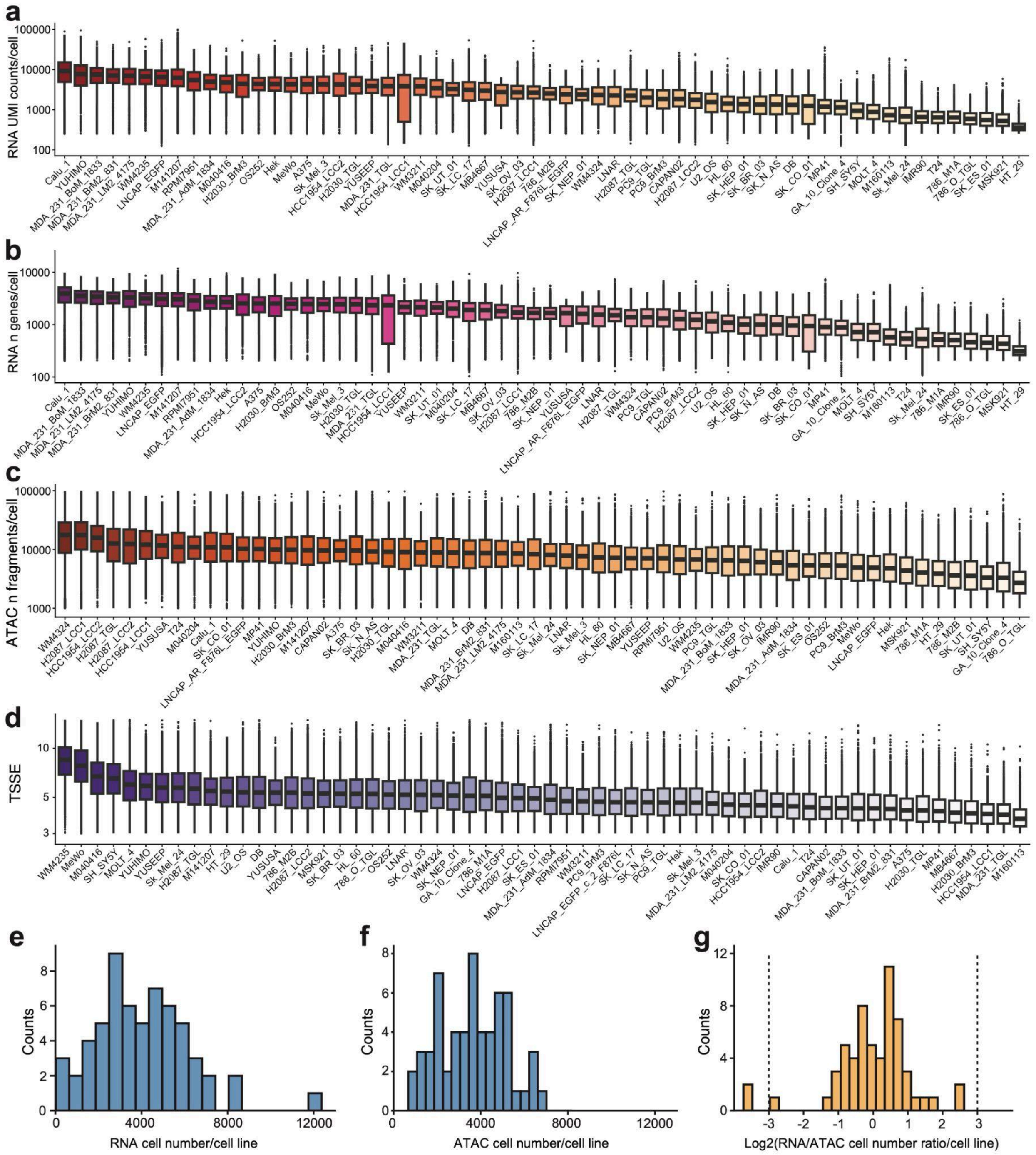

Supplementary Figure 1. Quality metrics of pan-cancer cell line multi-modal single-cell profiling.

a, Box plot showing the distribution of single-cell RNA UMI counts across cell lines. **b,** Box plot showing the distribution of the number of genes detected per single cell across cell lines. **c,** Box plot showing the distribution of the number of ATAC fragments per single cell across cell lines. **d,** Box plot showing the distribution of single-cell TSSE scores across cell lines. **e,** Histogram showing the distribution of single-cell RNA-seq cell numbers across cell lines. **f,** Histogram showing the distribution of single-cell ATAC-seq cell numbers across cell lines. **g,** Histogram

showing the distribution of log2 RNA-to-ATAC cell numbers across cell lines. Dashed lines indicate the cutoffs used for filtering. Only cell lines without extreme imbalance between RNA and ATAC cell numbers were retained for integration. N per group in a-d are the number of cells each cell line profiled in this study. The detailed single-cell meta-data is uploaded to GEO. Box plots show median, IQR, and 1.5× IQR whiskers.

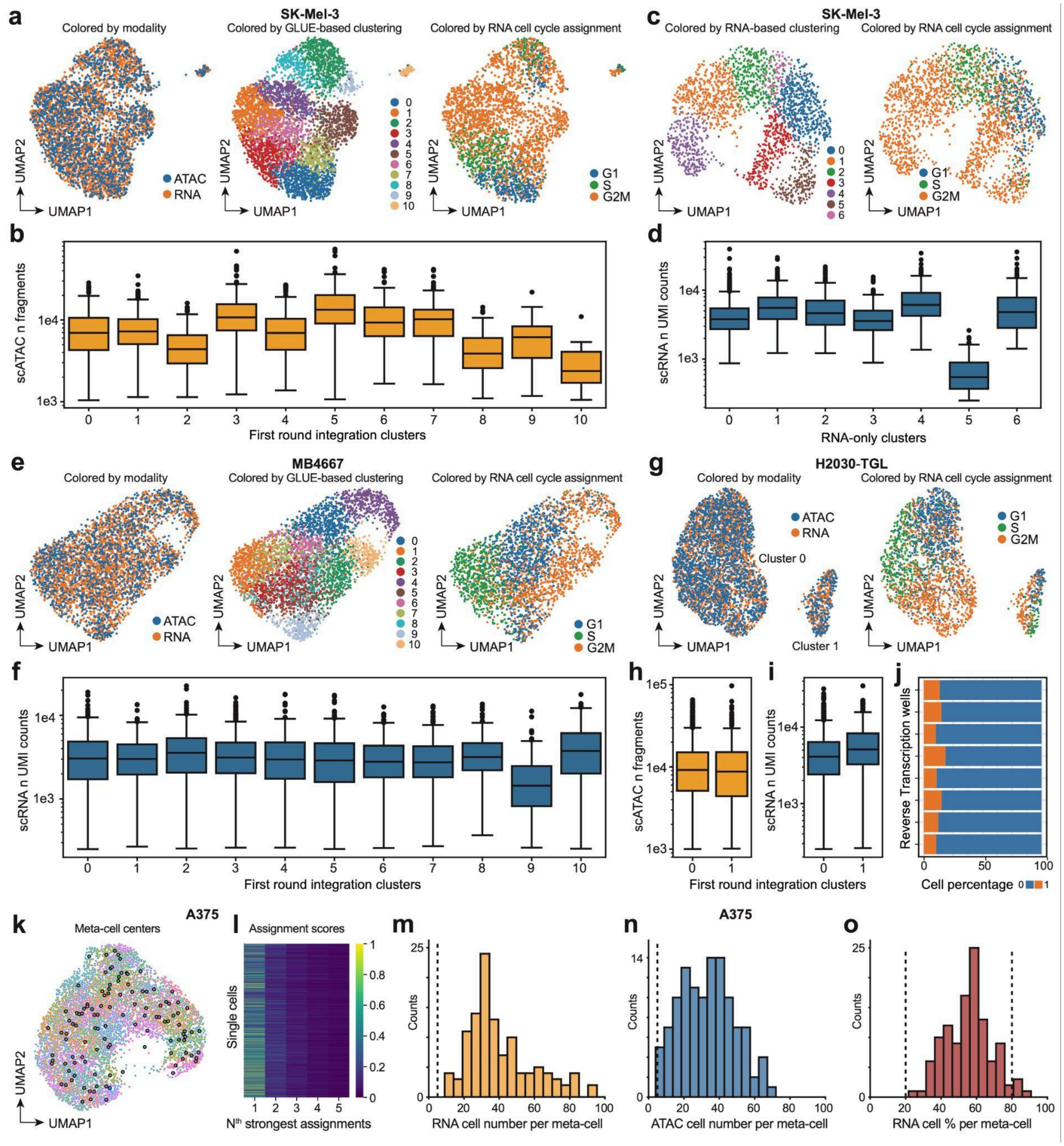

Supplementary Figure 2. Integration and processing of single-cell RNA and ATAC data and meta-cell identification.

a, Example of ATAC-driven low-quality cell filtering: first-round GLUE UMAPs of SK-Mel-3 cells (cell line n = 1, n RNA cells = 2,877, n ATAC cells = 1,734) colored by modality, Leiden cluster, and RNA-based cell-cycle phase. **b,** Box plot showing single-cell ATAC fragment distributions across SK-Mel-3 Leiden clusters (N ATAC cells from cluster1 to 10: 240, 238, 211, 191, 189, 176, 176, 129, 117, 45, 22). Outlier clusters 2, 8, 9, and 10 showed mixed cell-cycle assignments and low ATAC quality. **c,** RNA expression-based UMAPs of SK-Mel-3 cells colored by Leiden cluster and cell-cycle phase after removing low-quality cells and first-round integration

outliers (cell line n = 1, n RNA cells = 2,877). **d,** Box plot showing RNA UMI distributions across expression-based Leiden clusters (N RNA cells from cluster1 to 5: 546, 463, 327, 306, 276, 200, 99); outlier cluster 5 contained low-quality RNA cells. **e,** Example of RNA-driven low-quality cell filtering: first-round GLUE UMAPs of MB4667 cells (cell line n = 1, n RNA cells = 2,950, n ATAC cells = 1,516) colored by modality, Leiden cluster, and cell-cycle phase. **f,** Box plot showing RNA UMI distributions across MB4667 Leiden clusters (N RNA cells from cluster1 to 10: 376, 349, 337, 293, 283, 256, 245, 242, 220, 181, 168). Outlier cluster 9 showed mixed cell-cycle assignments and low RNA quality. **g,** Example of a true discrete intra–cell line state: first-round GLUE UMAPs of H2030-TGL cells (cell line n = 1, n RNA cells = 2,532, n ATAC cells = 2,434) colored by modality and cell-cycle phase. This discrete state was also observed in an independent study. **h, i,** Box plots showing ATAC fragment numbers and RNA UMI counts across main and outlier H2030-TGL clusters (N RNA cells of cluster1 and 2: 2,214, 318; N ATAC cells of cluster1 and 2: 2,207, 227), indicating that the outlier was not explained by low ATAC or RNA quality. **j,** Bar plot showing RT-well composition of both clusters, indicating that the outlier was not explained by library-preparation batch effects. **k,** Example of meta-cell identification: final GLUE UMAP of A375 cells (cell line n = 1, n RNA cells = 4,763, n ATAC cells = 3,829) colored by meta-cell assignment, with meta-cell centers highlighted by black-circled dots. **l,** Heatmap showing the top five SEACells assignment scores for A375 single cells (n cell = 8,592; n cell line = 1). **m–o,** Histograms showing the number of RNA cells, number of ATAC cells, and percentage of RNA cells per A375 meta-cell. Box plots show median, IQR, and 1.5× IQR whiskers.

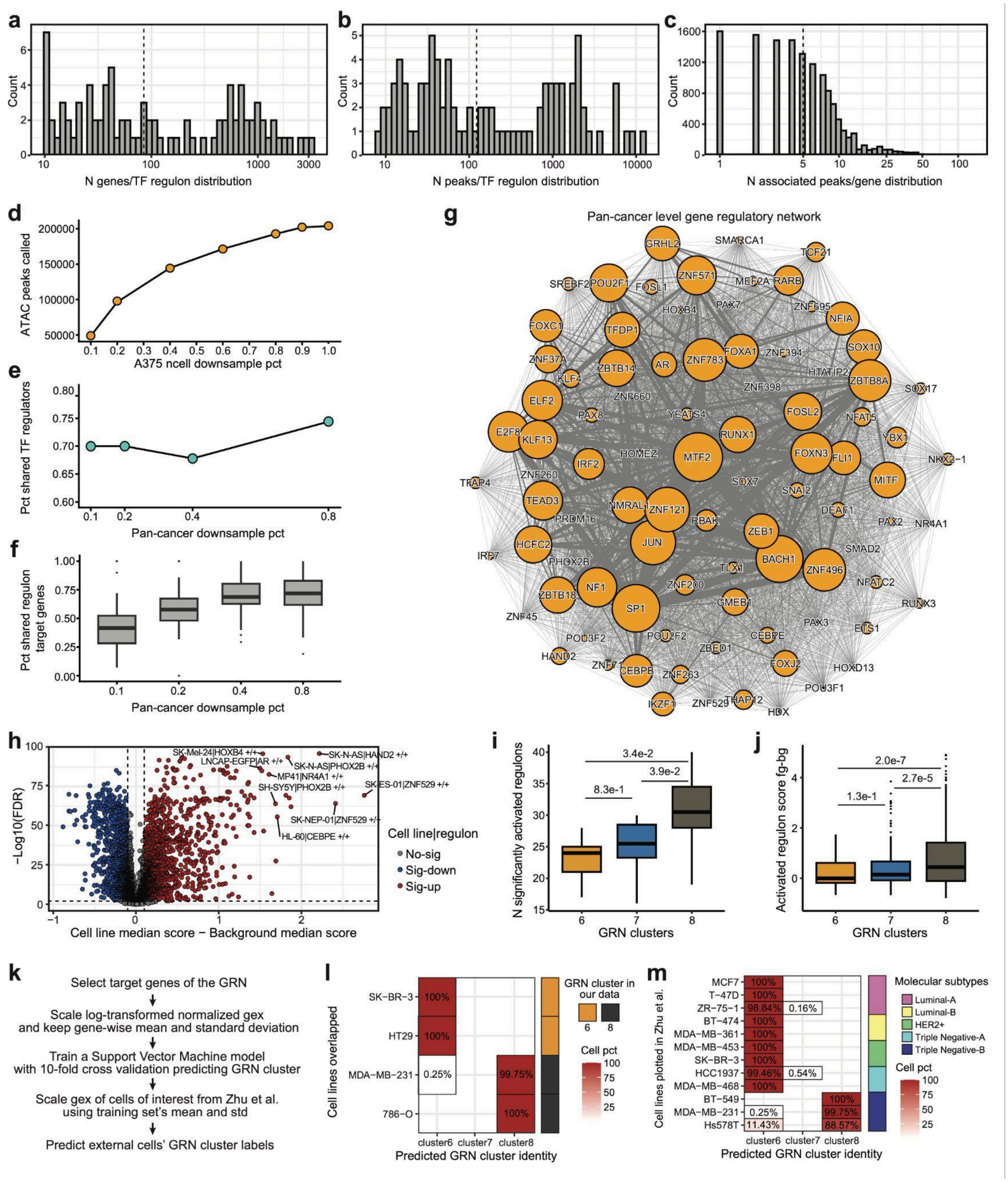

Supplementary Figure 3. Characteristics of the pan-cancer gene-regulatory network.
a–c, Histograms showing the distributions of the number of target genes per TF (a), ATAC peaks regulated by TFs (b), and ATAC peaks associated with downstream genes (c). Dashed lines indicate medians of 85, 122.5, and 5, respectively. **d,** Number of ATAC peaks called from A375 across increasing cell downsampling fractions. **e, f,** Fractions of TF regulators (e) and regulon target genes (N regulons from 0.1 to 0.8 groups: 51, 52, 47, 50) (f) recovered relative to

the full dataset across pan-cancer downsampling fractions. **g,** Network diagram showing pan-cancer TF regulons. Dot size represents the number of target genes for each TF, and edge width represents the number of shared target genes between regulons. **h,** Volcano plot showing relative activation and statistical significance of TF regulons across cancer cell lines. Strongly activated regulons exhibited high tissue specificity, including HAND2 and PHOX2B in neuroblastoma, AR in prostate cancer, HOXB4 in melanoma, and CEBPE in acute promyelocytic leukemia. **i,** Box plot showing the number of significantly activated TF regulons across GRN clusters 4–6, corresponding to epithelial (n cell line = 5), intermediate (n cell line = 10), and mesenchymal states (n cell line = 18). Multiple-test-corrected p-values from Tukey tests following one-way ANOVA are reported. **j,** Box plot showing the extent of significantly activated TF regulons across GRN clusters 4–6, corresponding to epithelial (n cell line = 5), intermediate (n cell line = 10), and mesenchymal states (n cell line = 18). The difference between each cell line's z-scored regulon score and the mean background z-scored regulon score was used as a proxy. Multiple-test-corrected p-values from Tukey tests following one-way ANOVA are reported. **k,** Workflow for establishing a validation model to predict GRN-based cancer cell-line phenotypes. **l,** Predicted GRN cluster identities of four overlapping cell lines (n = 4) between our dataset and Zhu et al.[1], showing assignment to the expected epithelial cluster 6 or mesenchymal cluster 8. **m,** Predicted GRN cluster identities of breast cancer cell lines (n = 12) from Zhu et al.[1], showing concordance between epithelial/stromal-EMT phenotypes and assignment to GRN cluster 6 or 8. Box plots show median, IQR, and 1.5× IQR whiskers.

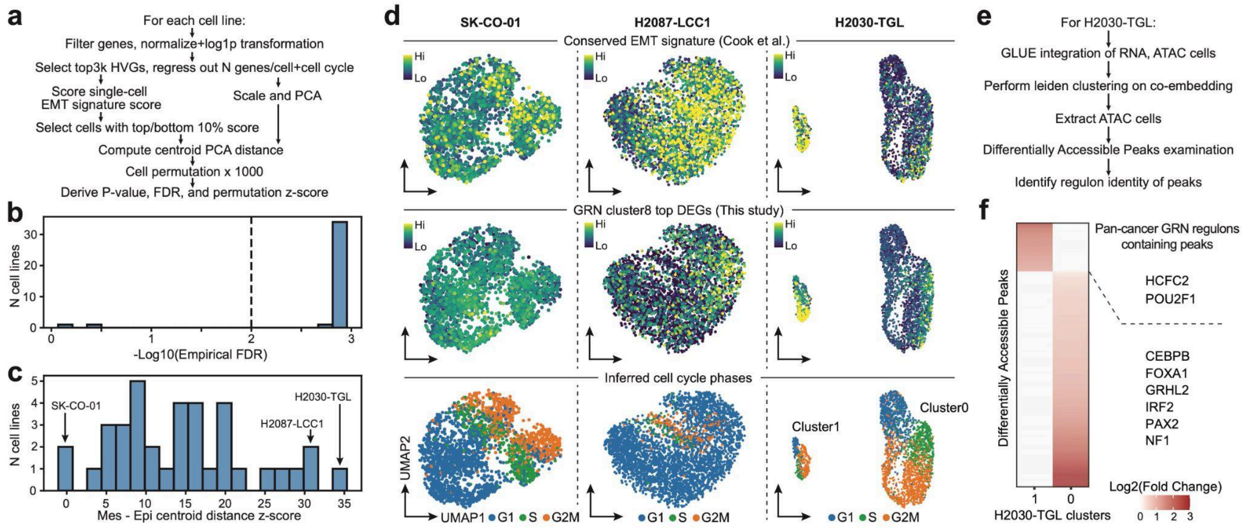

Supplementary Figure 4. EMT-associated intra-cell-line heterogeneity and regulatory validation.

a, Schematic of the workflow used to quantify EMT-associated heterogeneity within each cell line. **b,** Distribution of empirical FDR values across cell lines. **c,** Distribution of permutation-based z-scores for EMT-high versus EMT-low centroid distances, highlighting SK-CO-01, H2087-LCC1, and H2030-TGL as representative examples. **d,** UMAP visualization of representative cell lines colored by conserved EMT signature, GRN cluster 8 DEG signature, and inferred cell-cycle phase. Left panel: n cell line = 1, n cells = 3,601; middle panel: n cell line = 1, n cells = 3,652; right panel: n cell line = 1, n cells = 2,532. Score distributions for visualization were scaled separately within each cell line. **e,** Schematic of the workflow used to identify differentially accessible peaks and associated regulons between H2030-TGL intra-cell-line states. **f,** Heatmap of differentially accessible peaks in two H2030-TGL clusters (N upregulated peaks in cluster 0 = 1,321, n upregulated peaks in cluster 1 = 300; n cell line = 1), and associated pan-cancer GRN regulons containing these peaks.

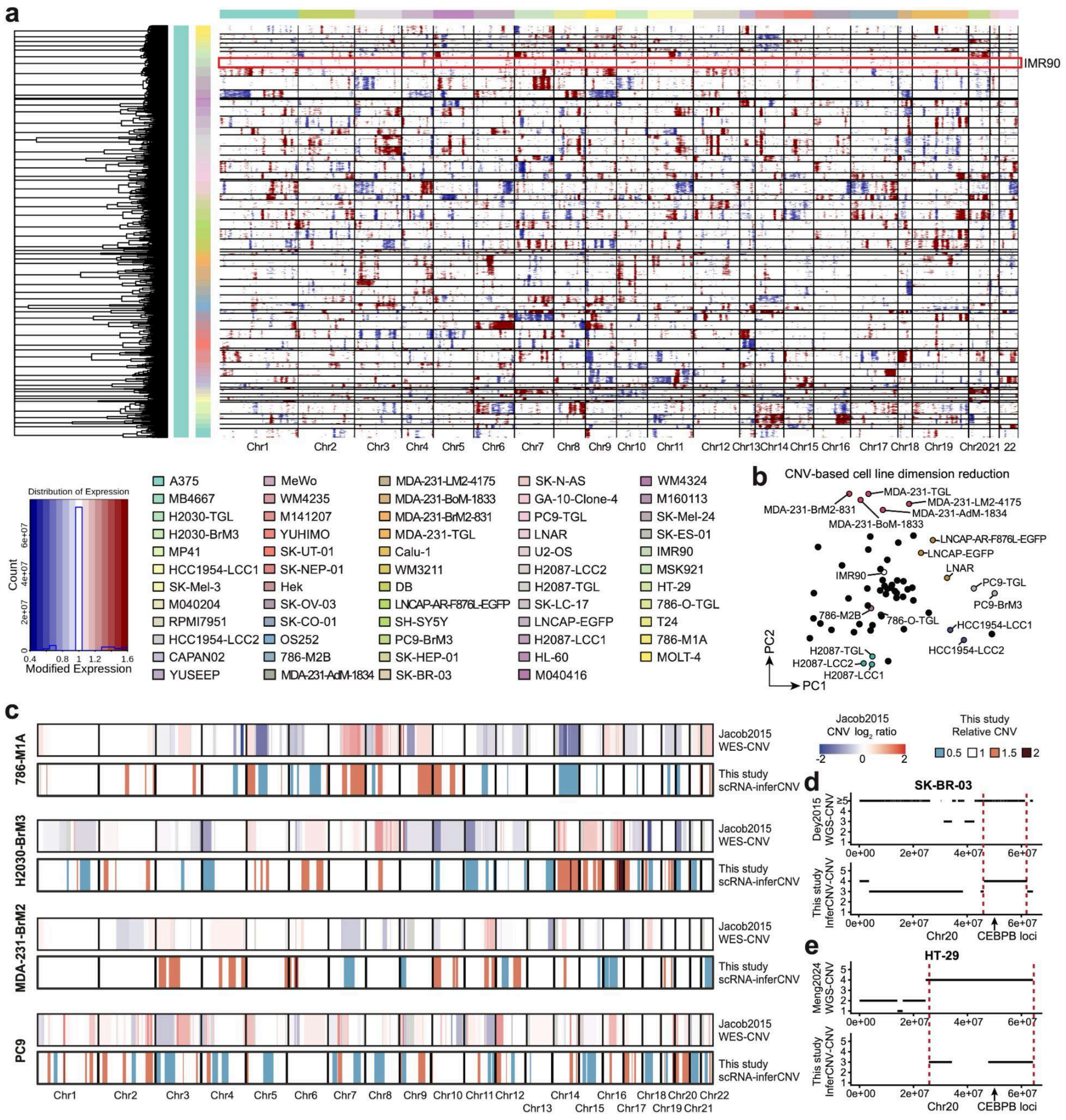

Supplementary Figure 5. Pan-cancer cell line copy number variation inference by InferCNV.

a, Heatmap showing smoothed RNA gene expression–based CNV patterns across meta-cells of pan-cancer cell lines (n = 59). The karyotypically normal diploid cell line IMR90 is highlighted. Hierarchical clustering of CNV patterns was performed within each cell line, and no statistically significant subclones were identified. **b,** PCA plot showing the distribution of cell lines (n cell lines = 59) along the first two principal components based on inferred gene-level CNV profiles. Cell lines with close genetic evolutionary proximity, as well as IMR90, are highlighted. **c,** Comparison of genome-wide CNV profiles across 22 autosomes inferred from scRNA-seq in this study with published WES-derived CNV profiles from Jacob et al.[2] for 786-M1A,

H2030-BrM3, MDA231-BrM2, and PC9. **d,** Comparison of chr20 amplifications in SK-BR-03 between published WGS-derived CNV data[3,4] and scRNA-seq-based inferCNV from this study. **e,** Comparison of chr20 CNV profiles in HT-29 between published WGS-derived CNV data and scRNA-seq-based inferCNV from this study.

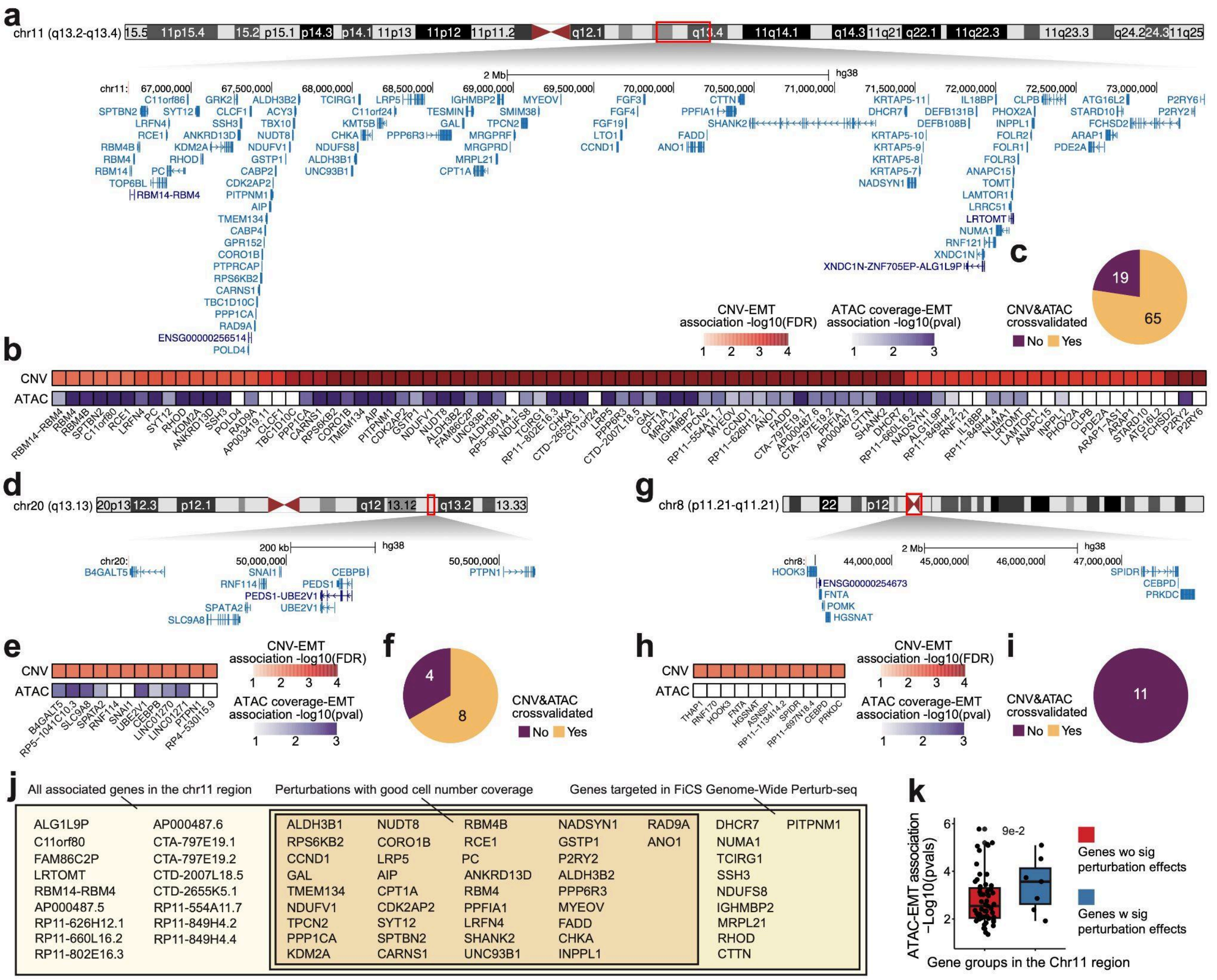

Supplementary Figure 6. Characterization of the association between CNV of hotspot regions and EMT.

a, Genome track displaying the gene distribution within the chromosome 11 hotspot region. **b,** Heatmaps showing the statistical significance of gene expression–derived CNV–EMT pseudotime associations (top) and normalized ATAC coverage-based gene-EMT pseudotime associations (bottom) within the chromosome 11 hotspot region (n genes = 84) across 33 cell lines. White tiles indicate non-significant associations (Pearson correlation p ≥ 0.05). **c,** Pie chart showing the number of genes within the chromosome 11 hotspot region significantly associated with CNV-EMT pseudotime, and the subset validated by ATAC-EMT association. **d,** Genome track displaying the gene distribution within the chromosome 20 hotspot region. **e,** Heatmaps showing the statistical significance of gene expression–derived CNV-EMT pseudotime associations (top) and normalized ATAC coverage-based gene-EMT pseudotime associations (bottom) within the chromosome 20 hotspot region (n genes = 12) across 33 cell lines. White tiles indicate non-significant associations (Pearson correlation p ≥ 0.05). **f,** Pie chart showing the number of genes within the chromosome 20 hotspot region significantly associated with CNV-EMT pseudotime, and the subset validated by ATAC-EMT association. **g,** Genome track displaying the gene distribution within the chromosome 8 region that failed ATAC validation. **h,** Heatmaps showing the statistical significance of gene expression–derived CNV–EMT

pseudotime associations (top) and normalized ATAC coverage–based gene–EMT pseudotime associations (bottom) within the chromosome 8 region (n genes = 11) across 33 cell lines. White tiles indicate non-significant associations (Pearson correlation $p \geq 0.05$); no gene in this region exhibited a significant ATAC-EMT association. **i,** Pie chart showing the number of genes within the chromosome 8 region significantly associated with CNV–EMT pseudotime, and the subset validated by ATAC–EMT association. **j,** Venn diagram showing genes within the chromosome 11 hotspot region targeted in the FiCS Perturb-seq dataset. Genes not covered were primarily noncoding, and 10 perturbations were excluded due to low cell number coverage ($n < 100$). **k,** Box plot comparing the statistical significance of ATAC signal–EMT associations between genes with or without significant phenotypic shifts in the Perturb-seq data (no-shift group: $n = 58$; significant-shift group: $n = 7$). Boxes in box plots indicate the median and interquartile range (IQR), with whiskers indicating 1.5× IQR.

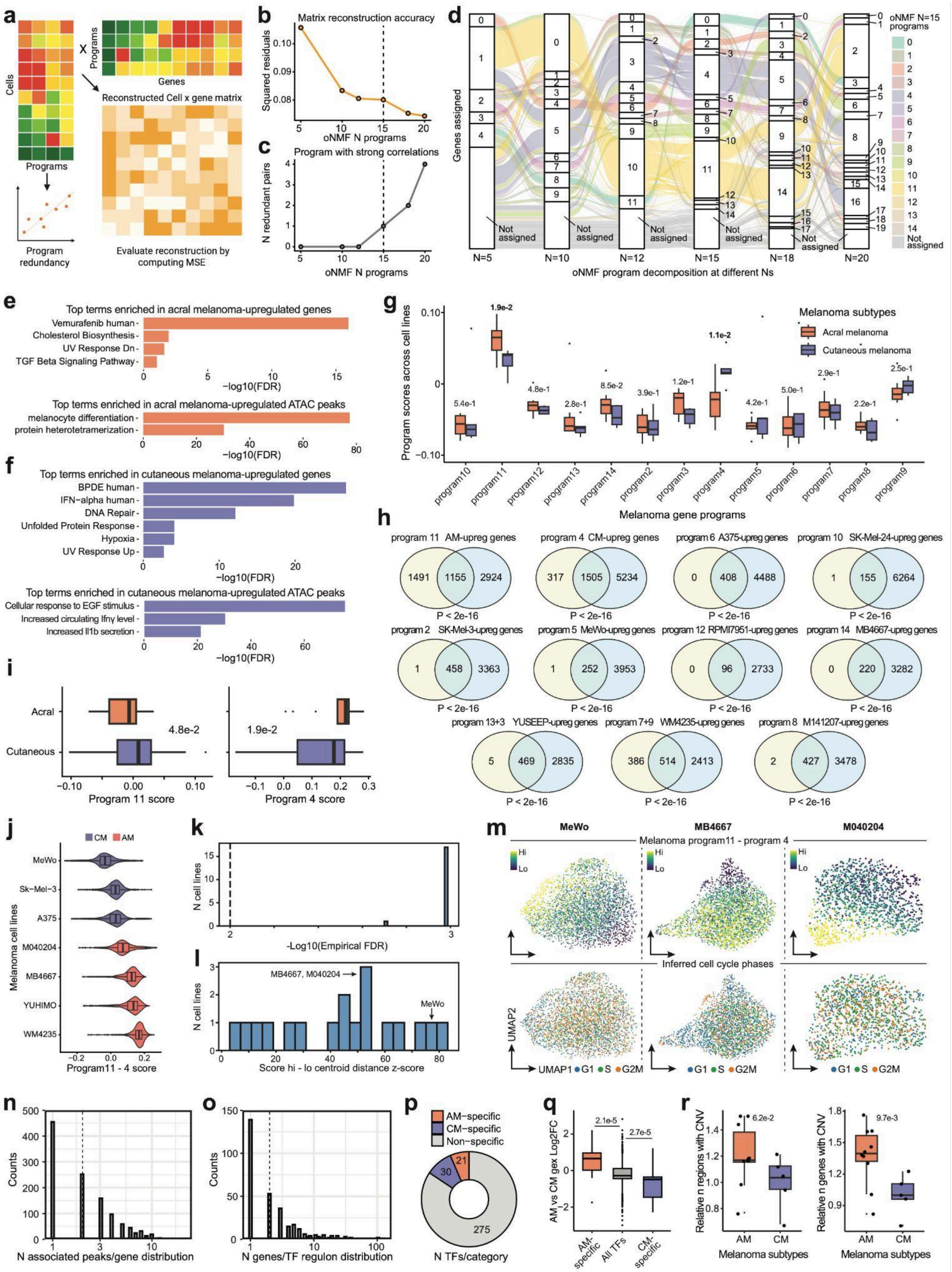
a
Cells
Programs
X
Genes
Reconstructed Cell x gene matrix
Program redundancy
Evaluate reconstruction by computing MSE
b
Matrix reconstruction accuracy
Squared residuals
oNMF N programs
c
Program with strong correlations
N redundant pairs
oNMF N programs
d
Genes assigned
Not assigned
N=5
N=10
N=12
N=15
N=18
N=20
oNMF program decomposition at different Ns
oNMF N=15 programs
e
Top terms enriched in acral melanoma-upregulated genes
Vemurafenib human
Cholesterol Biosynthesis
UV Response Dn
TGF Beta Signaling Pathway
-log10(FDR)
Top terms enriched in acral melanoma-upregulated ATAC peaks
melanocyte differentiation
protein heterotetramerization
-log10(FDR)
f
Top terms enriched in cutaneous melanoma-upregulated genes
BPDE human
IFN−alpha human
DNA Repair
Unfolded Protein Response
Hypoxia
UV Response Up
-log10(FDR)
Top terms enriched in cutaneous melanoma-upregulated ATAC peaks
Cellular response to EGF stimulus
Increased circulating Ifny level
Increased Il1b secretion
-log10(FDR)
g
Program scores across cell lines
Melanoma subtypes
Acral melanoma
Cutaneous melanoma
program10
program11
program12
program13
program14
program2
program3
program4
program5
program6
program7
program8
program9
Melanoma gene programs
h
program 11 AM-upreg genes
program 4 CM-upreg genes
program 6 A375-upreg genes
program 10 SK-Mel-24-upreg genes
program 2 SK-Mel-3-upreg genes
program 5 MeWo-upreg genes
program 12 RPMI7951-upreg genes
program 14 MB4667-upreg genes
program 13+3 YUSEEP-upreg genes
program 7+9 WM4235-upreg genes
program 8 M141207-upreg genes
P < 2e-16
i
Acral
Cutaneous
Program 11 score
Program 4 score
j
CM
AM
MeWo
Sk−Mel−3
A375
M040204
MB4667
YUHIMO
WM4235
Melanoma cell lines
Program11 - 4 score
k
N cell lines
-Log10(Empirical FDR)
l
MB4667, M040204
MeWo
N cell lines
Score hi - lo centroid distance z-score
m
MeWo
MB4667
M040204
Melanoma program11 - program 4
Hi
Lo
Inferred cell cycle phases
UMAP2
UMAP1
G1
S
G2M
n
Counts
N associated peaks/gene distribution
o
Counts
N genes/TF regulon distribution
p
AM−specific
CM−specific
Non−specific
N TFs/category
q
AM vs CM gex Log2FC
AM-specific
All TFs
CM-specific
r
Relative n regions with CNV
Relative n genes with CNV
AM
CM
Melanoma subtypes

Supplementary Figure 7. Characterization of Melanoma subtype-specific gene-regulatory features.

a, Schematic showing evaluation of oNMF decompositions. Schematic in **a** is created in BioRender. Xu, Z. (2026) https://BioRender.com/v1z1sik. **b,** Mean squared reconstruction residuals across oNMF program numbers; dashed line indicates selected N = 15. **c,** Number of redundant program pairs, defined by Pearson correlation > 0.65 between program scores across melanoma cells; dashed line indicates N = 15. **d,** Sankey diagram showing conservation of gene membership across decompositions, with genes colored by program identity in the selected N = 15 decomposition. **e, f,** Bar plots showing terms enriched among upregulated genes and ATAC peaks in AM (e) or CM (f). **g,** Box plot comparing pseudobulk gene-program scores between AM (n = 10) and CM (n = 5) cell lines. P-values were calculated using two-sided Wilcoxon tests. **h,** Venn diagrams showing overlaps between oNMF-derived programs and DESeq2-defined DEGs. Two-sided p-values are from Fisher's exact tests. **i,** Program 11 and Program 4 scores in acral (n = 42) versus cutaneous melanoma (n = 15) from an external validation cohort. **j,** Distribution of single-cell Program 11 minus Program 4 scores across example melanoma cell lines. N cells per group from top to bottom: 3,575, 2,877, 5,375, 1,353, 2,950, 1,260, 2,599. **k, l,** Distributions of empirical FDR (k) and permutation-based z-scores (l) across melanoma cell lines (n = 15); MB4667, M040204, and MeWo are highlighted. **m,** UMAPs of representative melanoma cell lines colored by Program 11 minus Program 4 score and cell-cycle phase, showing intra-cell-line variation not explained by cell cycle. Scores were scaled separately within each cell line for visualization. Left panel: n cell line = 1, n cells = 3,575; middle panel: n cell line = 1, n cells = 2,950, right panel: n cell line = 1, n cells = 1,353. **n, o,** Histograms showing ATAC peaks associated with downstream genes (n) and target genes per TF (o); dashed lines indicate medians of 2. **p,** Donut chart showing TF counts across melanoma regulatory categories. **q,** Box plot showing AM versus CM relative expression changes of AM-specific TFs (n = 21) and CM-specific TFs (n = 30), using all FigR-examined TFs as background (n = 1,141). Two-sided p-values from Tukey's tests following one-way ANOVA are reported. **r,** Box plots comparing CNV regions and CNV genes between AM and CM cell lines (AM, n = 10; CM, n = 5). Two-sided p-values from Wilcoxon tests are reported. Box plots show median, IQR, and 1.5× IQR whiskers.

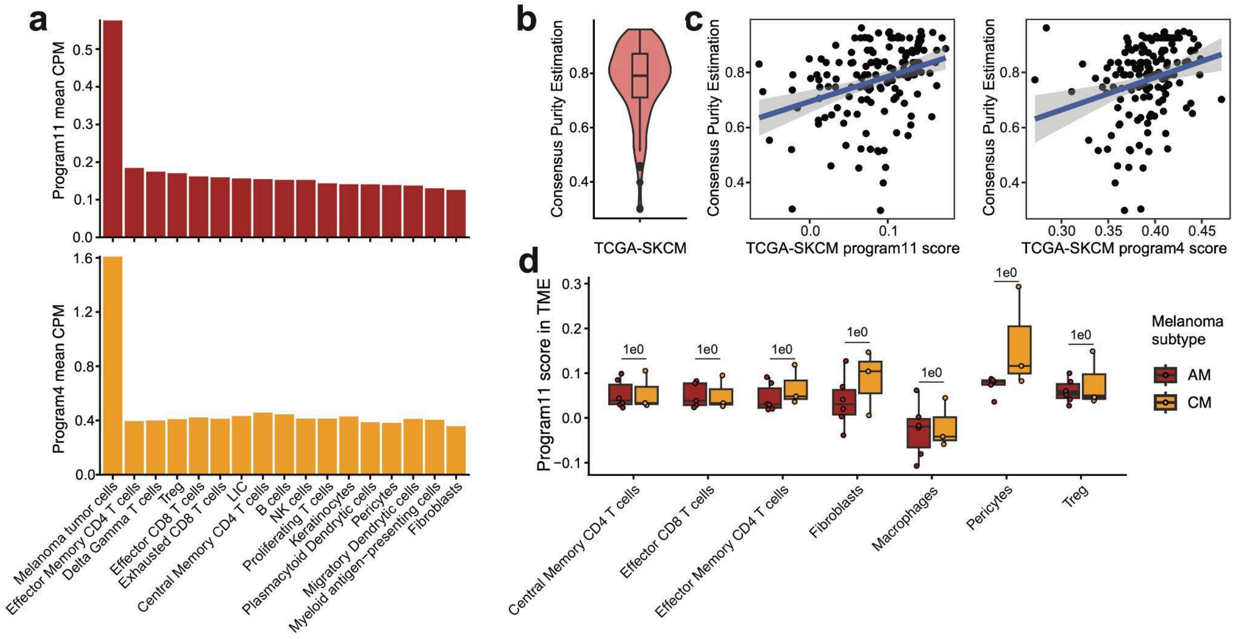

Supplementary Figure 8. Evaluation of tumor-cell specificity and TME spill-over for melanoma subtype-associated programs.

a, Mean CPM expression of program 11 and program 4 genes across melanoma tumor cells and non-malignant TME cell types in an external patient melanoma single-cell RNA-seq data. **b,** Distribution of consensus tumor purity estimates across TCGA-SKCM bulk RNA-seq samples. The shaded band represents the 95% confidence interval around the fitted line. **c,** Association between consensus tumor purity estimates and bulk program 11 or program 4 scores in TCGA-SKCM samples. **d,** Comparison of program 11 scores in non-malignant TME cell types between AM (n = 6) and CM (n = 3) patient single-cell RNA-seq samples.

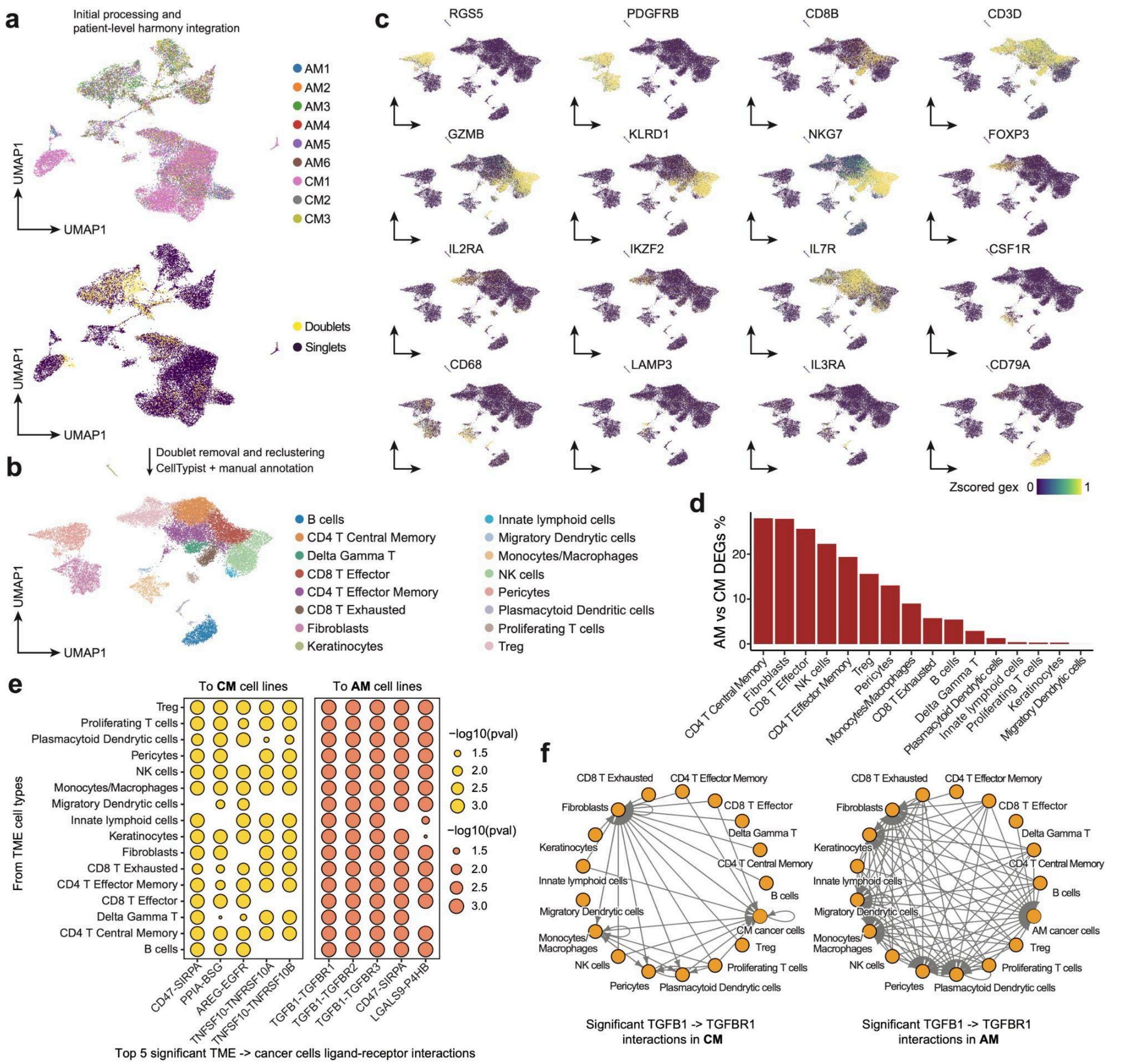

Supplementary Figure 9. Patients' TME single-cell RNA-seq data reprocessing and examinations of melanoma subtype-associated changes.

a, First-round patient-level Harmony-integrated UMAP of TME single-cell RNA-seq from AM and CM patients (N patient samples = 9, n cells = 24,293). Data was reprocessed from[5]. Cells are colored by patient identity (top) and computationally identified doublets (bottom). **b,** Final patient-level Harmony-integrated UMAP of TME single-cell RNA-seq from AM and CM patients (N patient samples = 9, n cells = 19,599). Cells are colored by cell-type labels annotated using CellTypist[6] and manual curation. **c,** UMAP colored by representative marker genes of major TME cell types (N patient samples = 9, n cells = 19,599). **d,** Bar plot showing the percentage of significantly differentially expressed genes for each TME cell type between AM and CM patients. **e,** Dot heatmaps showing the top five ligand–receptor pairs with the highest number of significant paired activations between CM (n patients = 3) or AM (n patients = 6) patients’ TME cell types (n = 16) (ligand genes) and CM (n cell lines = 5) or AM (n cell lines = 10) cell lines

(receptor genes), identified using CellPhoneDB[7]. Permutation-based p-values exported from CellPhoneDB are reported. **f,** Network diagrams showing all significant TGFB1–TGFBR1 paired activations across TME cell types and cancer cell lines in the CM (left) or AM (right) subtype, identified using CellPhoneDB.

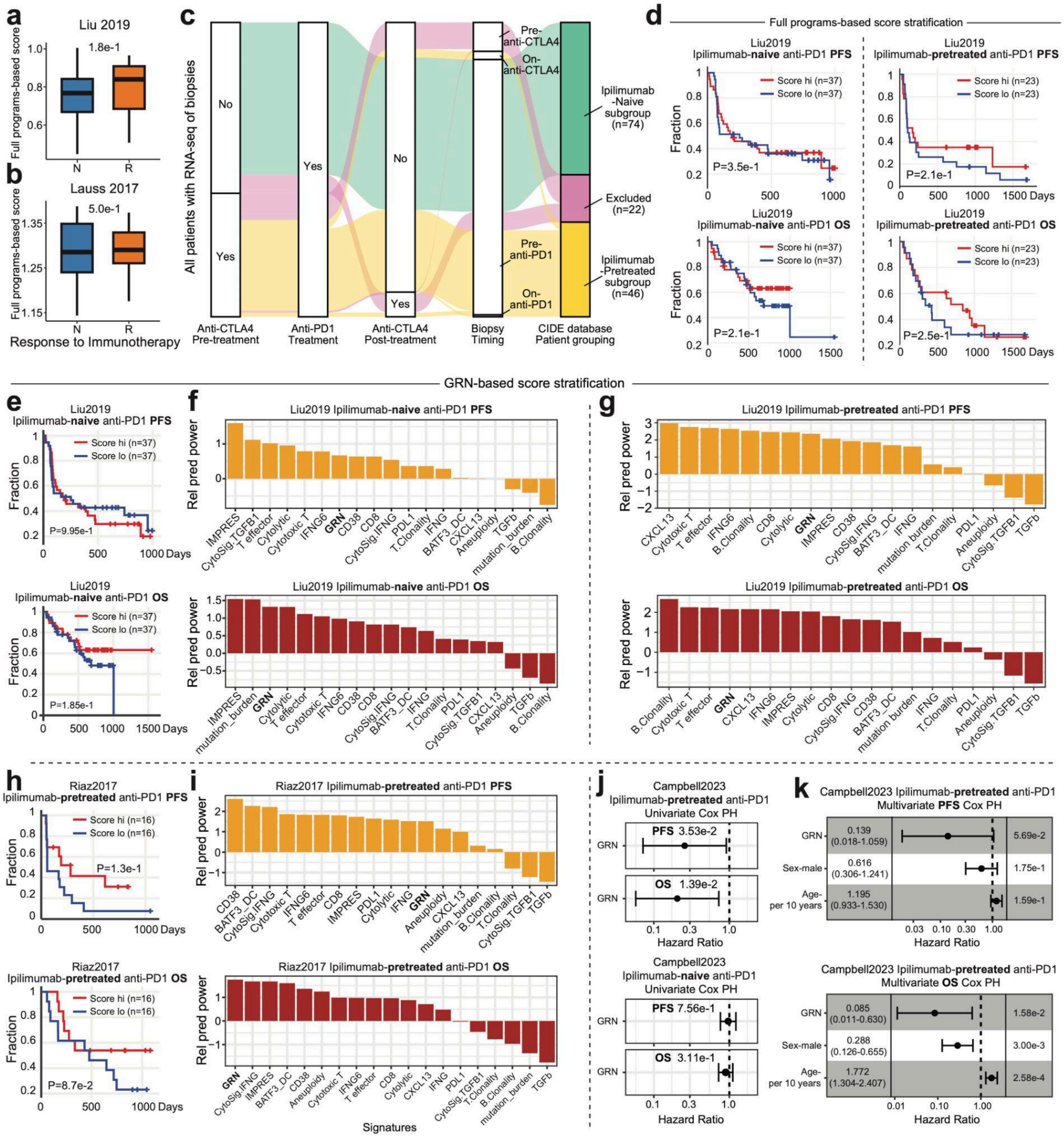

Supplementary Figure 10. Comparative analyses of the power of gene signatures in immunotherapy patient cohorts.

a, Box plot comparing scores of Ipilimumab-pretreated anti-PD-1 responder (n = 17) and non-responder (n = 29) from Liu2019[8], calculated as full-gene Program 4 minus full-gene Program 11. P-value, two-sided Wilcoxon test. **b,** Box plot comparing scores of adoptive TIL therapy responder (n = 10) and non-responder (n = 15) from[9], calculated as full-gene Program 4 minus full-gene Program. P-value, two-sided Wilcoxon test. **c,** Sankey diagram classifying Liu2019 anti-PD-1-treated melanoma patients by prior anti-CTLA-4 treatment, post-treatment anti-CTLA-4 exposure, and biopsy timing. **d,** Kaplan–Meier curves showing PFS and OS in Liu2019 anti-PD-1-treated patients stratified by ipilimumab-naive (left) or ipilimumab-pretreated

(right) status and full-program scores. In Ipilimumab-naive cohort: n hi-score patients = 37; n lo-score patients = 37. In Ipilimumab-pretreated cohort: n hi-score patients = 23; n lo-score patients = 23. P-value, two-sided Wald test for the signature term from univariate Cox regression. **e,** Kaplan–Meier curves showing PFS (top) and OS (bottom) in ipilimumab-naive, anti-PD-1-treated Liu2019 patients stratified by GRN scores. N hi-score patients = 37; n lo-score patients = 37. P-value, two-sided Wald test for the signature term from univariate Cox regression. **f,** Bar plots showing the relative predictive power of gene signatures for PFS (top) and OS (bottom) in ipilimumab-naive, anti-PD-1-treated Liu2019 patients. Coefficients from univariate Cox regression were z-scored. Established prognostic signatures were compiled by CIDE. **g,** Bar plots showing the relative predictive power of gene signatures for PFS (top) and OS (bottom) in ipilimumab-pretreated, anti-PD-1-treated Liu2019 patients. **h,** Kaplan–Meier curves showing PFS (top) and OS (bottom) in ipilimumab-pretreated, anti-PD-1-treated Riaz2017 patients stratified by GRN scores. N hi-score patients = 16; n lo-score patients = 16. P-value, two-sided Wald test for the signature term from univariate Cox regression. **i,** Bar plots showing the relative predictive power of gene signatures for PFS (top) and OS (bottom) in ipilimumab-pretreated, anti-PD-1-treated Riaz2017[10] patients (n = 32). **j,** Error-bar plots showing hazard ratios and 95% confidence intervals (CIs) for the GRN signature in predicting PFS (top) and OS (bottom) in integrated ipilimumab-pretreated, anti-PD-1-treated Campbell2023[11] cohorts. N patients in the Ipilimumab-pretreated anti-PD-1 integrated cohort = 102; n patients in the Ipilimumab-naive anti-PD-1 integrated cohort = 335. P-value, two-sided Wald test for the signature term from univariate Cox regression. **k,** Forest plots showing hazard ratios and 95% CIs for multiple terms in predicting PFS (top) and OS (bottom) in integrated ipilimumab-pretreated, anti-PD-1-treated Campbell2023 cohorts (n patients = 102). P-values, two-sided Wald tests from Cox regression. Box plots show median and interquartile range, with whiskers extending to 1.5× IQR.

Supplementary Reference

1. Zhu, Q. *et al.* Single cell multi-omics reveal intra-cell-line heterogeneity across human cancer cell lines. *Nat Commun* **14**, 8170 (2023).
2. Jacob, L. S. *et al.* Metastatic Competence Can Emerge with Selection of Preexisting Oncogenic Alleles without a Need of New Mutations. *Cancer Res* **75**, 3713–3719 (2015).
3. Dey, S. S., Kester, L., Spanjaard, B., Bienko, M. & van Oudenaarden, A. Integrated genome and transcriptome sequencing of the same cell. *Nat Biotechnol* **33**, 285–289 (2015).
4. Meng, X.-N. *et al.* Dynamic genomic changes in methotrexate-resistant human cancer cell lines beyond DHFR amplification suggest potential new targets for preventing drug resistance. *Br J Cancer* **130**, 1819–1827 (2024).
5. Zhang, C. *et al.* A single-cell analysis reveals tumor heterogeneity and immune environment of acral melanoma. *Nat Commun* **13**, 7250 (2022).
6. Domínguez Conde, C. *et al.* Cross-tissue immune cell analysis reveals tissue-specific features in humans. *Science* **376**, eabl5197 (2022).
7. Efremova, M., Vento-Tormo, M., Teichmann, S. A. & Vento-Tormo, R. CellPhoneDB: inferring cell-cell communication from combined expression of multi-subunit ligand-receptor complexes. *Nat Protoc* **15**, 1484–1506 (2020).
8. Liu, D. *et al.* Integrative molecular and clinical modeling of clinical outcomes to PD1 blockade in patients with metastatic melanoma. *Nat Med* **25**, 1916–1927 (2019).
9. Lauss, M. *et al.* Mutational and putative neoantigen load predict clinical benefit of adoptive T cell therapy in melanoma. *Nat Commun* **8**, 1738 (2017).
10. Riaz, N. *et al.* Tumor and Microenvironment Evolution during Immunotherapy with Nivolumab. *Cell* **171**, 934–949.e16 (2017).
11. Campbell, K. M. *et al.* Prior anti-CTLA-4 therapy impacts molecular characteristics associated with anti-PD-1 response in advanced melanoma. *Cancer Cell* **41**, 791–806.e4 (2023).
