# Supplementary material for: Single-cell multimodal profiling of pan-cancer cell lines uncovers gene regulatory principles underlying intrinsic cell states and environmental features
Source: Nat Commun. 2026 Jul 23;17:6975. doi: 10.1038/s41467-026-75360-7 (PMC13396447; doi:10.1038/s41467-026-75360-7)
Supplement: Supplementary file 2 — Description of Additional Supplementary Information [file 41467_2026_75360_MOESM2_ESM.pdf]

Title: Supplementary Data 1
Description: Information on cell lines profiled in this study. This table details the source, tissue of origin, cancer type, and culture conditions of each cancer cell line used.

Title: Supplementary Data 2
Description: Pan-cancer cell line RNA-based marker gene list. This table lists significantly upregulated marker genes for each cancer cell line profiled, identified using Scanpy. Multiple test-corrected FDR values of wilcox test are reported.

Title: Supplementary Data 3
Description: Pan-cancer cell line ATAC-based marker gene list. This table lists genes with significantly upregulated gene activity inferred from ATAC-seq data for each cancer cell line profiled. Multiple test-corrected FDR values of wilcox test are reported.

Title: Supplementary Data 4
Description: Pan-cancer gene regulatory network. This table details TF-ATAC peak-target gene triplets identified at the pan-cancer level using SCENIC+.

Title: Supplementary Data 5
Description: Cancer cell line regulon scores. This table provides the AUCell scores of each pan-cancer regulon across all profiled cell lines.

Title: Supplementary Data 6
Description: Regulon score-based cell line clustering information. This table provides the hierarchical clustering assignments of pan-cancer cell lines based on the activity profiles of TF regulons.

Title: Supplementary Data 7
Description: Statistical examination of cancer cell line-specific regulon activation. This table lists regulons whose activity scores were significantly higher than background in individual cancer cell lines. Multiple test-corrected FDR values of wilcox test p-values are reported.

Title: Supplementary Data 8
Description: Gene expression-derived gene-level copy number variation of cancer cell lines. This table provides CNV profiles of cancer cell lines inferred using InferCNV.

Title: Supplementary Data 9
Description: Gene CNV-EMT association analysis. This table details the statistical significance of associations between gene-level CNVs within identified hotspot regions and EMT pseudotime across RNA and ATAC modalities. Multiple-test corrected two-sided pearson correlation p-values are reported for the RNA-EMT association. Uncorrected two-sided pearson correlation p-values are reported for the ATAC-EMT association as the validation.

Title: Supplementary Data 10

Description: Melanoma gene program members. This table lists member genes of 15 gene programs deconvolved from the melanoma cancer cell line cohort using oNMF implemented in DSPIN.

Title: Supplementary Data 11
Description: DEG-refined melanoma gene programs 4 and 11. This table lists member genes of the CM universal gene program (4) and the AM universal gene program (11) after intersection with significantly differentially expressed genes identified by DESeq2.

Title: Supplementary Data 12
Description: Melanoma subtype-specific gene regulatory network. This table lists TFs identified as melanoma subtype-specific and their downstream target genes within either the AM or CM universal gene programs. Uncorrected permutation-calibrated nominal p-values exported from FigR are reported.

Title: Supplementary Data 13
Description: Significantly associated peaks within melanoma subtype-specific gene regulatory networks. This table lists ATAC peaks associated with melanoma subtype-specific TFs and their downstream genes within the AM or CM universal gene programs. Uncorrected permutation-calibrated nominal p-values exported from FigR are reported.

Title: Supplementary Data 14
Description: Association between the DEG-refined AM universal gene program and computational deconvolution of immune infiltration in the TCGA SKCM cohort. This table provides correlation coefficients and statistical significance values for these associations. Multiple-test corrected FDR of two-sided pearson correlation p-values are reported.

Title: Supplementary Data 15
Description: Differentially expressed genes of single-cell RNA-seq-profiled TME cell types between AM and CM patients. This table lists significantly differentially expressed genes between AM and CM patients across TME cell types, identified using Seurat. Multiple test-corrected FDR values of wilcox test p-values are reported.

Title: Supplementary Data 16
Description: Significantly differentially expressed ligand and receptor genes in melanoma gene programs. This table lists ligand and receptor genes that are significantly differentially expressed and belong to oNMF-deconvolved melanoma gene programs.
