# Supplementary material for: Single-cell multimodal profiling of pan-cancer cell lines uncovers gene regulatory principles underlying intrinsic cell states and environmental features
Source: Nat Commun. 2026 Jul 23;17:6975. doi: 10.1038/s41467-026-75360-7 (PMC13396447; doi:10.1038/s41467-026-75360-7)
Supplement: Supplementary file 4 — Reporting Summary [file 41467_2026_75360_MOESM4_ESM.pdf]

nature portfolio

Corresponding author(s): Wei Zhou, Junyue Cao

Last updated by author(s): Jun 25, 2026

Reporting Summary

Nature Portfolio wishes to improve the reproducibility of the work that we publish. This form provides structure for consistency and transparency in reporting. For further information on Nature Portfolio policies, see our Editorial Policies and the Editorial Policy Checklist.

Statistics

For all statistical analyses, confirm that the following items are present in the figure legend, table legend, main text, or Methods section.

n/a	Confirmed	
☐	☒	The exact sample size (n) for each experimental group/condition, given as a discrete number and unit of measurement
☐	☒	A statement on whether measurements were taken from distinct samples or whether the same sample was measured repeatedly
☐	☒	The statistical test(s) used AND whether they are one- or two-sided<br>*Only common tests should be described solely by name; describe more complex techniques in the Methods section.*
☐	☒	A description of all covariates tested
☐	☒	A description of any assumptions or corrections, such as tests of normality and adjustment for multiple comparisons
☐	☒	A full description of the statistical parameters including central tendency (e.g. means) or other basic estimates (e.g. regression coefficient) AND variation (e.g. standard deviation) or associated estimates of uncertainty (e.g. confidence intervals)
☐	☒	For null hypothesis testing, the test statistic (e.g. F, t, r) with confidence intervals, effect sizes, degrees of freedom and P value noted<br>*Give P values as exact values whenever suitable.*
☒	☐	For Bayesian analysis, information on the choice of priors and Markov chain Monte Carlo settings
☐	☒	For hierarchical and complex designs, identification of the appropriate level for tests and full reporting of outcomes
☐	☒	Estimates of effect sizes (e.g. Cohen's d, Pearson's r), indicating how they were calculated

Our web collection on statistics for biologists contains articles on many of the points above.

Software and code

Policy information about availability of computer code

Data collection	No software was used except for Illumina RTA basecalling.
Data analysis	Common, freely available sequencing data analysis software was used to analyze data, as described in Methods: bcl2fastq/v2.19.0.316, python/v2.7.13 (for fastq reads preprocessing) and python/v3.8 (for downstream analyses), trim_galore/v0.6.7, STAR/v 2.7.9a, featureCounts/2.0.1, bedtools/2.30.0, Picard/v2.27.4, MACS2/2.2.9.1, MACS3/3.0.0b3, SnapATAC2/2.6.1, deepTools/3.5.1, R/4.1.1, samtools/1.13, bowtie2/2.3.0, cutadapt/3.4, eulerr/6.1.1, scGLUE/0.4.1, rhdf5/2.38.1, Seurat/4.2.0, clusterProfiler/4.2.2, DESeq2/1.50.2, dspin/1.5.2, decoupler/2.1.4, inferCNV/3.20, GenomicRanges/1.46.1, GenomeInfoDb/1.30.0, IRanges/2.28.0, Matrix/1.4-1, dplyr/1.0.1, FigR/1.0.1, motifmatchr/1.28.0, ChromVAR/1.28.0, CellPhoneDB/5.0.0, SCENIC+/1.0a1, SEACells/0.3.3.

For manuscripts utilizing custom algorithms or software that are central to the research but not yet described in published literature, software must be made available to editors and reviewers. We strongly encourage code deposition in a community repository (e.g. GitHub). See the Nature Portfolio guidelines for submitting code & software for further information.

Data

Policy information about availability of data

All manuscripts must include a data availability statement. This statement should provide the following information, where applicable:

- Accession codes, unique identifiers, or web links for publicly available datasets
- A description of any restrictions on data availability
- For clinical datasets or third party data, please ensure that the statement adheres to our policy

The single-cell RNA and ATAC raw FASTQ data generated in this study have been deposited in the NCBI BioProject database under accession code PRJNA1354039 [https://www.ncbi.nlm.nih.gov/bioproject/?term=PRJNA1354039]. Processed gene count matrices, ATAC peak count matrices, and cell metadata generated in this study have been deposited in the NCBI Gene Expression Omnibus (GEO) database under accession code GSE311521 [https://www.ncbi.nlm.nih.gov/geo/query/acc.cgi?acc=GSE311521]. Public reference lung and breast tissue single-cell RNA-seq atlases data were retrieved via CZ Cell x Gene portal [https://cellxgene.cziscience.com/]. Public CNV and drug sensitivity data for cell lines were obtained from the Cancer Cell Line Encyclopedia [https://sites.broadinstitute.org/ccle/]. Public ChIP-seq data were obtained from ENCODE [https://www.encodeproject.org/]. The TF-ome CRISPRa Perturb-seq on RPE-1 cell line was obtained from Zenodo [https://zenodo.org/records/15213619]53. Public immunotherapy patient cohort data were collected by CIDE [https://cide.ccr.cancer.gov/]85. The conserved EMT signatures used in this study are available in the manuscript [https://www.nature.com/articles/s41467-020-16066-2]35. The FiCS HEK293T Genome-wide Perturb-seq data used in this study is available in the FigShare database [https://plus.figshare.com/articles/dataset/Processed_data_for_X-Atlas_Orion_Genome-wide_Perturb-seq_Datasets_via_a_Scalable_Fix-Cryopreserve_Platform_for_Training_Dose-Dependent_Biological_Foundation_Models/29190726]54. Subtype-resolution patients' melanoma single-cell RNA-seq used in this study are available in the GEO database under accession code GSE215121 [https://www.ncbi.nlm.nih.gov/geo/query/acc.cgi?acc=GSE215121]59. The bulk RNA-seq of a SKCM cohort used in this study are available in the TCGA database [https://portal.gdc.cancer.gov/]127. Source data are provided with this paper, the remaining data are available within the Article, Supplementary Information or Source Data file.

Research involving human participants, their data, or biological material

Policy information about studies with human participants or human data. See also policy information about sex, gender (identity/presentation), and sexual orientation and race, ethnicity and racism.

Reporting on sex and gender	NA
Reporting on race, ethnicity, or other socially relevant groupings	NA
Population characteristics	NA
Recruitment	NA
Ethics oversight	NA

Note that full information on the approval of the study protocol must also be provided in the manuscript.

Field-specific reporting

Please select the one below that is the best fit for your research. If you are not sure, read the appropriate sections before making your selection.

☒ Life sciences ☐ Behavioural & social sciences ☐ Ecological, evolutionary & environmental sciences

For a reference copy of the document with all sections, see nature.com/documents/nr-reporting-summary-flat.pdf

Life sciences study design

All studies must disclose on these points even when the disclosure is negative.

Sample size	The study profiled 60 human cancer cell lines representing 16 tissue origins and 20 cancer types, yielding 240,957 single-nucleus RNA-seq profiles and 223,347 single-nucleus ATAC-seq profiles after quality control. Median nuclei per cell line were 3,784 (RNA) and 3,768 (ATAC). No statistical methods were used to predetermine sample size.
Data exclusions	No data was excluded.
Replication	The profiling experiment was conducted in 6 batches.
Randomization	Cancer cell lines were selected to represent diverse tissue origins and cancer types. No randomization was performed, as experiments involved profiling established cell lines without allocation to intervention groups. Nuclei from each cell line were processed independently for single-cell RNA-seq and ATAC-seq using combinatorial indexing, and computational analyses were performed uniformly across all samples.
Blinding	Blinding was not applicable to this study. Experiments involved profiling established cancer cell lines without subjective outcome assessment. Computational analyses were performed using objective statistical criteria. For external patient cohort analyses, group annotations were based on publicly available metadata.

Reporting for specific materials, systems and methods

We require information from authors about some types of materials, experimental systems and methods used in many studies. Here, indicate whether each material, system or method listed is relevant to your study. If you are not sure if a list item applies to your research, read the appropriate section before selecting a response.

Materials & experimental systems

n/a	Involved in the study
☒	☐ Antibodies
☐	☒ Eukaryotic cell lines
☒	☐ Palaeontology and archaeology
☒	☐ Animals and other organisms
☒	☐ Clinical data
☒	☐ Dual use research of concern
☒	☐ Plants

Methods

n/a	Involved in the study
☒	☐ ChIP-seq
☒	☐ Flow cytometry
☒	☐ MRI-based neuroimaging

Eukaryotic cell lines

Policy information about cell lines and Sex and Gender in Research

Cell line source(s)	For the acral melanoma cell lines: YUHIMO, YUSEEP, and YUSUSA were obtained from Yale University. M040204, M040416, M141207, and M160113 were obtained from the University of Zurich. WM3211, WM4325, and WM4324 were obtained from the Wistar Institute. MB4667 was obtained from the University of Colorado. The remaining cell lines (HCC1954-LCC1, HCC1954-LCC2, MDA-231-BrM2-831, MDA-231-LM2-4175, MDA-231-AdM-1834, MDA-231-BoM-1833, MDA-231-TGL, SK-BR-03, H2030-BrM3, H2030-TGL, H2087-TGL, H2087-LCC1, H2087-LCC2, PC9-BrM3, PC9-TGL, Calu-1, SK-LC-17, SH-SY5Y, SK-N-AS, 786-M1A, 786-M2B, 768-O-TGL, CAPAN02, LNAR, LNCAP-EGFP, LNCAP-EGFP-c.2-F876L, HT-29, SK-CO-01, SK-OV-03, SK-UT-01, SK-HEP-01, MSK921, OS252, SK-ES-01, and SK-NEP-01 cell lines) were obtained from the Antibody and Bioresource Core Facility at Memorial Sloan Kettering Cancer Center. IMR90, MeWo, RPMI7951, Sk-Mel-24, Sk-Mel-3, DB, GA-10-Clone-4, MOLT-4, HL-60, U2-OS, and MP41 cell lines were obtained from the American Type Culture Collection.
Authentication	Cell lines were authenticated by checking the morphology and mapping sequencing reads from different cell lines to species-specific reference genomes.
Mycoplasma contamination	All cell lines used in this study were routinely tested for mycoplasma contamination using a PCR-based assay (Universal Mycoplasma Detection Kit, 30-1012K, ATCC) and were mycoplasma-negative.
Commonly misidentified lines (See ICLAC register)	No commonly misidentified cell lines were used.

Plants

Seed stocks	NA
Novel plant genotypes	NA
Authentication	NA
