# Supplementary material for: Single-cell multimodal profiling of pan-cancer cell lines uncovers gene regulatory principles underlying intrinsic cell states and environmental features
Source: Nat Commun. 2026 Jul 23;17:6975. doi: 10.1038/s41467-026-75360-7 (PMC13396447; doi:10.1038/s41467-026-75360-7)
Supplement: Supplementary file 5 — Transparent Peer Review file [file 41467_2026_75360_MOESM5_ESM.pdf]

nature portfolio

Peer Review File

Single-cell multimodal profiling of pan-cancer cell lines uncovers gene regulatory principles underlying intrinsic cell states and environmental features

Corresponding Author: Dr Junyue Cao

Parts of this Peer Review File have been redacted as indicated to remove third-party material.

This file contains all reviewer reports in order by version, followed by all author rebuttals in order by version.

Version 0:

Reviewer comments:

Reviewer #1

(Remarks to the Author)
Summary
Xu and colleagues present a single-cell multi-omic atlas of 60 human cancer cell lines spanning 20 cancer types, profiled using their EasySci platform for parallel RNA-seq and ATAC-seq. Through integrative analysis, the authors construct pan-cancer gene regulatory networks, identify conserved EMT-associated states across lineages, link copy number variations to cell state transitions. They further demonstrate that these in vitro-derived signatures correlate with tumor microenvironment composition and immunotherapy responses in patient cohorts.

General Remarks
This manuscript should be accepted with revisions. The integration of matched transcriptomic and epigenetic profiles across such a diverse panel of cell lines enables novel insights into regulatory mechanisms that would be difficult to obtain from primary tumor data alone and from one cancer type. The validation of in vitro findings using external perturbation datasets (CRISPRa/i screens, ENCODE ChIP-seq) and patient cohorts (TCGA, immunotherapy trials) strengthens the conclusions. However, the manuscript's current presentation poses challenges for readability and accessibility, particularly for readers not deeply versed in computational single-cell methods.

Several key methodological clarifications are needed. First, the authors should more explicitly justify why single-cell resolution was essential for their findings, given that many analyses present averaged expression across meta-cells or cell lines. For example, single-cell data revealed that even within established lines, cells occupy distinct states along the EMT continuum and enabled deconvolution of mixed phenotypic states within individual melanoma lines. Second, the CNV analysis relies on transcriptome-inferred copy number variations (using InferCNV) without validation from orthogonal DNA-based methods such as bulk whole-genome sequencing or array data from the Cancer Cell Line Encyclopedia. These clarifications would strengthen confidence in the CNV-EMT associations and help readers understand the specific advantages of the single-cell approach.

The following recommendations aim to enhance clarity and impact for a broader audience.

Major/Minor Points
Abstract
1. The abstract's transition from pan-cancer findings to the focused melanoma analysis feels abrupt. The authors state they performed a "focused analysis of acral and cutaneous melanoma" without explaining why these subtypes warranted special attention. The current framing misses an opportunity to highlight the significance of including rare cancer subtypes in large-scale atlases.

Introduction
1. The EasySci platform is central to the experimental approach, enabling parallel single-cell RNA and ATAC profiling across many samples. However, the Introduction does not introduce this technology or explain why it is particularly

beneficial for pan-cancer analysis. Readers unfamiliar with the method are left wondering how the authors achieved such scale.

Results
1. The Results section mentions EasySci only in passing (Figure 1a legend) without explaining its significance or advantages. For readers unfamiliar with single-cell methods, the technical achievement here may be underappreciated.
2. Figure 1c-d is visually overwhelming. The current presentation uses 60+ colors for individual cell lines, making it nearly impossible to identify which cell lines cluster together or discern tissue-of-origin patterns. The reader cannot meaningfully interpret these figures.
3. The concept of "meta-cells" (aggregating neighboring cells for multimodal analysis) is introduced without sufficient explanation for non-specialist readers. The rationale for why this aggregation is necessary for GRN construction and how it preserves biological variation while reducing sparsity is not clearly stated.
4. The description of TF regulon identification and clustering (Figure 2b-c) is dense. The text states that 92 activating TF regulons were used for hierarchical clustering, but it is unclear how these 92 were selected from the larger network and what "activating" means in this context.
5. Make sure you are extremely clear in the figures which data analysis are what you performed versus what are the validation sets.
6. Figure 2d shows mapping of lung and breast cancer cell lines to normal reference cell types. The text states that cells were "mapped," but the method is not clearly explained in the Results. The concept of projecting cancer cells onto normal developmental trajectories is powerful but requires clearer exposition.
7. The use of cytokine-treated cell line panels as a reference for EMT signature genes is introduced without justification. Readers may question why signatures from experimentally induced EMT are appropriate for analyzing basal heterogeneity across cancer cell lines.
8. The relationship between "GRN-based clustering" and "RNA-based clustering" (Figure 2h) is mentioned but not clearly distinguished. It is unclear whether these are independent classifications or whether one informed the other.
9. The term "genomic hotspot loci" is introduced (Figure 3c) but not defined. Are these known cancer-associated amplicons, or are they novel regions identified in this study? The distinction between expected findings and novel discoveries is unclear.
10. Figure 3d shows CNV patterns across epithelial, intermediate, and mesenchymal states, but the visual presentation makes it difficult to see the pattern being described—that amplifications are present in epithelial states but absent in mesenchymal states. The heatmap colors and organization do not clearly convey this trend.
11. The in silico knockout and Perturb-seq validation sections (Figure 3h-p) are dense and contain multiple interconnected analyses. The current flow is difficult to follow, especially for readers not familiar with perturbation screening approaches.
12. Figure 4b is difficult to interpret. The scatter plots show associations between oNMF-derived programs and established melanoma signatures, but the relationship between the two axes, what the error bars represent, and how to interpret the coloring are not immediately clear.
13. Figures 4g-k would benefit from consistent color schemes to help readers track which elements correspond to AM versus CM across panels. Currently, each panel uses independent coloring, making it difficult to follow the regulatory logic being described.
14. The transition from in vitro cell line findings to patient TME and immunotherapy response is conceptually important but presented as a series of correlative analyses without explicit articulation of the hypotheses being tested.
15. The description of immune signature scoring in Figure 5d relies on broad, general terms ("reduced translation," "increased heat shock responses") that do not clearly connect to specific immune functions or anti-tumor mechanisms. The current phrasing assumes prior knowledge of how these signatures relate to immune function, which may not hold.

Discussion
1. While the Discussion is comprehensive, several limitations and contextual considerations should be more explicitly addressed to provide a balanced interpretation
a. The study profiled 60 cell lines across 20 cancer types, which is substantial, but the Discussion does not acknowledge which cancer types were excluded or under-represented. Readers may wonder about the generalizability of findings to cancers not included in the panel (e.g., pancreatic, gastric, bladder carcinomas).
b. The identification of 15 orthogonal gene programs from melanoma cell lines using oNMF is presented as a key step in defining AM- and CM-specific programs. However, the Discussion does not address how sensitive these programs might be to the number of programs chosen (k) or whether alternative program numbers would yield different subtype-specific signatures. This is particularly relevant because these programs later form the basis for TME correlations and immunotherapy predictions.
c. The study exclusively uses cancer cell lines cultured in isolation, which by definition lack the complex tumor microenvironment, including immune cells, fibroblasts, endothelial cells, and stromal components, that influences cancer cell behavior in vivo

Reviewer #2

(Remarks to the Author)
I co-reviewed this manuscript with one of the reviewers who provided the listed reports. This is part of the Nature Communications initiative to facilitate training in peer review and to provide appropriate recognition for Early Career Researchers who co-review manuscripts.

Reviewer #3

(Remarks to the Author)
In “Single-cell multimodal profiling of pan-cancer cell lines uncovers gene regulatory principles underlying intrinsic cell states and environmental features,” the authors leveraged a newly generated, comprehensive cancer-cell-line atlas to dissect gene-regulatory networks and convergent molecular programs across tumor types. By integrating single-cell chromatin accessibility with matched transcriptomes, they identify gene-regulatory phenotypes that arise from differential activation of transcription-factors. At the pan-cancer level, analysis of cis-regulatory elements and upstream TF motifs reveals an EMT-linked cell state that recurs across lineages, supporting a universal transcriptional dedifferentiation program in epithelial cancers. Among the TFs implicated, CEBPB stands out as a driver of the epithelial phenotype, frequently amplified by copy-number gains.

Focusing on melanoma cell lines, the authors resolve subtype-specific transcriptional modules that separate cutaneous from acral melanoma. The most striking differentiating module was found to be JAK-STAT signaling, which is significantly elevated in cutaneous melanoma.

Crucially, the authors project the single-cell-derived regulatory programs onto bulk RNA-seq datasets from patient tumors. The acral-specific program was found to correlate with an immunosuppressive tumor microenvironment, whereas the cutaneous program aligns with a more active antitumor immune response. In addition, scoring subtype-specific programs in bulk tumors was capable of stratifies patients by response to immune checkpoint blockade, underscoring the translational relevance of the in-vitro cell-line derive transcriptional modules.

Overall, I consider this paper a valuable contribution. By integrating ATAC-seq, single-cell transcriptomics, and bulk RNA-seq, the authors present a thorough and well-grounded analysis. The creation of the EasySci cell-line resource will be a useful asset for the community. Nevertheless, I have identified several points that warrant further clarification regarding the novelty of certain results. I also provide suggestions to help strengthen and clarify the work.

Major comments:

1) Validation of the eight gene-regulatory clusters in independent datasets, such as the cell line cohort generated by Zhu Q et al. Nat Commun, 2023 (PMID: 38071219), will be essential to demonstrate reproducibility across different library-preparation techniques and sequencing platforms. Moreover, it will help highlight the novel insights of this work.

2) The manuscript lists TYR and PAX3 for skin melanoma, ASPH and CAPN2 for lung carcinoma, STARD3 for breast cancer, RHEB for prostate cancer, and TLE4 for neuroblastoma. However, several of listed genes are not strong, exclusive tissue-specific markers on their own. The expression of some of these genes is either relatively broad or context-dependent, and they are not routinely used as “representative marker genes” for the indicated tissues.

3) The authors write: “Conversely, GRHL2, CEBPB, and FOXC1 emerged as putative EMT suppressors: their expression and activity peaked in the epithelial state, and their regulons were negatively associated with EMT progression, extending previous observations from specific cancer types.” The statement should be refined for accuracy and to provide clearer contextualization of the results’ novelty. For example, GRHL2 has already been reported to inhibit EMT in breast cancer (PMID 22379025) and lung cancer (PMID 37600329); these two cancer types are the ones that predominantly populate clusters 6-8 in our analysis. More generally, the Discussion should more clearly delineate which aspects of the study are novel versus extensions of, and support for, existing literature.

4) In the absence of a dedicated normal-cell reference, inferCNV uses the average expression across all cells as a pseudo-reference. In cell-line datasets, where most cells share similar CNV profiles, the assumption regarding the existence of a substantial “baseline” population as a reference is often violated. The authors should therefore provide more detail in the appropriate Methods section on how the CNV analysis for the cell-line data was performed, including how statistically significant CNVs were determined.

5) Scoring the AM and CS signatures on bulk RNA-seq is appropriate, but the results must be interpreted in the context of tumor-cell specificity. The programs’ scores could be partially driven by changes in stromal or immune compartment composition rather than by the malignant cells alone. Therefore, the authors should provide evidence that the observed associations between the AM/CM programs and the various cell-type abundances are not merely due to gene expression spill-over from non-cancer fractions.

6) On a similar note, the TCGA-SKCM cohort is dominated by classic sun-exposed cutaneous melanomas; acral melanomas, if present at all, make up only a very small minority (SKCM = Skin Cutaneous Melanoma). Consequently, it is unclear what the AM score captures when applied to this dataset. In Fig 4d the authors present another bulk-RNA-seq cohort but only signaling activities that show significant subtype differences are displayed. To assess the specificity of the entire programs and to confirm the robustness of the scoring strategy, the manuscript should show the distribution of both the AM and CM scores across the relevant melanoma subtypes (i.e., cutaneous vs. acral). This will clarify how the scores behave in patients’ tumors of different origins, as one might expect the AM score to be elevated in acral melanomas and the CM score to be higher in cutaneous melanomas.

7) Regarding the immunotherapy-cohort analyses (i) The study cited as reference 71 (PMID 31792460) includes a larger patient cohort than the one used in the present manuscript. The authors should clarify why a smaller subset was selected

and provide a rationale for this sampling strategy. (ii). Leveraging additional publicly available datasets (e.g., PMID 37037616, PMID 26359337, PMID 30753825, etc.) and conducting multivariate analyses would enable assessment of whether the associations between individual gene-signature scores and immunotherapy response remain independent of one another, and of other known clinical covariates, thereby providing a clearer picture of the distinct predictive value of the signatures identified by the authors.

8) Extended Data Fig. 1 currently displays data for only a subset of the cell lines. Please include all the cell lines in this figure (or in a supplemental table) so that readers can assess the overall data quality and gauge the robustness of results derived from any individual line or from downstream analyses.

9) The raw FASTQ files, processed count matrices, and cell-metadata are central to the study and would constitute a valuable resource for the community. However, the current Data-Availability statement does not guarantee that these data will be deposited, and there is no reason for withholding the analysis code during peer review. I recommend that the authors upload the raw and processed data, as well as the code, to appropriate public repositories.

Minor comments:

1) In the first Main paragraph, the authors wrote that "analyses of primary tumors are often confounded by the presence of non-tumor components (e.g., stroma, immune cells, and adjacent normal tissue), making it difficult to define the intrinsic regulatory programs shared across cancer lineages." This statement is inaccurate if the focus is on "analyses". Modern snRNA-seq pipelines reliably distinguish malignant cells from non-malignant cells by exploiting copy-number variation profiles, and lineage-specific expression markers. Consequently, cancer-cell intrinsic programs can be inferred despite the presence of stromal and immune compartments. I recommend revising the sentence.

2) In the second Main paragraph, the authors state that "most studies rely on droplet-based platforms that are restricted to a single molecular modality or limited cell numbers per experiment, constraining the ability to map chromatin-gene regulatory relationships across diverse cancer states." The manuscript does not provide evidence that a large number of cells per experiment is required to increase discovery power in this context. In fact, cancer cell lines are considered relatively homogeneous compared with primary tumours or patient-derived samples. For such homogeneous systems, modest cell numbers are usually sufficient to capture the relevant regulatory variation. At a minimum, the authors should re-phrase the statement to acknowledge only the primary limitation. However, providing data that evaluate the association between increasing cell numbers and improved chromatin-gene regulatory mapping would be a better approach and could potentially help contextualize higher cell numbers as a key advantage of the study.

3) Define "GRN" (gene-regulatory network) at first use.

4) There is a typo linking CEBPB and UBE2V1 to chr11 rather than chr20

5) There is a typo the in x-axis of extended data fig 3f ('Backgroumd' instead of 'Background')

6) A citation appears to be missing after the sentence: "We next asked whether amplification of these genomic loci impacts drug response, given that cancer cell state is known to influence drug sensitivity."

7) Reference number 72 cite the Author Correction rather than the original manuscript.

8) Extended Data Fig. 8 a–d lacks information on what the displayed numbers represent (P-values) and does not indicate which statistical test was use

Reviewer #4

(Remarks to the Author)
This study presents a large-scale, multimodal single-cell atlas of 60 human cancer cell lines, leveraging an in-house EasySci platform to generate over 460,000 single-nucleus transcriptomic and chromatin accessibility profiles. By integrating RNA and ATAC data, the authors systematically construct pan-cancer gene regulatory networks (GRNs), delineate conserved epithelial–mesenchymal transition (EMT) states across lineages, and perform an in-depth comparative analysis of acral melanoma (AM) versus cutaneous melanoma (CM). The study further links in vitro regulatory features to tumor microenvironment (TME) composition and immunotherapy responses, demonstrating the translational potential of cancer cell line–derived regulatory signatures.There are several limitations to be revised.
1. Although cell lines enable the isolation of tumor-intrinsic programs,they can not define the TME interactions such as fibroblasts, immune cells,and ECM. Also the regulatory networks and pathways can not be demonstrated or validations.
2. The data lack spatial transcriptomics or imaging-based validation.Without spatial data and evaluation, it is difficult to ascertain whether ligand–receptor pairs are expressed in physically proximate cells.
3. Copy number variations were inferred from RNA expression using InferCNV. It has variable accuracy. Should chose whole-genome sequencing (WGS) or single-cell DNA-seq.
4. No validation for almost all the data. Make an example, CEBPB was proposed as an epithelial-state gate keeper based on in silico knockout. Please chose experimental perturbation methods to validate.
5. The single-cell TME data were derived from a single published study (GSE215121). This dataset includes both AM and CM patients,but the ethnicities and treatment backgrounds are unknown.

6. The main problem is almost all the results based on cell lines data without tumor tissue sequencing data.

Version 1:

Reviewer comments:

Reviewer #1

(Remarks to the Author)
I appreciate the authors' careful and comprehensive responses to the first-round comments. The revised manuscript is substantially improved, and the authors have done an excellent job addressing the prior concerns through additional clarification, analyses, and revisions.

Overall, I find the study to be rigorous, clearly presented, and of high value as a resource for the community. I have no additional major concerns and support publication of the manuscript.

(Remarks on code availability)
The authors have made the code available, and it appears to be a useful and reasonably organized resource accompanying the manuscript.

Reviewer #2

(Remarks to the Author)
I co-reviewed this manuscript with one of the reviewers who provided the listed reports. This is part of the Nature Communications initiative to facilitate training in peer review and to provide appropriate recognition for Early Career Researchers who co-review manuscripts.

(Remarks on code availability)
No, the code does not provide a README file. In addition, it is not clear based on the code availability how one would reproduce all analyses performed. Minimal R and python functions are currently present on the repository.

Reviewer #3

(Remarks to the Author)
The authors have addressed all my comments thoroughly and effectively.
I have no further comments.

(Remarks on code availability)
The scripts are available. To improve transparency and clarity, consider providing additional information in the GitHub repository, such as indicating which scripts correspond to which figures and specifying where the source data (e.g., input files for each script) are located.

Reviewer #4

(Remarks to the Author)
The revised version answer my concerns.

(Remarks on code availability)
The revised version answered my concerns.

Open Access This Peer Review File is licensed under a Creative Commons Attribution 4.0 International License, which permits use, sharing, adaptation, distribution and reproduction in any medium or format, as long as you give appropriate credit to the original author(s) and the source, provide a link to the Creative Commons license, and indicate if changes were made.
In cases where reviewers are anonymous, credit should be given to 'Anonymous Referee' and the source.
The images or other third party material in this Peer Review File are included in the article's Creative Commons license, unless indicated otherwise in a credit line to the material. If material is not included in the article's Creative Commons license and your intended use is not permitted by statutory regulation or exceeds the permitted use, you will need to obtain permission directly from the copyright holder.
To view a copy of this license, visit https://creativecommons.org/licenses/by/4.0/

REVIEWER COMMENTS

Reviewer #1, expertise in single cell RNA-seq, single cell ATAC-seq and melanoma TME (Remarks to the Author):

Summary

Xu and colleagues present a single-cell multi-omic atlas of 60 human cancer cell lines spanning 20 cancer types, profiled using their EasySci platform for parallel RNA-seq and ATAC-seq. Through integrative analysis, the authors construct pan-cancer gene regulatory networks, identify conserved EMT-associated states across lineages, link copy number variations to cell state transitions. They further demonstrate that these in vitro-derived signatures correlate with tumor microenvironment composition and immunotherapy responses in patient cohorts.

General Remarks

This manuscript should be accepted with revisions. The integration of matched transcriptomic and epigenetic profiles across such a diverse panel of cell lines enables novel insights into regulatory mechanisms that would be difficult to obtain from primary tumor data alone and from one cancer type. The validation of in vitro findings using external perturbation datasets (CRISPRa/i screens, ENCODE ChIP-seq) and patient cohorts (TCGA, immunotherapy trials) strengthens the conclusions. However, the manuscript's current presentation poses challenges for readability and accessibility, particularly for readers not deeply versed in computational single-cell methods.

Several key methodological clarifications are needed. First, the authors should more explicitly justify why single-cell resolution was essential for their findings, given that many analyses present averaged expression across meta-cells or cell lines.

We thank the reviewer for this important suggestion. We agree that we should more explicitly explain why single-cell resolution was essential for the analyses and conclusions.

Although many figures are summarized at the cell-line level for interpretability, the underlying analyses were performed at single-cell (**Fig. 1c-e, Fig. 2d-f, Fig. 4a-b**) or meta-cell resolution (**Fig. 2a-c, g-j, Fig. 3h, Fig. 4c**). This distinction is important because bulk or cell-line-averaged profiles would collapse intra-cell-line heterogeneity, provide a limited number of biological units for statistical analysis, and obscure subtle regulatory dynamics.

For example, single-cell resolution was critical for GRN inference. Our GRN analysis relies on coordinated variation between chromatin accessibility and gene expression across subtle cellular states[1,2]. Instead of aggregating all cells from the same cell line into one profile, our meta-cell strategy linked chromatin accessibility to gene expression, preserved local cell-state variation (**Extended Data Fig. 2k**) while reducing sparsity effects from single-cell assays (as discussed in **Extended Data Fig. 3** in SEACells algorithm[3]).

To directly test this, we performed pseudobulk benchmarking by aggregating all cells from each

cell line into one RNA profile and one ATAC profile, and then reran SCENIC+ using the same peak set. Pseudobulk analysis resulted in a substantial loss of information (**Fig. R1-1a**): many well-established lineage- and state-specific regulators identified in the single-cell-based GRN, including PAX3 and SOX10 in melanoma, PHOX2B and POU3F2 in neuroblastoma, FOXA1 in prostate cancer, IKZF1 in lymphoid malignancies, and EMT regulators such as FOSL1, ZEB1, and FOXC1, were no longer recovered. In contrast, evenly sampling only 10% of cells from each cell line for meta-cell-level analysis already retained substantial power for TF regulator identification (**Fig. R1-1b**). These results show that single-cell profiling is required to preserve regulatory heterogeneity, and thus to recover the full set of lineage- and state-specific regulators.

We further compared the power of target-gene identification across these analyses. Increasing the number of sampled cells gradually improved the accuracy of target-gene prediction (**Fig. R1-1c**). By contrast, the pseudobulk strategy, despite retaining the full sequencing depth from all cells, removed intra-cell-line heterogeneity and showed the weakest overlap with the original GRN-derived target-gene assignments.

We observed a similar pattern for melanoma program decomposition. Using the same gene feature set, we repeatedly ran NMF on either single-cell profiles or pseudobulk RNA profiles across melanoma cell lines. Because NMF involves random initialization, the stability of inferred programs across repeated runs directly affects their biological interpretability. We therefore quantified program stability using silhouette scores, where values closer to 1 indicate more consistent recovery of similar programs across random initializations, whereas values closer to 0 indicate poorly separated program assignments. The single-cell-derived decomposition was substantially more stable than the pseudobulk-derived decomposition, with silhouette scores of 0.592 and 0.111 (**Fig. R1-1d**), respectively. This supports the conclusion that the richer gene covariation structure captured by single-cell profiles substantially improved the robustness and interpretability of melanoma gene program identification.

Together, these analyses demonstrate that single-cell resolution was essential for the quality and interpretability of our analyses. Beyond the inter-cell-line comparisons we emphasize, our single-cell profiling also revealed specific intra-cell-line state heterogeneity, as shown for example in **Extended Data Fig. 2g**. While complementary to existing bulk measurements, single-cell resolution was necessary for the regulatory analyses that define this resource.

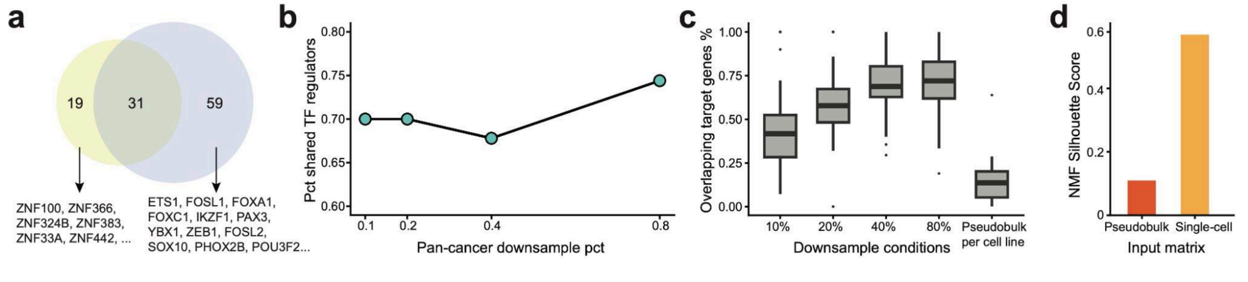

Fig. R1-1. Evaluation of single-cell-based analyses.

a, Venn diagram showing the overlap of GRN TF regulators identified using pseudobulked data

(yellow) or the original single-cell-derived meta-cells (blue).
b, Fraction of TF regulators shared with the full dataset across pan-cancer downsampling fractions.
c, Fraction of regulon target genes shared with the full dataset across pan-cancer downsampling fractions and pseudobulked data.
d, NMF silhouette scores comparing program stability from pseudobulked and single-cell input matrices of melanoma cell lines.

For example, single-cell data revealed that even within established lines, cells occupy distinct states along the EMT continuum.

We agree that intra-cell-line heterogeneity exists and that single-cell data provide an important opportunity to examine whether established cell lines contain distinct cellular states along the EMT continuum. As shown in **Fig. 2h**, the overall EMT spectrum in our dataset was primarily explained by inter-cell-line heterogeneity. Nevertheless, cells from the same cell line also showed variation in EMT pseudotime, although this variation was generally more restricted than the differences observed across cell lines.

To directly quantify whether EMT-associated heterogeneity is detectable within individual cell lines, we devised an analysis to measure the separation between EMT-high and EMT-low cell-state centers within each cell line in the low-dimensional manifold (**Fig. R1-2a**). Specifically, for each cell line, we compared the observed distance between EMT-high and EMT-low centers with a background distribution generated by random partitioning of cells from the same cell line. Greater-than-background separation would indicate that EMT variation contributes to structured, rather than random, intra-cell-line state heterogeneity (**Fig. R1-2b-c**). Using this approach, all cell lines from our GRN cluster 6-8 (**Fig. 2b**) except SK-CO-01 and MDA-231-TGL showed strong statistical significance (**Fig. R1-2b**), supporting EMT as a detectable and biologically interpretable source of intra-cell-line heterogeneity.

We further examined how this EMT-associated heterogeneity relates to cell-state organization on the UMAP (**Fig. R1-2d**). SK-CO-01, which showed no significant intra-cell-line EMT separation, displayed a relatively diffuse distribution without an obvious EMT-associated structure. In contrast, H2030-TGL and H2087-LCC1 showed the two largest intra-cell-line EMT deviations across the dataset, but with distinct patterns. In H2030-TGL, cells in a more mesenchymal state formed a discrete cluster, whereas H2087-LCC1 showed a more continuous transition. The distinct heterogeneity observed in H2030-TGL is consistent with previous reports of cell-state heterogeneity in this cell line (**Fig. S3b** in PMID: 37433798), which could not be explained by technical or quality-related factors during preprocessing (**Extended Data Fig. 2g-j**). Moreover, both the conserved EMT signature (PMID: 32358524) and the DEG signature of GRN cluster 8 that we identified (**Fig. 2e**) showed strong concordance on the UMAP, supporting a consistent biological interpretation of these intra-cell-line state differences.

Finally, to test whether these intra-cell-line states are also supported at the epigenetic level, we compared chromatin accessibility features between H2030-TGL cells from distinct states (**Fig.**

R1-2e). We observed regulatory differences consistent with their inferred relative EMT states (**Fig. R1-2f**). Peaks activated in cluster 1, corresponding to the more mesenchymal state, were associated with HCFC2 regulation, which we identified as activated along the EMT continuum (**Fig. 2j**). In contrast, peaks activated in cluster 0, corresponding to the more epithelial state, were associated with CEBPB, FOXA1, GRHL2, IRF2, and PAX2 regulons, which were identified in our study as representative regulators of the epithelial state (**Fig. 2c, i-j**).

Together, these results support EMT as a contributor to intra-cell-line heterogeneity and demonstrate that regulatory programs identified at the pan-cancer scale can also explain structured cell-state variation within individual cell lines. We have included these analyses in our manuscript (line 267-275, **Extended Data Fig. 4**).

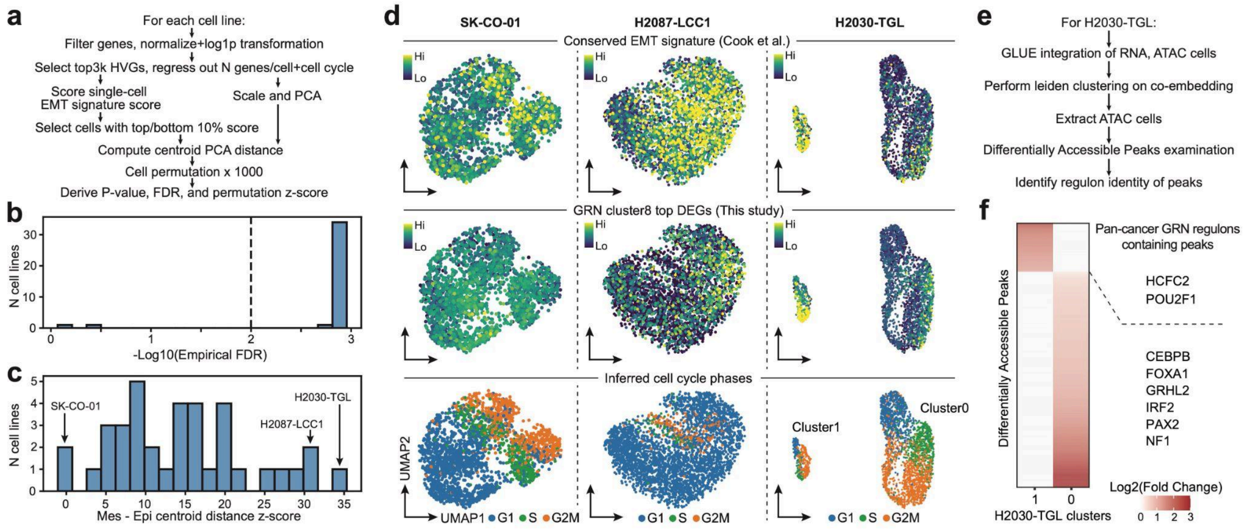

Fig. R1-2. EMT-associated intra-cell-line heterogeneity and regulatory validation.
a, Schematic of the workflow used to quantify EMT-associated heterogeneity within each cell line.
b, Distribution of empirical FDR values across cell lines.
c, Distribution of permutation-based z-scores for EMT-high versus EMT-low centroid distances, highlighting SK-CO-01, H2087-LCC1, and H2030-TGL as representative examples.
d, UMAP visualization of representative cell lines colored by conserved EMT signature, GRN cluster 8 DEG signature, and inferred cell-cycle phase. Score distributions for visualization were scaled separately within each cell line.
e, Schematic of the workflow used to identify differentially accessible peaks and associated regulons between H2030-TGL intra-cell-line states.
f, Heatmap of differentially accessible peaks in two H2030-TGL clusters, and associated pan-cancer GRN regulons containing these peaks.

And enabled deconvolution of mixed phenotypic states within individual melanoma lines.

We thank the reviewer for the insight. We would like to clarify that our goal was not to assign definitive subtype identities to individual melanoma cells, as their original labels are derived from

their clinical source. Instead, our analyses support a continuous state-transition spectrum between acral-like and cutaneous-like transcriptional programs, rather than discrete assignment of individual cells to definitive melanoma subtypes.

We examined the distribution of single-cell scores across melanoma cell lines using subtype-associated gene programs (Program 11, AM-universal; Program 4, CM-universal; **Fig. R1-3a**). Acral melanoma cell lines generally showed higher acral-like - cutaneous-like program scores than cutaneous melanoma cell lines. At the same time, intra-cell-line heterogeneity was also observed, although the score distributions within each cell line were generally more confined than the differences observed across cell lines. These results suggest that most cells within a given melanoma line occupy a broadly similar position along the acral-like to cutaneous-like spectrum, while still retaining measurable intra-cell-line state variation.

To further examine whether this intra-cell-line heterogeneity was associated with structured cell-state organization, we applied the same centroid-separation strategy described in **Fig. R1-2a**. All melanoma cell lines showed significant acral-like versus cutaneous-like separation (**Fig. R1-3b**), and large permutation-based z-scores further supported the presence of structured intra-cell-line melanoma state heterogeneity (**Fig. R1-3c**). For example, in MeWo, MB4667, and M040204, the Program 11 minus Program 4 score showed a gradual continuum on the UMAP after regressing out cell-cycle effects (**Fig. R1-3d**), supporting acral-like to cutaneous-like state variation as a prominent and structured source of intra-cell-line heterogeneity.

In summary, these analyses support the presence of a conserved acral-like to cutaneous-like state axis across melanoma cell lines. Although each cell line tends to occupy a dominant position along this spectrum, single-cell profiling reveals additional intra-cell-line heterogeneity that reflects continuous melanoma cell-state plasticity. We have included these analyses in our manuscript (line 619-621, **Extended Data Fig. 7j-m**).

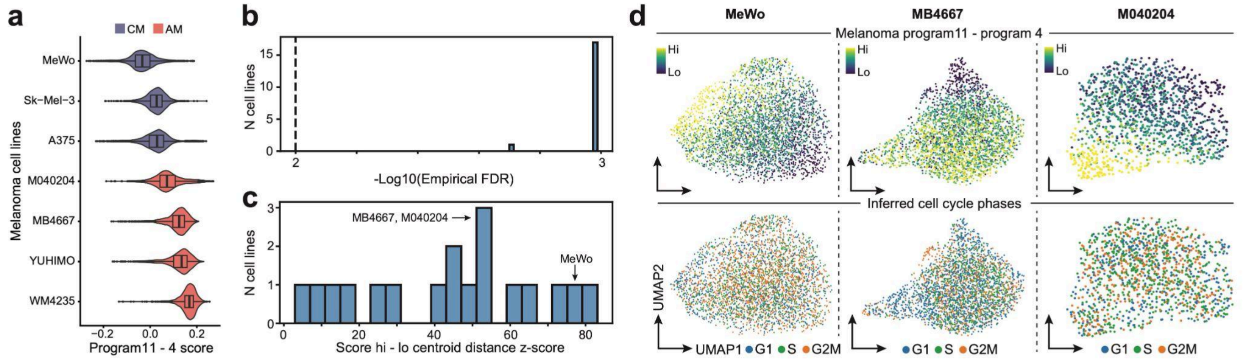

Fig. R1-3. Melanoma intra-cell-line heterogeneity examination.
a, Distribution of single-cell Program 11 minus Program 4 scores across example melanoma cell lines.
b, Distribution of empirical FDR values across melanoma cell lines, supporting significant separation between acral-like and cutaneous-like cell-state centers.

c, Distribution of permutation-based z-scores for acral-like versus cutaneous-like centroid distances across melanoma cell lines, highlighting MB4667, M040204, and MeWo as representative examples.
d, UMAP visualization of representative melanoma cell lines colored by Program 11 minus Program 4 score and inferred cell-cycle phase, showing structured intra-cell-line acral-like to cutaneous-like variation that is not explained by cell cycle. Score distributions for visualization were scaled separately within each cell line.

Second, the CNV analysis relies on transcriptome-inferred copy number variations (using InferCNV) without validation from orthogonal DNA-based methods such as bulk whole-genome sequencing or array data from the Cancer Cell Line Encyclopedia. These clarifications would strengthen confidence in the CNV-EMT associations and help readers understand the specific advantages of the single-cell approach.

We thank the reviewer for highlighting this important methodological consideration. We agree that independent validation can further strengthen CNV calls inferred from transcriptomic profiles. Single-cell RNA-seq- and ATAC-seq-based CNV inference has been broadly applied to detect large-scale copy-number alterations in cancer cells[4–6], as well as to distinguish malignant and non-malignant populations from CNV patterns in an unsupervised manner[7–9]. In response to the comment, we have validated these inferCNV results through several orthogonal approaches.

In the original manuscript, we have evaluated the reliability of our inferCNV results using two orthogonal criteria: the inferred CNV profile of IMR90 as a diploid negative control, and the concordance between inferred CNV regions and ATAC-derived chromatin accessibility signals. IMR90 showed minimal inferred CNV signal (**Extended Data Fig. 5a**), supporting the reliability of the baseline. For inferred amplification events, we retained two regions with substantial overlap with elevated ATAC signals and excluded a regional amplification pattern lacking such orthogonal support (**Extended Data Fig. 6g-i**). ATAC accessibility analysis also independently highlighted elevated signals at chr11 and chr20 hotspot regions (**Fig. 2f**), as reflected by enriched signals around genes located within these regions, such as CEBPB, B4GALT5, and UBE2V1 in the chr20 hotspot and CCND1 and TPCN2 in the chr11 hotspot.

To directly benchmark our CNV inference, we compared cell line-specific genome-wide inferCNV profiles with publicly available CNV profiles derived from WGS or WES. We chose these published sources over CCLE SNP-array data because several of our metastatic-derivative lines are not represented in CCLE, and they provide higher-resolution copy-number estimates. CNV profiles of 786-M1A, H2030-BrM3, MDA231-BrM2, and PC9 from WES[10] showed strong concordance with our inferCNV results (**Fig. R1-4a**), despite extensive differences in laboratory source, profiling technology, and computational method. In addition, chr20 hotspot amplifications inferred by inferCNV in the epithelial-state cell lines SK-BR-03 and HT-29 were recapitulated by independent WGS datasets[11,12] (**Fig. R1-4b-c**).

Together, these analyses support the robustness of our CNV inference strategy for identifying

broad copy-number patterns within our single-cell multimodal resource. We agree that direct DNA-based profiling would provide higher-resolution validation and remains an important future direction. At the same time, CNV inference from large-scale single-cell RNA or ATAC data provides a high-throughput and accessible strategy for screening broad copy-number alterations and nominating candidate CNV-associated regulatory features for further validation (**Extended Data Fig. 5**).

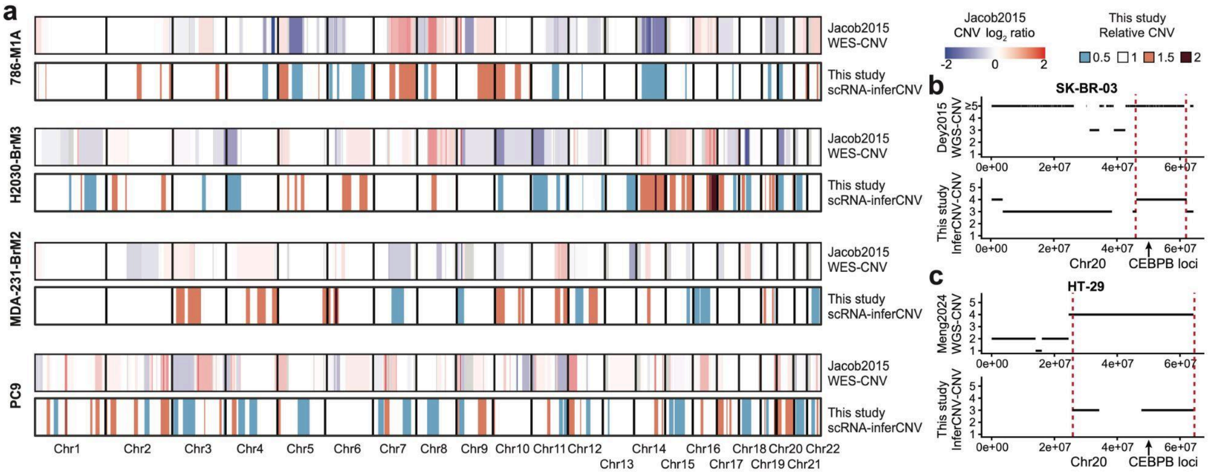

Fig. R1-4. Validation of scRNA-seq-based CNV inference.

a, Comparison of genome-wide CNV profiles inferred from scRNA-seq in this study with published WES-derived CNV profiles from Jacob et al. for 786-M1A, H2030-BrM3, MDA231-BrM2, and PC9.

b, Comparison of chr20 amplifications in SK-BR-03 between published WGS-derived CNV data and scRNA-seq-based inferCNV from this study.

c, Comparison of chr20 CNV profiles in HT-29 between published WGS-derived CNV data and scRNA-seq-based inferCNV from this study.

The following recommendations aim to enhance clarity and impact for a broader audience.

Major/Minor Points

Abstract

1. The abstract's transition from pan-cancer findings to the focused melanoma analysis feels abrupt. The authors state they performed a "focused analysis of acral and cutaneous melanoma" without explaining why these subtypes warranted special attention. The current framing misses an opportunity to highlight the significance of including rare cancer subtypes in large-scale atlases.

We thank the reviewer for the constructive comment. We agree that the previous narrative was abrupt and did not sufficiently highlight the inclusion of rare cancer subtypes.

We have revised the abstract to smooth the transition and to highlight the inclusion of rare cancer subtypes (line29-32): “*To further examine how regulatory programs diverge within a*

cancer lineage and contribute to clinically divergent outcomes, we performed a focused comparison of cutaneous melanoma with acral melanoma, a rare, UV-independent subtype underrepresented in existing pan-cancer atlases."

We also highlighted the inclusion of rare cancer subtypes in the resource and our ability to identify subtype-specific regulatory features in the abstract (line 37-40): "*Together, by extending single-cell multi-omic profiling to rare alongside common cancer subtypes, this atlas offers a resource for mapping pan-cancer and subtype-specific gene-regulatory programs that shape cancer cell-state plasticity*."

Introduction

1. The EasySci platform is central to the experimental approach, enabling parallel single-cell RNA and ATAC profiling across many samples. However, the Introduction does not introduce this technology or explain why it is particularly beneficial for pan-cancer analysis. Readers unfamiliar with the method are left wondering how the authors achieved such scale.

We thank the reviewer for raising this important point. We agree that EasySci[13], the platform that enabled this pan-cancer atlas, was not adequately introduced in the original manuscript. Briefly, it is a combinatorial-indexing single-cell genomics platform developed by our lab that offers high scalability and sample-multiplexing capability. These capabilities are especially valuable for profiling diverse sample panels, such as the cancer cell line atlas constructed here. Common 10x Genomics workflows require either splitting samples across channels, which can introduce batch effects, or sample hashing with oligo-conjugated antibodies, which adds experimental and computational complexity. EasySci avoids these tradeoffs by barcoding samples in parallel microwells during reverse transcription, then pooling them for shared downstream processing, minimizing technical variation.

We have added the following text to the Introduction (line 69-71): "*To address this gap, we used EasySci, a combinatorial-indexing single-cell genomics platform that offers high scalability and sample-multiplexing capability, to construct a pan-cancer single-cell atlas…*"

Results

1. The Results section mentions EasySci only in passing (Figure 1a legend) without explaining its significance or advantages. For readers unfamiliar with single-cell methods, the technical achievement here may be underappreciated.

We appreciate this opportunity to clarify the platform's advantages. To do so, we have added the following technical description to the Results section (line 88-91): "*Based on combinatorial indexing, EasySci enables parallel sample barcoding through indexed reverse transcription of different samples in separate wells of a plate. Subsequent split-pool barcoding exponentially expands the cell barcode space, enabling profiling of millions of cells in a single experiment. These features simplify multi-sample profiling and minimize technical batch effects*."

2. Figure 1c-d is visually overwhelming. The current presentation uses 60+ colors for individual cell lines, making it nearly impossible to identify which cell lines cluster together or discern tissue-of-origin patterns. The reader cannot meaningfully interpret these figures.

We thank the reviewer for this helpful comment. We agree that the color palette is visually complex, given the need to distinguish 60 individual cell lines in the UMAP visualization. To address this, we directly annotated each cluster on the UMAP with the corresponding cell line name and matched color. This approach is easier to scan than cross-referencing a separate legend. We have updated the figure legend to reflect the purpose of our annotation strategy. We welcome further suggestions from the reviewer for refining this visualization.

3. The concept of "meta-cells" (aggregating neighboring cells for multimodal analysis) is introduced without sufficient explanation for non-specialist readers. The rationale for why this aggregation is necessary for GRN construction and how it preserves biological variation while reducing sparsity is not clearly stated.

We thank the reviewer for this helpful suggestion. Gene regulatory network construction requires linked chromatin accessibility and gene expression measurements to map regulatory elements to their target genes. Meta-cell construction is a widely used strategy for linking these modalities. This is necessary in our EasySci data, where RNA and chromatin accessibility are profiled in different cells, so that no single cell carries both measurements. We used the SEACells algorithm for meta-cell construction. As benchmarked in that study (**Extended Data Fig. 3** in[3]), aggregating similar cells substantially improves the concordance between chromatin accessibility and gene expression, facilitating the detection of coordinated chromatin and transcriptional programs. To improve clarity, we have added the following description to the Results section (line 150-159):

"*In this approach, single-cell transcriptomes and chromatin accessibility profiles were first integrated into a shared low-dimensional space, after which transcriptionally and epigenetically similar neighboring cells were identified and grouped to form 'meta-cells'. Each meta-cell therefore represents the multi-modal profile of a local neighborhood of cells. By aggregating counts from locally similar cells, this approach links transcriptomic and chromatin states, reduces dropout-driven sparsity at each measurement point, and preserves cell-state heterogeneity at the meta-cell level*".

4. The description of TF regulon identification and clustering (Figure 2b-c) is dense. The text states that 92 activating TF regulons were used for hierarchical clustering, but it is unclear how these 92 were selected from the larger network and what "activating" means in this context.

We thank the reviewer for raising these two important points about regulon selection and our definition of "activating" regulons. To clarify, the 92 activating regulons were not cherry-picked. From the full GRN, we retained all regulons with positive (activating) associations between TF expression and target genes and between cis-regulatory element accessibility and target genes, and used all 92 for clustering without further sub-selection. We focused on activating regulons

because they correspond to the canonical, more interpretable mode of TF-mediated regulation (detailed below).

Regarding the term "activating", in SCENIC+, both positive and negative associations are retained when inferring links between TF expression and target genes, and between cis-regulatory element accessibility and target genes. Although TFs can act through diverse mechanisms, the canonical model is that TF binding promotes accessibility of cis-regulatory elements, which in turn facilitates target gene expression. We therefore restricted our analysis to these activating regulons, as now stated explicitly in the main text (lines 172–177):

"*To classify cell lines by their regulatory programs, we focused on activating regulons, defined as those in which TF expression is positively associated with target gene expression, and the accessibility of TF motif-containing cis-regulatory elements is also positively associated with target gene expression. Scoring pan-cancer cell lines with these 92 activating TF regulons, followed by hierarchical clustering, identified 8 clusters of cancer cell lines with distinct gene regulatory programs*."

5. Make sure you are extremely clear in the figures which data analysis are what you performed versus what are the validation sets.

We thank the reviewer for this valuable suggestion. To improve clarity, we have added labels throughout the figures to distinguish analyses performed on our dataset from those using external validation datasets. Specifically, figure legends now identify the data source for each panel, with external validation datasets, including tissue single-cell RNA-seq reference, ENCODE ChIP-seq, CRISPRa and CRISPRi Perturb-seq, CCLE, and TCGA, and patient TME single-cell RNA-seq. As examples, in Fig. 3, panels based on external datasets, including CCLE drug-sensitivity profiles (**Fig. 3f**), external cell-line transcriptomes and ENCODE CEBPB ChIP-seq (**Fig. 3i–j**), CRISPRa and CRISPRi Perturb-seq data (**Fig. 3k-p**), are now explicitly labeled to distinguish them from analyses performed on our atlas. Likewise, in Fig. 5, panels derived from TCGA-SKCM bulk RNA-seq (**Fig. 5b,c**), published patient single-cell RNA-seq (**Fig. 5d–f**), and immunotherapy patient cohorts (**Fig. 5h–j**) are marked as external, whereas cell-line-derived analyses (**Fig. 5g**) are labeled as generated in this study.

6. Figure 2d shows mapping of lung and breast cancer cell lines to normal reference cell types. The text states that cells were "mapped," but the method is not clearly explained in the Results. The concept of projecting cancer cells onto normal developmental trajectories is powerful but requires clearer exposition.

We thank the reviewer for highlighting the need to explain this approach more clearly. To examine whether cancer cell lines may resemble specific cell types from their corresponding normal tissues, we implemented the ingest function in Scanpy, which maps query single-cell transcriptomes onto a reference single-cell RNA-seq dataset and transfers the annotation label of the most transcriptionally similar reference cells. As references, we used published single-cell atlases of normal human lung (Sikkema et al.[14]) and breast (Kumar et al.[15]).

In brief, query transcriptomes were normalized and transformed using the same gene features as the reference dataset, and then projected into the reference PCA space using the precomputed PCs and gene loadings. Each query cell was then assigned the cell-type label of its nearest reference neighbors. This mapping yielded biologically interpretable assignments; for example, SK-BR-3 cells were predominantly mapped to LumHR cells, consistent with a recent study identifying LumHR cells as a potential cell of origin for ER^+ breast cancer[16]. More broadly, the mapping revealed a coherent progression across clusters 6–8: cluster 6 cell lines mapped to specialized epithelial cell types, whereas cluster 8 cell lines, including triple-negative MDA-MB lines, mapped almost entirely to a fibroblast-like mesenchymal state. This indicates that cancer cell lines retain lineage features of their tissue of origin while spanning an epithelial-to-mesenchymal continuum.

We note that this approach identifies the closest normal counterpart for each cancer cell rather than implying identity with normal cells, thereby highlighting retained lineage features within the malignant state. To clarify this analysis, we have added the following explanation to the Results section (lines 207–212):

"*To further dissect the phenotypes of these pan-cancer conserved cell states, we implemented a nearest-neighbor-based annotation label transfer strategy. In brief, query cells from lung and breast cancer cell lines within clusters 6–8 were projected into a shared transcriptional space with reference single-cell RNA-seq atlases of normal lung and breast tissues. Cell-type annotations were then assigned to query cancer cells based on the labels of their nearest neighbors among the reference cells.*"

7. The use of cytokine-treated cell line panels as a reference for EMT signature genes is introduced without justification. Readers may question why signatures from experimentally induced EMT are appropriate for analyzing basal heterogeneity across cancer cell lines.

We thank the reviewer for raising this important point. We fully agree that EMT programs can vary substantially across contexts. Indeed, the original study[17] presenting this signature acknowledged the strong context specificity of EMT. To enhance generalizability, the conserved EMT signature was defined using genes that change consistently across most conditions, capturing EMT features shared across diverse contexts. In addition, these cytokine-induced transcriptomic responses from multiple cell lines were benchmarked against the widely recognized HALLMARK EMT signature (**Fig. 1f** in[17]), confirming robust induction of the EMT phenotype. Because these genes reflect convergent EMT dynamics rather than cytokine-specific responses, they remain informative for the basal EMT-like heterogeneity analyzed here, despite the signature having been derived under induced conditions.

Importantly, rather than relying heavily on this signature, we used it only as an orthogonal check on the cell states identified *de novo* in our GRN analysis (**Fig. 2b**). We observed strong consistency between our GRN-based cell state definition and the trajectory inferred using this conserved EMT signature (**Fig. 2h**). The agreement between these two independent analyses

supports the robustness of our EMT-state definitions. As additional support, IMR90, a diploid human fibroblast cell line in our atlas, falls at the terminal mesenchymal end of this trajectory, consistent with its fibroblast identity. To make this framing explicit, we have added the following statement to the Results section (line 239-243):

"*To further confirm the generalizability and robustness of our epigenetically and transcriptionally defined EMT-like states, we utilized a recently defined conserved EMT signature derived from diverse cell lines treated with pro-EMT cytokines. This signature captures genes consistently induced across multiple EMT-promoting conditions and was used here as an orthogonal validation.*"

8. The relationship between "GRN-based clustering" and "RNA-based clustering" (Figure 2h) is mentioned but not clearly distinguished. It is unclear whether these are independent classifications or whether one informed the other.

We thank the reviewer for pointing out this ambiguity. The "RNA-based clustering" labeled in the original **Fig. 2h** refers to the EMT pseudotime. It is fully orthogonal to and independent from the GRN-based clustering, and the agreement between the two in **Fig. 2h** supports the generalizability of our de novo EMT definitions. GRN-based clustering was derived from TF regulon activities in our pan-cancer GRN (**Fig. 2b**), whereas EMT pseudotime was inferred from the conserved EMT signature derived from cytokine-treated cell lines[17]. Despite their independent derivation, the two align: cell lines in the GRN-defined epithelial cluster fall near the root of the EMT trajectory, and those in the GRN-defined mesenchymal cluster toward its terminal end, consistent with our EMT-state definitions. This alignment also suggests shared epigenetic and gene-regulatory features across cancer contexts.

To clarify, we have revised the axis and legend labels in Fig. 2g-j and added the following explanation to the Results section (line 243-246):

"*Cells in our study aligned along a continuous trajectory defined by the conserved EMT signature derived from distinct cancer cell lines, and their pseudotime distributions closely paralleled the GRN-based clustering independently defined by our epigenetic and gene expression association analysis.*"

9. The term "genomic hotspot loci" is introduced (Figure 3c) but not defined. Are these known cancer-associated amplicons, or are they novel regions identified in this study? The distinction between expected findings and novel discoveries is unclear.

We thank the reviewer for pointing this out. In our study, the term "genomic hotspot loci" refers to recurrently amplified genomic regions whose copy-number changes were linked to the EMT state in our study. Specifically, these regions correspond to loci on chromosomes 11 and 20 identified in our analysis. We did not intend to claim them as newly discovered amplicons, rather, the novel aspect of our finding is their specific association with the epithelial EMT state. To avoid ambiguity, we have added the following definition to the Results section (lines

410–412):

"*As expected for genes affected by regional genetic alterations, these genes were concentrated in two specific genomic regions on chromosomes 11 and 20, respectively, which we refer to here as genomic hotspot loci*."

10. Figure 3d shows CNV patterns across epithelial, intermediate, and mesenchymal states, but the visual presentation makes it difficult to see the pattern being described—that amplifications are present in epithelial states but absent in mesenchymal states. The heatmap colors and organization do not clearly convey this trend.

We thank the reviewer and agree that the original Fig. 3d did not clearly convey this pattern. It was originally designed to show the global CNV patterns of representative cell lines across epithelial, intermediate, and mesenchymal states. However, at the whole-genome scale, the two regions highlighted in Fig. 3c are too small to make the state-associated amplification visible. In particular, prominent amplification of these two regions was restricted to epithelial states and absent in mesenchymal states. To make this pattern clearer, we have revised **Fig. 3d** to focus on these two chromosomes rather than the full genome-wide landscape, so that the epithelial-specific amplification is now directly visible.

11. The in silico knockout and Perturb-seq validation sections (Figure 3h-p) are dense and contain multiple interconnected analyses. The current flow is difficult to follow, especially for readers not familiar with perturbation screening approaches.

We thank the reviewer for this helpful comment. To improve readability, we have comprehensively revised this section so that the analyses in **Fig. 3h-p** now follow a clear logical progression. Specifically, in silico knockout first nominates CEBPB as an epithelial-state regulator (**Fig. 3h**); ENCODE ChIP-seq and CRISPRa Perturb-seq then validate its binding and functional activity (**Fig. 3i-l**); and a CRISPRi Perturb-seq screen of the chromosome 11 hotspot validates additional candidate regulators (**Fig. 3m-p**). (line 439-482)

12. Figure 4b is difficult to interpret. The scatter plots show associations between oNMF-derived programs and established melanoma signatures, but the relationship between the two axes, what the error bars represent, and how to interpret the coloring are not immediately clear.

We thank the reviewer for helping us clarify this figure. In the original scatter plot, one axis shows an oNMF-derived gene program score and the other an established melanoma phenotypic signature score, both averaged per cell line, so that the position of each cell line reflects the association between the two. To highlight inter-cell-line heterogeneity, colors represent individual melanoma cell lines (matching the original Fig. 4a). Error bars indicate the standard deviation of scores across meta-cells within each cell line. Because the absolute values of these scores are generally less informative than their relative trends, we omitted the numerical axis scales to keep the focus on these associations.

We have replotted **Fig. 4b** and defined the axes and coloring to prioritize the inter-cell line difference.

13. Figures 4g-k would benefit from consistent color schemes to help readers track which elements correspond to AM versus CM across panels. Currently, each panel uses independent coloring, making it difficult to follow the regulatory logic being described.

We thank the reviewer for the constructive suggestion. We have updated the color palette of **Fig. 4**: we use red to represent acral melanoma, and blue to represent cutaneous melanoma.

14. The transition from in vitro cell line findings to patient TME and immunotherapy response is conceptually important but presented as a series of correlative analyses without explicit articulation of the hypotheses being tested.

We thank the reviewer for raising this important point. To improve the narrative flow, we have revised the manuscript to state the two hypotheses tested in this section explicitly. First, we examine whether melanoma subtype-defining cancer-intrinsic inflammatory programs observed *in vitro* are associated with distinct TME features in patient tumors. Second, because the tumor microenvironment shapes immunotherapy outcomes, we examine whether these subtype-specific gene-regulatory features predict immunotherapy response in patients. We also clarified the rationale for using TCGA SKCM bulk RNA-seq profiles and revised the text to better connect the TME composition and predicted neoantigen burden analyses to the first hypothesis. (Line 752-777).

15. The description of immune signature scoring in Figure 5d relies on broad, general terms ("reduced translation," "increased heat shock responses") that do not clearly connect to specific immune functions or anti-tumor mechanisms. The current phrasing assumes prior knowledge of how these signatures relate to immune function, which may not hold.

We thank the reviewer for this helpful comment. We agree that the original description relied on broad gene-program labels and did not sufficiently explain how these signatures relate to immune function. These annotations were derived from the recently developed TCAT framework[18], which defines recurrent T cell gene expression programs associated with T cell subsets, states, and functions across diverse single-cell datasets. Our original goal was to compare immune cell phenotypic states between AM and CM patients in an unbiased manner, complementary to the analysis of canonical functional marker expression.

To address the reviewer's concern, we revised this analysis and panels in three ways (**Fig. R1-5, Fig. 5d**). First, we removed broad T cell state annotations that were not directly interpretable in terms of specific immune functions from **Fig. 5d**, such as Translation, heat shock, and Immediate Early Genes (IEG). Second, to better match the context in which these recurrent T cell programs were defined, we restricted the TCAT-based scoring in **Fig. 5d** to T cell populations only, rather than applying these annotations across other immune and stromal lineages. Third, to characterize phenotypic changes in additional tumor microenvironmental cell

types, we performed AM-versus-CM differential expression analysis across all TME cell types and conducted functional enrichment analysis. As representative examples, macrophages and fibroblasts, two major components of the tumor microenvironment, are now shown in **Fig. 5e**.

In the revised **Fig. 5d**, Tregs and exhausted CD8 T cells from AM patients showed increased immunosuppressive/exhaustion-associated programs, and CD8 effector T cells, γδ T cells, and NK cells showed reduced cytotoxic programs. In the revised **Fig. 5e**, macrophages from AM patients showed enrichment of IL1 signaling and Senescence Associated Secretion Phenotype (SASP) programs, consistent with a Tumor Associated Macrophage (TAM)-like phenotype that may contribute to an immunosuppressive tumor microenvironment[19]. Fibroblasts from AM patients also showed an increased SASP secretion program, together with reduced focal adhesion and EGFR signaling, suggesting a shift toward an immunosuppressive Cancer Associated Fibroblast (CAF)-like state rather than a classic fibroblast phenotype[20]. We updated the main text accordingly (line 791-807). Together, these revisions provide a more direct connection between the scored gene programs and their potential immune relevance in the tumor microenvironment.

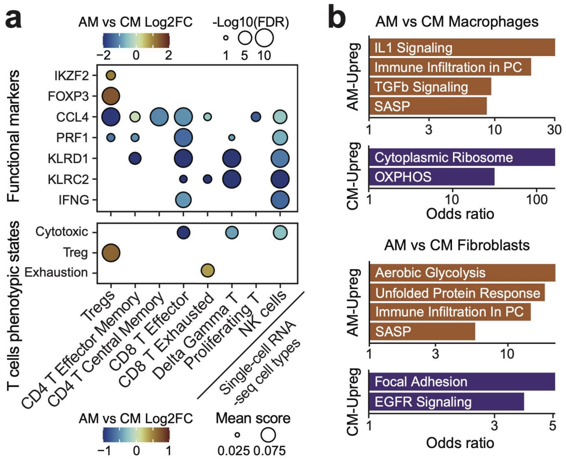

Figure R1-5. Revised characterization of AM- and CM-associated tumor microenvironmental cell states from single-cell RNA-seq data.

a, Dot plot showing AM-versus-CM log2 fold changes of selected functional markers (top) and TCAT-derived T cell phenotypic state scores (bottom) across T and NK cell populations; dot size represents statistical significance for marker expression or mean score for TCAT-derived state scores.

b, Bar plots showing top enriched functional terms among AM-upregulated and CM-upregulated differentially expressed genes in macrophages and fibroblasts.

Discussion

1. While the Discussion is comprehensive, several limitations and contextual considerations should be more explicitly addressed to provide a balanced interpretation

a. The study profiled 60 cell lines across 20 cancer types, which is substantial, but the Discussion does not acknowledge which cancer types were excluded or under-represented.

Readers may wonder about the generalizability of findings to cancers not included in the panel (e.g., pancreatic, gastric, bladder carcinomas).

We agree. While our panel enables comparative analysis across diverse cancer lineages, it does not capture the full diversity of human cancers. We have revised the Discussion to acknowledge the cancer types not represented in our panel and the resulting limits on generalizability (line 1047-1053):

"*Although we profiled 60 cell lines spanning 20 cancer types, our panel does not encompass the full diversity of human cancers. Several clinically important malignancies, such as pancreatic, gastric, and bladder carcinomas, were not represented, and therefore, the extent to which the regulatory programs identified here generalize to these contexts remains to be determined. Future studies incorporating broader cancer type coverage, additional genetic backgrounds, and primary tumor or patient-derived models will be important for defining which regulatory programs are broadly conserved versus lineage- or context-specific*."

b. The identification of 15 orthogonal gene programs from melanoma cell lines using oNMF is presented as a key step in defining AM- and CM-specific programs. However, the Discussion does not address how sensitive these programs might be to the number of programs chosen (k) or whether alternative program numbers would yield different subtype-specific signatures. This is particularly relevant because these programs later form the basis for TME correlations and immunotherapy predictions.

We thank the reviewer for raising this important point. We agree that the robustness of program decomposition is critical, as these programs serve as the basis for several downstream analyses. To address this concern, we performed additional benchmarking of the oNMF decomposition across multiple choices of program number, k, and evaluated the stability of the inferred programs from several complementary perspectives (Fig. R1-2).

First, to examine how well different values of k captured the underlying data structure, we performed NMF decomposition[21] across a range of k values (shown as **N** in **Fig. R1-6**) and compared matrix reconstruction accuracy (**Fig. R1-6a**). As expected, increasing k reduced the mean squared reconstruction error. However, the improvement became marginal after k>10, indicating that more than 10 programs were generally sufficient to capture the major structure of the data (**Fig. R1-6b**).

Second, we considered that increasing k may also introduce redundancy by over-separating genes with similar covariation patterns. To evaluate this, we computed correlations between gene program scores across melanoma cells and flagged program pairs with Pearson correlation coefficients greater than 0.65 as "redundant pairs." Decompositions with smaller k values showed strong orthogonality, while higher-resolution decompositions showed increasing redundancy (**Fig. R1-6c**). At k=15, only one correlated program pair was detected: programs 3 and 13, which remained biologically interpretable and corresponded to two YUSEEP cell line-specific programs (**Fig. 4a**). Thus, the k=15 decomposition preserved broad orthogonality

while providing sufficient resolution to capture biologically meaningful cell line- and subtype-associated programs.

Third, to directly visualize the conservation of gene program membership across different values of k, we generated a Sankey diagram showing how genes were grouped across decompositions (**Fig. R1-6d**). Gene groups were colored according to their program identity in the original k=15 decomposition. This analysis showed that many genes were not assigned to stable programs when k=5, whereas program identities became largely stable from k=10 to k=15. In contrast, further increasing k tended to split existing gene groups into smaller subprograms. Importantly, programs 4 and 11 in the k=15 decomposition, which were identified in the manuscript as cutaneous melanoma-specific and acral melanoma-specific programs, respectively, remained highly stable across different decompositions (**Fig. R1-6d**). This supports the robustness of the subtype-associated program identification.

Finally, because we observed that these decomposed programs captured melanoma phenotypic transitions[22–24] (**Fig. 4b**), we further benchmarked how well programs inferred from different k values represented known melanoma biology. Despite small differences in performance, different choices of k consistently recovered conserved gene programs corresponding to melanocytic, neural crest-like, and undifferentiated phenotypes across melanoma cell lines. These programs showed substantial overlap with previously characterized melanoma phenotypic signatures, further supporting the biological robustness of the decomposition.

Overall, these analyses suggest that our main conclusions are not highly sensitive to the exact choice of k. The N=15 decomposition provided a balanced resolution, capturing known melanoma phenotypic programs while avoiding extensive redundancy. We have added these analyses to the manuscript (line 598-599).

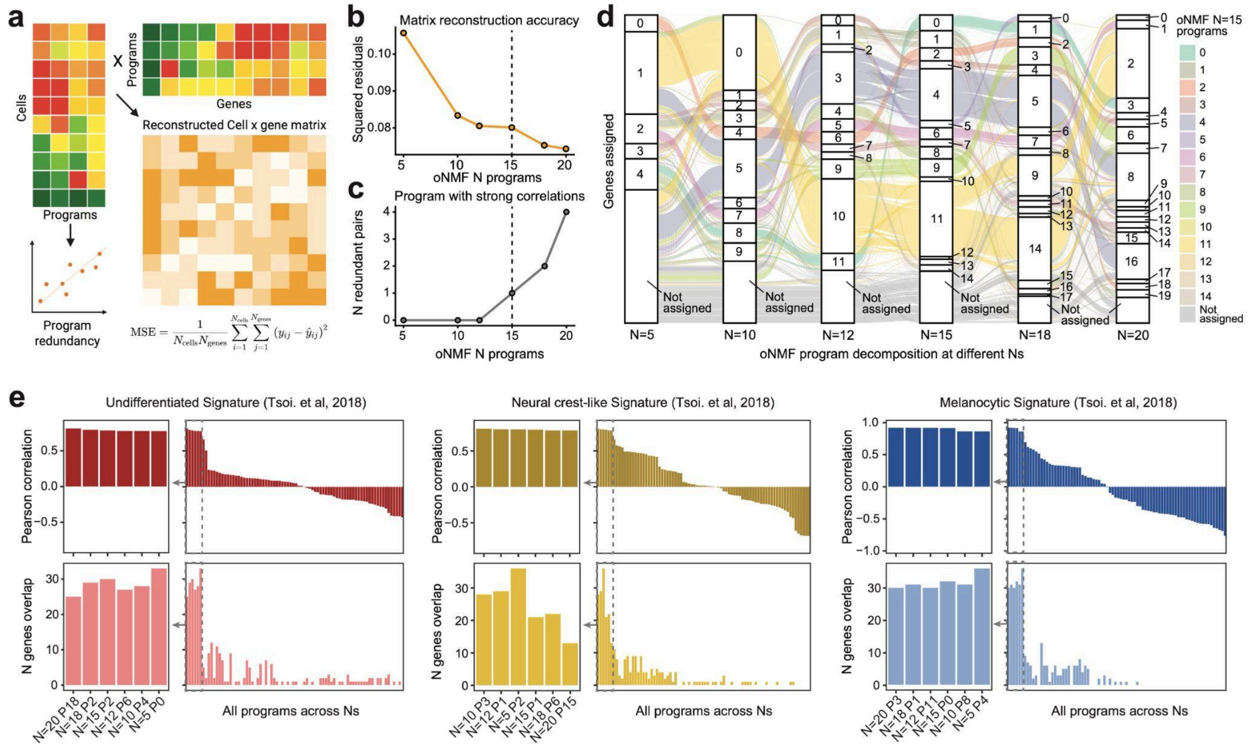

Figure R1-6. Benchmarking the robustness of oNMF program decomposition across different numbers of programs.

a, Schematic showing evaluation of oNMF decompositions by matrix reconstruction accuracy and program redundancy.

b, Mean squared reconstruction residuals across different oNMF program numbers, showing diminishing improvement after approximately 10 programs; dashed line indicates the selected N=15.

c, Number of redundant program pairs, defined by Pearson correlation >0.65 between program scores across melanoma cells, across different program numbers; dashed line indicates N=15.

d, Sankey diagram showing conservation of gene membership across decompositions, with genes colored by their program identity in the selected N=15 decomposition.

e, Benchmarking of programs from different decompositions against known melanoma phenotypic signatures from Tsoi et al. 2018[23], showing consistent recovery of undifferentiated, neural crest-like, and melanocytic programs.

c. The study exclusively uses cancer cell lines cultured in isolation, which by definition lack the complex tumor microenvironment, including immune cells, fibroblasts, endothelial cells, and stromal components, that influences cancer cell behavior in vivo.

We thank the reviewer for raising this important limitation. We agree, and have revised the Discussion to acknowledge it while noting the complementary value of cell lines for isolating cancer-cell-intrinsic programs and the need for future integration with patient-derived and in vivo models (line 1055-1066):

"*A second limitation is that our study profiles cancer cell lines cultured in isolation, which do not fully recapitulate the complex tumor microenvironment, including immune, stromal, endothelial, and fibroblast components. Therefore, our dataset is best interpreted as a resource for dissecting cancer cell-intrinsic regulatory programs. Nevertheless, cell lines provide a tractable and controlled system that minimizes confounding from variable tumor purity, inter-patient heterogeneity, and tissue composition, factors that can complicate analyses of primary tumors, especially for rare cancer subtypes with limited available specimens. The retention of key phenotypic states and regulatory programs in vitro supports the utility of these models for studying intrinsic cancer cell states*."

Reviewer #2, ECR (Remarks to the Author):

I co-reviewed this manuscript with one of the reviewers who provided the listed reports. This is part of the Nature Communications initiative to facilitate training in peer review and to provide appropriate recognition for Early Career Researchers who co-review manuscripts.

We thank the reviewer for the careful evaluation of our manuscript.

Reviewer #3, expertise in pan-cancer single cell analysis (Remarks to the Author):

In “Single-cell multimodal profiling of pan-cancer cell lines uncovers gene regulatory principles underlying intrinsic cell states and environmental features,” the authors leveraged a newly generated, comprehensive cancer-cell-line atlas to dissect gene-regulatory networks and convergent molecular programs across tumor types. By integrating single-cell chromatin accessibility with matched transcriptomes, they identify gene-regulatory phenotypes that arise from differential activation of transcription-factors. At the pan-cancer level, analysis of cis-regulatory elements and upstream TF motifs reveals an EMT-linked cell state that recurs across lineages, supporting a universal transcriptional dedifferentiation program in epithelial cancers. Among the TFs implicated, CEBPB stands out as a driver of the epithelial phenotype, frequently amplified by copy-number gains.

Focusing on melanoma cell lines, the authors resolve subtype-specific transcriptional modules that separate cutaneous from acral melanoma. The most striking differentiating module was found to be JAK-STAT signaling, which is significantly elevated in cutaneous melanoma.

Crucially, the authors project the single-cell-derived regulatory programs onto bulk RNA-seq datasets from patient tumors. The acral-specific program was found to correlate with an immunosuppressive tumor microenvironment, whereas the cutaneous program aligns with a more active antitumor immune response. In addition, scoring subtype-specific programs in bulk tumors was capable of stratifies patients by response to immune checkpoint blockade, underscoring the translational relevance of the in-vitro cell-line derive transcriptional modules.

Overall, I consider this paper a valuable contribution. By integrating ATAC-seq, single-cell transcriptomics, and bulk RNA-seq, the authors present a thorough and well-grounded analysis. The creation of the EasySci cell-line resource will be a useful asset for the community. Nevertheless, I have identified several points that warrant further clarification regarding the novelty of certain results. I also provide suggestions to help strengthen and clarify the work.

Major comments:

1) Validation of the eight gene-regulatory clusters in independent datasets, such as the cell line cohort generated by Zhu Q et al. Nat Commun, 2023 (PMID: 38071219), will be essential to demonstrate reproducibility across different library-preparation techniques and sequencing platforms. Moreover, it will help highlight the novel insights of this work.

We thank the reviewer for this valuable suggestion. We agree that validating the robustness of our classification across single-cell transcriptomes generated by different library-preparation methods and sequencing platforms is important. To this end, we benchmarked our EasySci-based dataset sequenced by Illumina NovaSeq against an external pan-cancer scRNA-seq dataset from Zhu et al.[25], generated using the DNBelab C4 platform and sequenced on BGISEQ500.

Direct comparison of lineage-specific clusters was limited by differences in cell line composition between the two datasets. The Zhu et al. atlas includes more breast, colon, and head and neck cancer cell lines, whereas our lineage-specific clusters include neuroblastoma, sarcoma, hematopoietic and lymphatic malignancies, skin cancer, and prostate cancer. Nevertheless, several lineage-specific regulators identified in our analysis, including PHOX2B and HAND2 for neuroblastoma[26], AR and FOXA1 for prostate cancer[27], and MITF, SOX10, and PAX3 for skin cancer[28], are well supported by prior studies, underscoring the biological interpretability of these clusters.

We therefore focused our cross-platform validation on the pan-cancer epithelial-mesenchymal states (GRN clusters 6-8), the cross-cancer states directly comparable between the two datasets. We trained a support vector machine classifier with 10-fold cross-validation using scaled expression of genes from our pan-cancer TF regulons to predict GRN cluster identity of single cells. The model achieved more than 99% accuracy within our dataset and showed strong predictive performance in the external Zhu et al. dataset as well. Among the four overlapping cell lines, 786-O (renal cell carcinoma), SK-BR-3 (HER2+ breast cancer), MDA-MB-231 (triple-negative breast cancer), and HT29 (colorectal adenocarcinoma), all were assigned to the expected GRN-defined phenotypic states: SK-BR-3 and HT29 to GRN cluster 6 (epithelial), and MDA-MB-231 and 786-O to GRN cluster 8 (mesenchymal) (**Fig. R3-1a, Fig. 2b**). Importantly, these cell lines span distinct tissue origins, indicating that these GRN-defined phenotypes generalize across cancer types rather than reflecting tissue- or platform-specific effects.

We further compared our predictions with the phenotypic annotations reported by Zhu et al. for breast cancer cell lines (**Fig. 1f** in[25]). Consistent with their predefined signature-based marker gene visualization, epithelial-like lines (e.g., MCF7, BT-474, MDA-MB-453) were assigned to our GRN cluster 6, whereas stromal/EMT-like lines (e.g., BT-549, MDA-MB-231, Hs578T) were consistently assigned to our GRN cluster 8 (**Fig. R3-1b**).

Together, these results support the robustness and cross-platform reproducibility of our GRN-based phenotypes and highlight their generalizability across cancer types. Notably, beyond reproducing these cell states, our analysis defines the underlying gene-regulatory programs, including the EMT-associated TF regulons and CEBPB; the cross-platform concordance shows these regulatory phenotypes, not only the transcriptional states, are reproducible. They also suggest that integrating pan-cancer datasets from diverse sources will be valuable for expanding coverage and refining shared regulatory principles across malignancies. We have included these results in our main text (line 258-265).

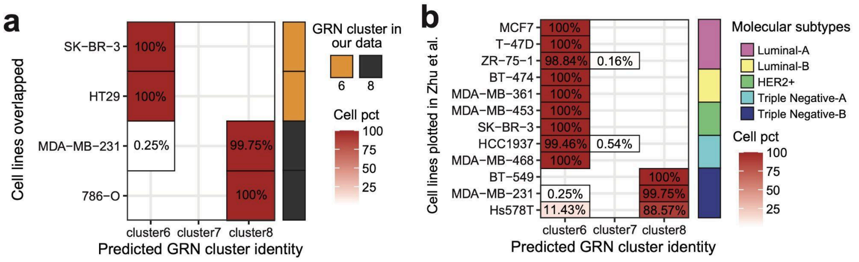

Fig. R3-1. Cross-platform validation of GRN-based phenotypes in an external pan-cancer scRNA-seq dataset.
a, Predicted GRN cluster identities of four overlapping cell lines between our dataset and Zhu et al., showing assignment to the expected epithelial cluster 6 or mesenchymal cluster 8.
b, Predicted GRN cluster identities of breast cancer cell lines from Zhu et al., showing concordance between epithelial/stromal-EMT phenotypes and assignment to GRN cluster 6 or 8.

2) The manuscript lists TYR and PAX3 for skin melanoma, ASPH and CAPN2 for lung carcinoma, STARD3 for breast cancer, RHEB for prostate cancer, and TLE4 for neuroblastoma. However, several of listed genes are not strong, exclusive tissue-specific markers on their own. The expression of some of these genes is either relatively broad or context-dependent, and they are not routinely used as “representative marker genes” for the indicated tissues.

We thank the reviewer for this helpful comment, and we agree that several of the original markers were not optimal lineage-specific choices. We therefore replaced the less specific markers, ASPH and CAPN2 for lung, RHEB for prostate, and TLE4 for neuroblastoma, with well-established lineage markers, while retaining TYR and PAX3 for melanoma and STARD3 for breast cancer with explicit functional justification. We added functional explanations for all of these genes in the main text and updated the marker gene heatmaps accordingly (line 110-117, **Fig. 1e**):

“...such as *TYR* and *PAX3* in skin melanoma cells, which support melanogenesis and melanocytic/neural crest lineage identity; *NKX2-1* and *SFTPB* in lung carcinoma cells, which define lung epithelial identity and surfactant production; *STARD3* and *ERBB2* in HER2-positive breast cancer cells, which mark the HER2-amplified locus and HER2 signaling; *KLK3* and *AR* in prostate cancer cells, which encode prostate-specific antigen and the androgen receptor transcription factor, respectively; and *PHOX2B* and *HAND2* in neuroblastoma cells, which define sympathetic neuronal lineage identity."

3) The authors write: “Conversely, GRHL2, CEBPB, and FOXC1 emerged as putative EMT suppressors: their expression and activity peaked in the epithelial state, and their regulons were negatively associated with EMT progression, extending previous observations from specific cancer types.” The statement should be refined for accuracy and to provide clearer contextualization of the results’ novelty. For example, GRHL2 has already been reported to inhibit EMT in breast cancer (PMID 22379025) and lung cancer (PMID 37600329); these two cancer types are the ones that predominantly populate clusters 6-8 in our analysis. More

generally, the Discussion should more clearly delineate which aspects of the study are novel versus extensions of, and support for, existing literature.

We thank the reviewer for this constructive suggestion, and we agree that the original wording did not cleanly separate established roles from our novel contributions. GRHL2 and FOXC1 are established EMT suppressors, and their reidentification by our unbiased GRN analysis is expected and serves as a positive control validating the approach. Beyond these, our analysis newly implicates CEBPB as a copy-number-amplified epithelial-state regulator, and demonstrates that these EMT-regulatory programs converge across diverse cancer lineages, the principal new contributions of this work. This CEBPB role is supported by orthogonal analyses across distinct assays and cell lines, including ChIP-seq in MCF7, HeLa, A549, HepG2, and HCT116 cells, and CRISPRa perturbation in RPE1 cells.

We also agree that epithelial cancers, particularly breast and lung (8 and 9 cell lines, respectively), are well-represented in clusters 6–8. Importantly, the convergence extends beyond these lineages: clusters 6–8 also include melanoma (SK-MEL-3), renal cell carcinoma (786-O), sarcoma (U2-OS), colorectal cancer (HT-29), and head and neck cancer (MSK921), indicating that the shared regulatory logic is not restricted to epithelial cancers.

To better acknowledge previous studies and clarify the distinction between prior knowledge and the contribution of our present analysis, we have revised the main text as follows (lines 250–255):

"*Conversely, GRHL2, CEBPB, and FOXC1 showed epithelial-state-enriched expression and activity, with regulon activities negatively associated with EMT progression. These findings are consistent with previous studies in specific cancer models implicating GRHL2 in EMT suppression in breast, lung, and ovarian cancer, as well as CEBPB and FOXC1 in breast cancer*[29–31]*, and our analysis extends these cancer-type-specific observations to a broader pan-cancer context*".

We have also revised the Discussion to more clearly delineate the novelty and contextual contribution of this analysis (lines 998–1003):

"*Conversely, the reidentification of epithelial-state-associated transcription factors, including GRHL2, CEBPB, and FOXC1, further supports the convergence of EMT-associated regulatory programs across cancer types. While these factors have been implicated in EMT regulation in specific cancer contexts*[29–31]*, our pan-cancer single-cell analysis places them within a broader cross-lineage framework, highlighting recurrent epithelial-associated regulatory features shared across diverse cancer cell states*".

4) In the absence of a dedicated normal-cell reference, inferCNV uses the average expression across all cells as a pseudo-reference. In cell-line datasets, where most cells share similar CNV profiles, the assumption regarding the existence of a substantial "baseline" population as a reference is often violated. The authors should therefore provide more detail in the appropriate

Methods section on how the CNV analysis for the cell-line data was performed, including how statistically significant CNVs were determined.

We thank the reviewer for raising this important point. We agree that using CNV-free cells as a baseline is a practical strategy when an appropriate normal reference population is available. However, our cohort contains only a limited number of non-cancer cell lines, and even non-cancer or immortalized cell lines can harbor genomic instability and copy-number alterations[32]. IMR90, a human fibroblast cell line, is the closest available diploid-like reference in our cohort. When using IMR90 as the baseline, we observed extensive reference-driven overcorrection (**Fig. R3-2a**). As indicated by the red and blue arrows, multiple regions showed shared shifts across many cell lines, suggesting that IMR90-specific expression patterns introduced reference-related bias into CNV inference.

We therefore used a reference-free inferCNV strategy, in which the average expression across the diverse panel of cell lines serves as a cohort-wide pseudo-reference. Because our panel spans 60 cell lines across 20 diverse cancer types with largely distinct copy-number landscapes, shared cohort-wide alterations are uncommon, making the cohort-average a robust pseudo-reference and mitigating the baseline concern raised for more homogeneous datasets. This strategy has been widely applied in single-cell RNA-seq analyses to infer aberrant CNV patterns for distinguishing malignant from non-malignant cells in an unsupervised manner[7–9], particularly when a matched normal reference is unavailable. In our cell-line atlas, this approach produced more biologically plausible CNV patterns across cell lines, including a largely flat profile for IMR90 and cell line-specific alterations in cancer cell lines (**Extended Data Fig. 5a**). To further evaluate the effect of reference choice, we computed the residual between CNV profiles inferred using IMR90 as the reference and those inferred using the reference-free strategy. The major residual signals were largely conserved across cell lines and aligned well with the overcorrected regions observed in the IMR90-referenced analysis (**Fig. R3-2b**), supporting the robustness of our current strategy.

In the original manuscript, we evaluated the validity of the inferCNV results using two criteria: the inferred CNV profile of IMR90 as a negative control and the concordance between inferred CNV regions and ATAC-derived chromatin accessibility signals, which provide an orthogonal, DNA-proximal proxy for copy-number changes. IMR90 showed minimal inferred CNV signal, supporting its use as a negative control. For inferred amplifications, we retained regions with substantial overlap with elevated ATAC signals, which also allowed us to exclude a regional amplification pattern that lacked orthogonal support (**Extended Data Fig. 6g-i**). Moreover, ATAC accessibility analysis independently highlighted elevated signals at chr11 and chr20 hotspot regions (**Fig. 2f**), as reflected by the enriched signals around genes located within these regions, such as CEBPB, B4GALT5, and UBE2V1 in the chr20 hotspot and CCND1 and TPCN2 in the chr11 hotspot. This cross-modality evidence further increased our confidence in the inferred CNV patterns.

To directly benchmark the accuracy of our CNV inference, we compared the cell line-specific genome-wide inferCNV profiles with publicly available CNV profiles derived from whole-genome

or whole-exome sequencing. The CNV profiles of 786-M1A, H2030-BrM3, MDA231-BrM2, and PC9 inferred from whole-exome sequencing[10] showed strong concordance with our inferCNV results (**Fig. R3-2c; Extended Data Fig. 5c**), despite differences in laboratory source, profiling technology, and computational method. In addition, chr20 hotspot amplifications inferred by inferCNV in epithelial state cell lines SK-BR-03 (**Fig. R3-2d, Extended Data Fig. 5d**) and HT-29 (**Fig. R3-2e, Extended Data Fig. 5e**) were well recapitulated by independent whole-genome sequencing datasets[11,12].

Together, these analyses support the robustness of our CNV inference strategy. We have added additional details to the Methods section describing the reference-free inferCNV analysis and HMM-based CNV calling procedure (Lines 1259–1271):

"*InferCNV (v1.24.0)[33] was used to call CNVs at the meta-cell level based on RNA raw counts. We adopted a reference-free mode to avoid background overcorrection. Gene coordinate files were generated from the reference genome annotation used for read mapping, and RNA meta-cells were grouped by cell line identity. Genes were ordered by genomic position and normalized expression values were smoothed across neighboring genes within each chromosome to identify broad regional expression shifts relative to the cohort-wide pseudo-reference baseline. Then the hidden Markov model (HMM) of inferCNV, i6 HMM model, produced six CNV states: 0 = complete loss, 0.5 = single-copy loss, 1 = neutral, 1.5 = single-copy gain, 2 = two-copy gain, and 3 = more than two-copy gain. Bayesian post-filtering of HMM-defined CNV regions was performed using a default posterior normal-probability cutoff of 0.5, and genomic regions with CNVs supported by posterior probabilities > 0.5 were retained. Retained CNV events were further evaluated based on regional consistency and orthogonal ATAC/WGS/WES support.*"

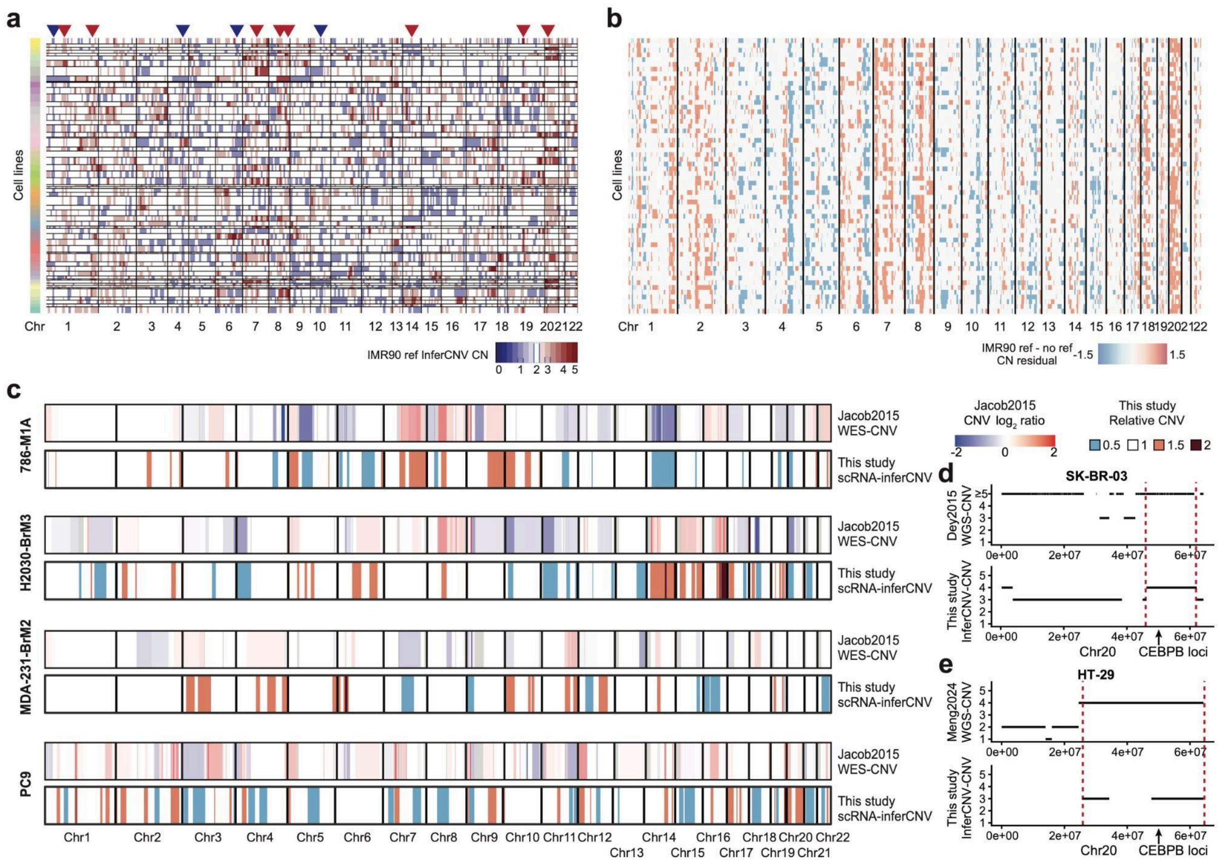

Fig. R3-2. Evaluation and orthogonal validation of scRNA-seq-based CNV inference.

a, Genome-wide inferCNV profiles generated using IMR90 as the reference show broad reference-driven shifts across many cell lines, with representative overcorrected regions highlighted by red and blue arrows.

b, Residual CNV signal between IMR90-referenced and reference-free inferCNV profiles shows conserved differences across cell lines, indicating regions most affected by the choice of reference.

c, Comparison of genome-wide CNV profiles inferred from scRNA-seq in this study with published WES-derived CNV profiles from Jacob et al. for 786-M1A, H2030-BrM3, MDA231-BrM2, and PC9.

d, Comparison of chr20 amplifications in SK-BR-03 between published WGS-derived CNV data and scRNA-seq-based inferCNV from this study.

e, Comparison of chr20 CNV profiles in HT-29 between published WGS-derived CNV data and scRNA-seq-based inferCNV from this study.

5) Scoring the AM and CM signatures on bulk RNA-seq is appropriate, but the results must be interpreted in the context of tumor-cell specificity. The programs' scores could be partially driven by changes in stromal or immune compartment composition rather than by the malignant cells alone. Therefore, the authors should provide evidence that the observed associations between the AM/CM programs and the various cell-type abundances are not merely due to gene expression spill-over from non-cancer fractions.

We thank the reviewer for highlighting the importance of tumor-cell specificity. We agree that tumor microenvironment composition is a potential confounding factor when interpreting gene program scores derived from bulk tumor RNA-seq. To address this concern, we performed additional analyses to evaluate whether the AM- and CM-associated program scores could be explained by non-malignant cell expression or by variation in tumor purity.

First, we examined the cell-type-specific expression of the melanoma subtype-associated gene programs using patient melanoma single-cell RNA-seq data[34]. As shown in **Fig. R3-3a (Extended Data Fig. 8a)**, the average normalized expression of Program 11 and Program 4, representing acral melanoma- and cutaneous melanoma-associated features, respectively, was highly enriched in melanoma tumor cells compared with non-malignant compartments. Program 11 showed approximately 3-fold higher expression in melanoma cells than in non-tumor cells, while Program 4 showed approximately 4-fold higher expression in melanoma cells than in non-tumor cells. In contrast, the expression levels of these program genes were relatively similar across different TME cell types, suggesting that their baseline expression in non-malignant compartments is substantially lower than in melanoma cells.

Second, we assessed whether global tumor purity could account for the bulk program scores in TCGA-SKCM samples. We used previously reported consensus tumor purity estimates derived from multiple tumor purity inference algorithms[35]. The median estimated tumor purity across TCGA-SKCM samples was approximately 80% (**Fig. R3-3b, Extended Data Fig. 8b**), supporting that the bulk transcriptomic profiles are largely dominated by the melanoma compartment. Consistently, both program 11 and program 4 scores were positively associated with estimated tumor purity (**Fig. R3-3c, Extended Data Fig. 8c**), further supporting that these programs primarily capture melanoma-cell-enriched transcriptional signals rather than being driven by non-malignant cell fractions. Overall, given the relatively high tumor purity of TCGA-SKCM samples and the substantially lower expression of these melanoma gene programs in non-malignant cells, it is unlikely that the bulk program scores are primarily driven by a specific TME compartment.

Third, we directly evaluated whether expression of these programs in specific TME compartments could explain the observed subtype-associated bulk differences. Using subtype-resolved patient melanoma single-cell RNA-seq data[36], we scored program 11 across robustly represented non-malignant cell types (≥8 cells per patient). Across these TME cell types, we did not observe a significant AM-specific increase in Program 11 scores (**Fig. R3-3d, Extended Data Fig. 8d**). In specific cell types, such as fibroblasts and pericytes, Program 11 showed a non-significant decrease in AM relative to CM, which is opposite to what would be expected if non-malignant expression were inflating the higher bulk Program 11 score in AM samples.

Together, these analyses indicate that the bulk AM/CM program scores and their associations with deconvolved TME cell-type abundances primarily reflect melanoma-cell-enriched expression rather than spill-over from non-malignant fractions. We have added these analyses

to the main text (line 778-787):

"*To evaluate potential confounding of bulk melanoma subtype-associated program scores by tumor purity or TME composition, we performed additional analyses using patient melanoma single-cell RNA-seq data and the TCGA-SKCM cohort. Both Programs 4 and 11 were strongly enriched in melanoma cells relative to non-malignant TME compartments. TCGA-SKCM samples generally showed high tumor purity, and the bulk scores of these cancer-intrinsic programs were positively associated with estimated tumor purity. Moreover, residual Program 11 gene scores showed no change in TME cell types between AM and CM patients, supporting the interpretation of Program 11 as a melanoma-cell-enriched transcriptional program*."

We acknowledge that computational inference alone cannot fully separate malignant-cell-intrinsic from microenvironment-derived signals, and we have therefore highlighted this caveat in the Discussion (line 1063-1066):

"*Although our analyses suggest that the AM- and CM-associated programs primarily reflect melanoma-cell-enriched transcriptional states, variation in tumor purity and TME composition may still contribute to these scorings. Future integration with subtype-resolved single-cell or spatial transcriptomic datasets will help further distinguish malignant-cell-intrinsic programs from microenvironment-associated components*."

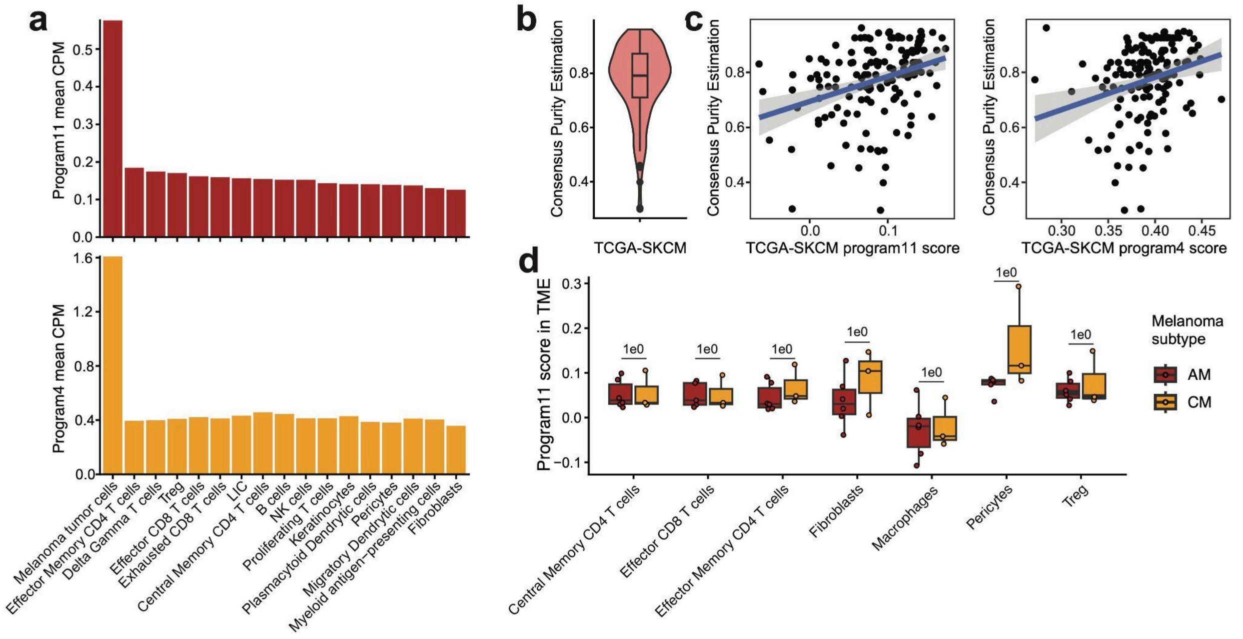

Fig. R3-3. Evaluation of tumor-cell specificity and TME spill-over for melanoma subtype-associated programs.

a, Mean CPM expression of program 11 and program 4 genes across melanoma tumor cells and non-malignant TME cell types in patient melanoma single-cell RNA-seq data.

b, Distribution of consensus tumor purity estimates across TCGA-SKCM bulk RNA-seq samples.

c, Association between consensus tumor purity estimates and bulk program 11 or program 4 scores in TCGA-SKCM samples.
d, Comparison of program 11 scores in non-malignant TME cell types between AM and CM patient single-cell RNA-seq samples.

6) On a similar note, the TCGA-SKCM cohort is dominated by classic sun-exposed cutaneous melanomas; acral melanomas, if present at all, make up only a very small minority (SKCM = Skin Cutaneous Melanoma). Consequently, it is unclear what the AM score captures when applied to this dataset. In Fig 4d the authors present another bulk-RNA-seq cohort but only signaling activities that show significant subtype differences are displayed. To assess the specificity of the entire programs and to confirm the robustness of the scoring strategy, the manuscript should show the distribution of both the AM and CM scores across the relevant melanoma subtypes (i.e., cutaneous vs. acral). This will clarify how the scores behave in patients' tumors of different origins, as one might expect the AM score to be elevated in acral melanomas and the CM score to be higher in cutaneous melanomas.

We thank the reviewer for this careful point about cohort composition and subtype interpretation. We would like to clarify how the TCGA-SKCM cohort can be interpreted given the available clinical annotations. Acral melanoma arises from melanocytes in acral skin sites, including the palms, soles, nail apparatus, fingers, and toes[37]. It is therefore anatomically cutaneous, but represents a distinct site-associated melanoma subtype with etiologic and molecular features that differ from many sun-exposed cutaneous melanomas.

To directly address this request, we examined the distribution of both program scores in an independent melanoma cohort with available subtype annotations[36]. The two programs separated the subtypes in the expected directions: the AM-universal program (Program 11) scored higher in acral melanoma and the CM-universal program (Program 4) scored higher in cutaneous melanoma ($P = 0.048$ and $P = 0.019$, respectively; **Fig. R3-4b**). Importantly, this subtype-resolved behavior is concordant with multiple independent lines of evidence that the AM score reflects genuine acral-associated biology: Program 11 was defined directly from acral melanoma cell lines (**Extended Data Fig. 7g**); its attenuated JAK-STAT and inflammatory activity distinguishes acral from cutaneous melanoma in both cell lines and patient tumors (**Fig. 4c-d**); and higher Program 11 activity is specifically associated with an immunosuppressive tumor microenvironment in patient tumors (**Fig. 5b**). Together, these convergent results indicate that the AM and CM scores capture subtype-associated melanoma cell states. We have included the analysis in the manuscript (**Extended Data Fig. 7i**).

Turning to the TCGA-SKCM cohort raised by the reviewer, we curated the available clinical metadata before performing the analysis, but did not find definitive subtype-level annotations that would allow us to reliably classify individual tumors as acral versus non-acral cutaneous melanoma. The recorded tissue or organ of origin was skin, and the primary diagnosis was malignant melanoma, but detailed anatomical site of origin and clinical subtype information were not consistently available. Thus, while TCGA-SKCM may include acral melanoma cases, we cannot confidently separate them from other cutaneous melanoma cases based on the

available metadata.

Accordingly, we did not use TCGA-SKCM as a definitive acral-versus-cutaneous validation cohort. Instead, we used it to ask whether the melanoma programs identified in our cell-line atlas are detectable in bulk melanoma tumors and whether these tumor-intrinsic program scores are associated with tumor microenvironmental features. This analysis is informative because the programs are interpreted as melanoma cell-state features rather than strict clinical subtype labels. We hypothesize that tumors acquiring an acral-like or cutaneous-like transcriptional state may show corresponding differences in tumor ecosystem features, even when precise clinical subtype annotation is unavailable.

We further used the available sampling-site metadata in TCGA-SKCM as an exploratory proxy, while recognizing its limitations. Although sampling sites alone cannot establish melanoma subtype, samples collected from extremity sites showed higher Program 11 scores than head and neck samples (**Fig. R3-4a**), which are more likely to represent sun-exposed cutaneous melanoma. In addition, one recently reported acral melanoma case in TCGA-SKCM, TCGA-ER-A19T[38], was recorded as an extremity sample in the metadata, supporting the plausibility that extremity-enriched Program 11 activity may capture acral-associated biology. This interpretation was concordant with the specific association between Program 11 and immunosuppressive TME features in TCGA-SKCM (**Fig. 5b**).

Together, these results support our interpretation that Program 11 and Program 4 capture subtype-associated melanoma cell states, while the TCGA-SKCM analysis evaluates the broader tumor and microenvironmental relevance of these states rather than definitively classifying TCGA samples by melanoma subtype.

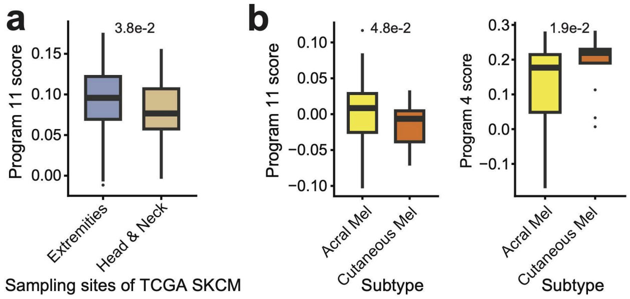

Fig. R3-4. Validation of melanoma subtype-associated programs in TCGA-SKCM and an external melanoma cohort.
a. Program 11 scores in TCGA-SKCM extremity versus head and neck samples.
b. Program 11 and Program 4 scores in acral versus cutaneous melanoma from an external cohort.

7) Regarding the immunotherapy-cohort analyses (i) The study cited as reference 71 (PMID 31792460) includes a larger patient cohort than the one used in the present manuscript. The authors should clarify why a smaller subset was selected and provide a rationale for this sampling strategy. (ii). Leveraging additional publicly available datasets (e.g., PMID 37037616,

PMID 26359337, PMID 30753825, etc.) and conducting multivariate analyses would enable assessment of whether the associations between individual gene-signature scores and immunotherapy response remain independent of one another, and of other known clinical covariates, thereby providing a clearer picture of the distinct predictive value of the signatures identified by the authors.

We thank the reviewer for these constructive suggestions. We agree that the cohort selection strategy should be more clearly described, and that additional validation and multivariable analyses are important for evaluating the robustness and context-dependence of signatures.

First, we would like to clarify that, because access to unprocessed raw data from multiple patient cohorts[39,40] is restricted, our original immunotherapy analyses were performed through Cancer Immunology Data Engine (CIDE)[41], a recently published resource that collected and processed bulk RNA-seq profiles and clinical metadata across multiple cancer immunotherapy cohorts. For Liu2019[39], CIDE stratifies anti-PD-1-treated melanoma patients according to prior ipilimumab exposure, a CTLA4 inhibitor, and biopsy timing. Based on these annotations, patients were mainly separated into an ipilimumab-naive subgroup (n = 74), an ipilimumab-pretreated subgroup (n = 46) (**Fig. R3-5a**). Thus, the smaller cohort used in our original analysis corresponded to the ipilimumab-pretreated anti-PD-1 subgroup.

We focused on this subgroup because our original analysis suggested that the GRN signature stratified outcome specifically in patients receiving anti-PD-1 therapy after prior anti-CTLA-4 treatment. To test whether this association also applied to ipilimumab-naive patients, we applied the same GRN signature to the Liu2019 ipilimumab-naive subgroup. In this setting, the signature did not significantly stratify either progression-free survival (PFS; **Fig. R3-5b**) or overall survival (OS; **Fig. R3-5c**). Importantly, this weaker stratification was not unique to our GRN signature: even immuno-predictive score (IMPRES)[42], the top-ranked immune-response signature in this analysis, did not reach statistical significance for either PFS or OS (PFS, p=1.11e-1; OS, p=1.22e-1). These results suggest that transcriptomic stratification of anti-PD-1 outcome may be generally more challenging in the anti-CTLA-4-naive setting. A possible interpretation for this context dependence is that baseline tumor-immune states may be more heterogeneous before anti-CTLA-4 exposure, whereas prior anti-CTLA-4 treatment may reshape and reveal more differences between immunologically responsive and suppressive tumor microenvironments[43].

To further evaluate generalizability, we analyzed an independent ipilimumab-pretreated anti-PD-1 melanoma cohort from Riaz2017[44] using the same CIDE framework. Although the GRN signature did not show the strongest association with PFS, it ranked highest for OS prediction among the evaluated signatures (**Fig. R3-5d,e**). These results provide additional support for the relevance of the GRN signature in the post-anti-CTLA-4 anti-PD-1 setting, despite the effects of intercohort heterogeneity and sample size.

In response to the reviewer's suggestion, we further expanded our analysis to the Campbell2023 melanoma immunotherapy resource (PMID 26359337)[43], which integrates

multiple published cohorts, including Gide2019 (PMID 30753825)[45]. We separately analyzed patients with prior anti-CTLA-4 treatment (n = 102) and without prior anti-CTLA-4 treatment (n = 335). Using log-transformed CPM RNA-seq profiles collected in the resource, we computed the GRN signature score and performed Cox proportional-hazards regression for PFS and OS. Consistent with the CIDE-based analyses, the GRN signature significantly stratified anti-CTLA-4-pretreated patients for both PFS and OS, with higher GRN scores associated with improved outcome (**Fig. R3-5f**). In contrast, the same signature did not significantly stratify outcomes in anti-CTLA-4-naive patients (**Fig. R3-5g**).

We next examined whether the GRN signature carries predictive value independent of the other signatures and of clinical covariates. Because published immune-response signatures share substantial gene overlap and are strongly collinear, entering them jointly in a single survival model yields unstable estimates; we therefore assessed their relative, mutually independent contributions through the predictive-power ranking analysis (**Fig. R3-5b-e**), in which the GRN signature consistently ranked among the top performers in the post-anti-CTLA-4 setting. To test independence from clinical covariates, we performed multivariable CoxPH analyses in the anti-CTLA-4-pretreated Campbell2023 resource, including the GRN signature score together with age and sex as covariates. For PFS, the GRN signature retained a protective direction but did not reach a strong statistical significance after covariate adjustment (**Fig. R3-5h**). For OS, the GRN signature was significantly associated with improved outcome after adjustment, while older age was associated with worse outcome (**Fig. R3-5i**). Male sex also showed a protective association in this selected cohort, consistent with previous reports of gender-associated differences in melanoma immunotherapy outcomes[46], although we interpret this observation cautiously given the complexity of sex effects.

Together, across independent datasets, the GRN signature showed a consistent association with outcome in melanoma patients receiving anti-PD-1 therapy after prior anti-CTLA-4 exposure, whereas its predictive value was absent in anti-CTLA-4-naive patients. We have revised the manuscript to explicitly describe the patient subgroup definitions, added the expanded cohort and analyses, and clarified that the predictive value of this GRN signature appears context-dependent (line 836-859).

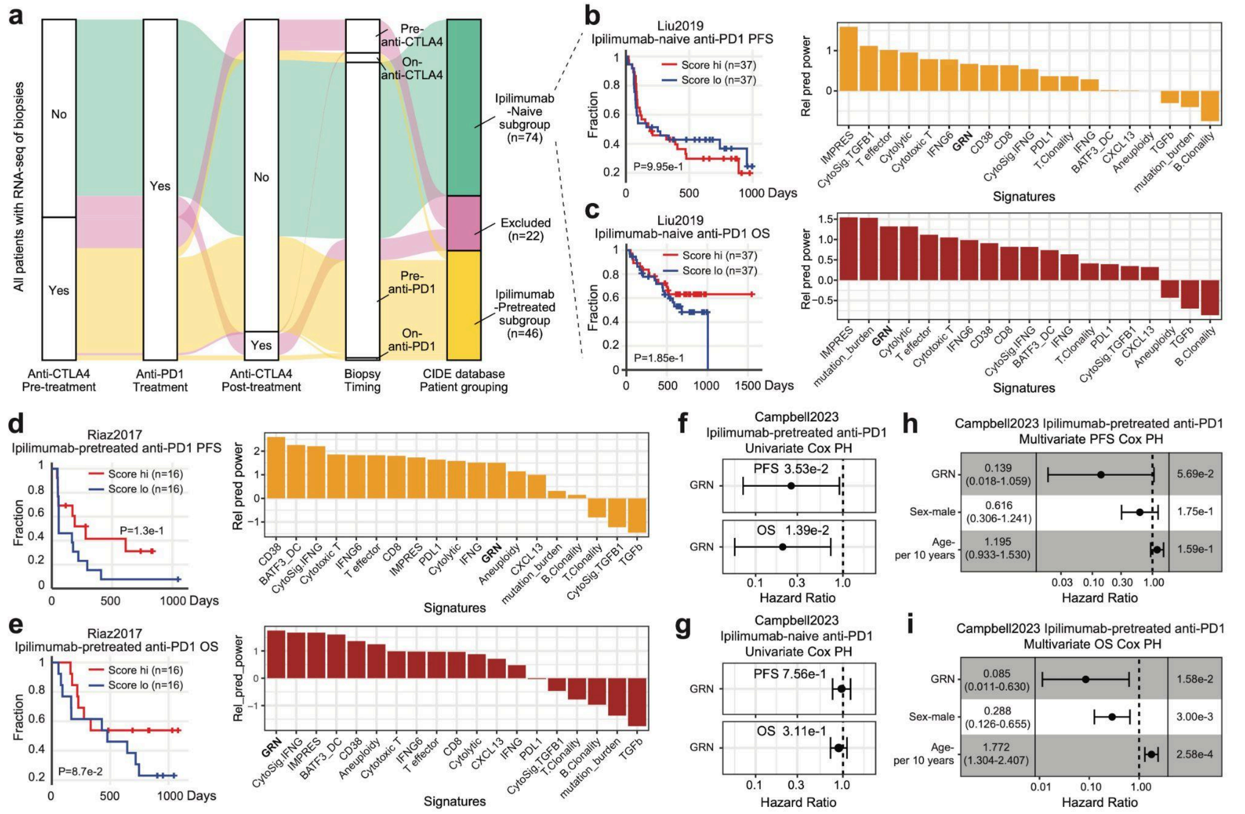

Fig. R3-5. Evaluation of the GRN signature in anti-PD-1-treated melanoma cohorts stratified by prior anti-CTLA-4 exposure.

a, Sankey diagram showing classification of Liu2019 anti-PD-1-treated melanoma patients by prior anti-CTLA-4 treatment, post-treatment anti-CTLA-4 exposure, biopsy timing.

b, Kaplan-Meier analysis and relative predictive-power ranking of the GRN signature and published immunotherapy signatures for PFS in the Liu2019 ipilimumab-naive anti-PD-1 subgroup.

c, Kaplan-Meier analysis and relative predictive-power ranking of the GRN signature and published immunotherapy signatures for OS in the Liu2019 ipilimumab-naive anti-PD-1 subgroup.

d, Kaplan-Meier analysis and relative predictive-power ranking of the GRN signature and published immunotherapy signatures for PFS in the Riaz2017 ipilimumab-pretreated anti-PD-1 cohort.

e, Kaplan-Meier analysis and relative predictive-power ranking of the GRN signature and published immunotherapy signatures for OS in the Riaz2017 ipilimumab-pretreated anti-PD-1 cohort.

f, Univariate CoxPH analysis showing the association between the GRN signature and PFS or OS in ipilimumab-pretreated anti-PD-1-treated patients from Campbell2023.

g, Univariate CoxPH analysis showing the association between the GRN signature and PFS or OS in ipilimumab-naive anti-PD-1-treated patients from Campbell2023.

h, Multivariable CoxPH analysis of PFS in ipilimumab-pretreated anti-PD-1-treated Campbell2023 patients, including the GRN signature, sex, and age as covariates.

i, Multivariable CoxPH analysis of OS in ipilimumab-pretreated anti-PD-1-treated Campbell2023 patients, including the GRN signature, sex, and age as covariates.

8) Extended Data Fig. 1 currently displays data for only a subset of the cell lines. Please include all the cell lines in this figure (or in a supplemental table) so that readers can assess the overall data quality and gauge the robustness of results derived from any individual line or from downstream analyses.

We thank the reviewer for this constructive suggestion. To provide a more comprehensive view of the dataset, we have updated **Extended Data Fig. 1** to include quality-control metrics for all profiled cell lines. This allows readers to assess the overall data quality across the full panel and evaluate the robustness of analyses. The processed dataset and associated metadata are also publicly available through GEO under accession number GSE311521.

9) The raw FASTQ files, processed count matrices, and cell-metadata are central to the study and would constitute a valuable resource for the community. However, the current Data-Availability statement does not guarantee that these data will be deposited, and there is no reason for withholding the analysis code during peer review. I recommend that the authors upload the raw and processed data, as well as the code, to appropriate public repositories.

We thank the reviewer for this important suggestion and agree that public availability of the raw data, processed matrices, metadata, and analysis code will be valuable for the community. Since the initial submission, we have deposited the processed gene count matrices and ATAC peak count matrices in GEO under accession number GSE311521. The raw FASTQ files have also been deposited under BioProject accession PRJNA1354039. In addition, we have uploaded key analysis scripts to GitHub: https://github.com/JunyueCaoLab/Cancer_atlas. These accession numbers and repository links have been added to the revised Data Availability statement.

Minor comments:

1) In the first Main paragraph, the authors wrote that “analyses of primary tumors are often confounded by the presence of non-tumor components (e.g., stroma, immune cells, and adjacent normal tissue), making it difficult to define the intrinsic regulatory programs shared across cancer lineages.” This statement is inaccurate if the focus is on “analyses”. Modern snRNA-seq pipelines reliably distinguish malignant cells from non-malignant cells by exploiting copy-number variation profiles, and lineage-specific expression markers. Consequently, cancer-cell intrinsic programs can be inferred despite the presence of stromal and immune compartments. I recommend revising the sentence.

We thank the reviewer for pointing this out. We agree that the original phrasing overstated this point and have revised it accordingly. The masking of malignant-versus-non-malignant heterogeneity is now attributed to bulk profiling, and cell lines are presented as a complementary, controlled system for isolating tumor-intrinsic programs (line 52-56).

2) In the second Main paragraph, the authors state that “most studies rely on droplet-based platforms that are restricted to a single molecular modality or limited cell numbers per experiment, constraining the ability to map chromatin-gene regulatory relationships across diverse cancer states.” The manuscript does not provide evidence that a large number of cells per experiment is required to increase discovery power in this context. In fact, cancer cell lines are considered relatively homogeneous compared with primary tumours or patient-derived samples. For such homogeneous systems, modest cell numbers are usually sufficient to capture the relevant regulatory variation. At a minimum, the authors should re-phrase the statement to acknowledge only the primary limitation. However, providing data that evaluate the association between increasing cell numbers and improved chromatin-gene regulatory mapping would be a better approach and could potentially help contextualize higher cell numbers as a key advantage of the study.

We thank the reviewer for prompting us to substantiate this point. We agree that cancer cell lines are generally more homogeneous than primary tumors or patient-derived samples, so the advantage of increased cell numbers here is not the discovery of rare cell states but improved sensitivity for chromatin accessibility detection and regulatory network inference.

To directly address this, we performed downsampling analyses on our single-cell ATAC data. Using A375 as a representative example, the number of ATAC peaks called increased rapidly at low sampling fractions and approached saturation at higher fractions (**Fig. R3-6a**), indicating that peak detection is sensitive to sampling depth while confirming that our current coverage recovers a near-saturated peak set.

We further subsampled cells and reconstructed gene regulatory networks using SCENIC+. TF-regulator recovery was robust even at lower fractions (**Fig. R3-6b**), whereas regulon target-gene recovery was more sensitive to coverage and improved with more cells (**Fig. R3-6c**), indicating that higher cell numbers improve target-gene assignment in sparse chromatin-based regulatory inference. We have added these analyses to the revised manuscript (**Extended Data Fig. 3**).

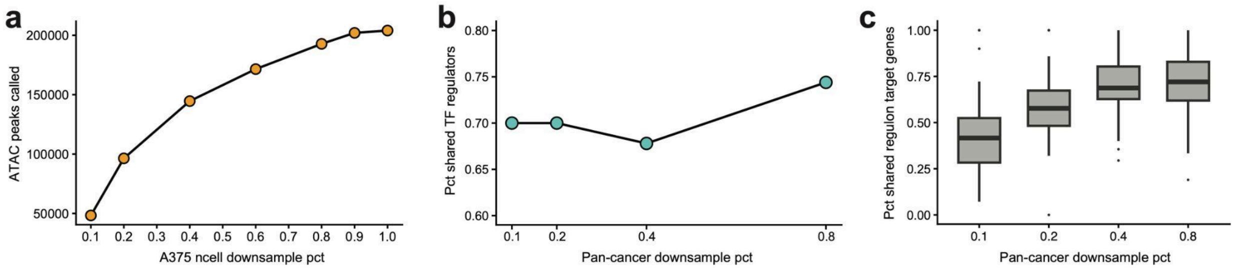

Fig. R3-6. Downsampling analysis evaluating the effect of cell number on chromatin accessibility and gene regulatory network reconstruction.
a, Number of ATAC peaks called from the A375 cell line across increasing cell downsampling fractions.

b, Fraction of TF regulators identified to be shared with the full dataset across pan-cancer downsampling fractions.
c, Fraction of regulon target genes identified to be shared with the full dataset across pan-cancer downsampling fractions.

3) Define "GRN" (gene-regulatory network) at first use.

We have added the definition of gene regulatory network at its first appearance in the manuscript (line 43-48):

"*Gene regulation shapes cellular behavior, state transitions, and lineage development. Across chromatin and RNA layers, transcription factors (TFs) bind enhancers and promoters to modulate chromatin state and tune downstream gene expression. Reconstructing gene regulatory networks (GRNs), which capture the interconnected regulatory architecture through which TFs coordinate gene-expression programs, can therefore provide mechanistic insight into the regulation of cellular phenotypes*."

4) There is a typo linking CEBPB and UBE2V1 to chr11 rather than chr20

We apologize for the typo. We have corrected the chromosome annotation for CEBPB and UBE2V1 in the manuscript.

5) There is a typo the in x-axis of extended data fig 3f ('Backgroumd' instead of 'Background')

We apologize for the typo. We have corrected the x-axis label in the new **Extended Data Fig. 3h**.

6) A citation appears to be missing after the sentence: "We next asked whether amplification of these genomic loci impacts drug response, given that cancer cell state is known to influence drug sensitivity."

We apologize for the oversight. We have added appropriate citations to support the statement that cancer cell state can influence drug sensitivity.

7) Reference number 72 cite the Author Correction rather than the original manuscript.

We apologize for the mistake. We have replaced the Author Correction citation with the original article.

8) Extended Data Fig. 8 a–d lacks information on what the displayed numbers represent (P-values) and does not indicate which statistical test was use.

We apologize for the lack of clarity. We have revised the legend for the new **Extended Data Fig. 10** to indicate that the displayed numbers represent P-values and to specify the statistical test

used.

Reviewer #4, expertise in single cell analysis for acral melanoma (Remarks to the Author):

This study presents a large-scale, multimodal single-cell atlas of 60 human cancer cell lines, leveraging an in-house EasySci platform to generate over 460,000 single-nucleus transcriptomic and chromatin accessibility profiles. By integrating RNA and ATAC data, the authors systematically construct pan-cancer gene regulatory networks (GRNs), delineate conserved epithelial–mesenchymal transition (EMT) states across lineages, and perform an in-depth comparative analysis of acral melanoma (AM) versus cutaneous melanoma (CM). The study further links in vitro regulatory features to tumor microenvironment (TME) composition and immunotherapy responses, demonstrating the translational potential of cancer cell line–derived regulatory signatures.There are several limitations to be revised.

1. Although cell lines enable the isolation of tumor-intrinsic programs,they can not define the TME interactions such as fibroblasts, immune cells,and ECM.

We agree with the reviewer's comment and fully acknowledge this limitation of the current study. The primary scope of our study is to provide a rich resource of chromatin accessibility and transcriptomic landscapes across cancer cell lines from diverse tissue origins. Cancer cell lines offer an isolated and experimentally tractable system for studying cancer-cell-intrinsic mechanisms, and have long served as standard models for genetic engineering, perturbation, drug screening, and mechanistic studies. At the same time, important questions remain regarding how in vitro culture shapes cell-line phenotypes, how well cancer cell lines preserve features of their corresponding tumors *in vivo*, and which models are most appropriate for specific biological questions.

Notably, our focused analysis of melanoma subtypes revealed extensive connections between *in vitro* cell-line states and features of their original tumor contexts, supporting the retention of disease-relevant phenotypes and regulatory networks in cultured models. We therefore view this resource as complementary to patient-sample profiling. While cell lines cannot directly define interactions with fibroblasts, immune cells, extracellular matrix, or other components of the native tumor microenvironment, they provide a controlled framework for dissecting tumor-intrinsic regulatory logic. Integration with patient tumor resources can help extend phenotypic observations toward experimentally testable mechanistic hypotheses. We have now clarified this limitation and scope in the Discussion (lines 1055–1066).

Also the regulatory networks and pathways can not be demonstrated or validations.

We thank the reviewer for this point. The robustness of our pan-cancer gene regulatory network characterization is supported by multiple lines of evidence.

First, many tissue-specific TF regulators identified in our analysis have been well characterized in their corresponding lineage or cancer contexts. For example, PHOX2B and HAND2 in neuroblastoma[26], AR and FOXA1 in prostate cancer[27], and MITF, SOX10, and PAX3 in melanoma/melanocytic lineages[28], are well supported by prior studies, underscoring the

biological interpretability of these clusters.

In cross-cancer-type epithelial-to-mesenchymal states, multiple TF regulators identified in our analysis have also been previously implicated in EMT regulation, including AP-1, TEAD family factors, and ZEB1[47]. Conversely, EMT-suppressive roles for GRHL2, CEBPB, and FOXC1 have been reported in specific cancer models[29–31], further supporting the relevance of our inferred regulators.

Our EMT state characterization, derived from both epigenetic and transcriptomic layers, exhibited strong concordance with transcriptomic states inferred using a conserved EMT signature derived from a distinct spectrum of cell lines in an independent study[17]. This agreement supports the generalizability of our GRN-based state classification.

We further validated CEBPB's binding dynamics across EMT by projecting external cell-line transcriptomic profiles[25] to the EMT trajectory and examining corresponding ENCODE ChIP-seq data (**Fig. 3i-j**). In addition, we supported the causal relevance of CEBPB using CRISPRa Perturb-seq data[48], where experimental activation of CEBPB led to an elevated CEBPB regulon score (**Fig. 3k-l**).

Finally, to validate the robustness of the regulatory phenotypes identified in our dataset, we trained a support vector machine model using the expression of pan-cancer GRN target genes to predict single-cell phenotype labels (**Fig. R4-1, Extended Data Fig. 3k-m**). When applied to an external cancer cell-line single-cell transcriptomic dataset generated by Zhu et al[25], this model successfully recovered pan-cancer EMT states in both overlapping cell lines (**Fig. R4-1a**) and previously unseen cell lines annotated by Zhu et al. (**Fig. 1f** in[25]) as occupying more differentiated epithelial or more mesenchymal states (**Fig. R4-1b**).

Together, these analyses provide multiple layers of support for the biological interpretability and generalizability of the regulatory programs we identified. We agree that systematic validation remains an important future direction, and such experiments would be valuable for further dissecting the candidate mechanisms nominated by our GRN analysis.

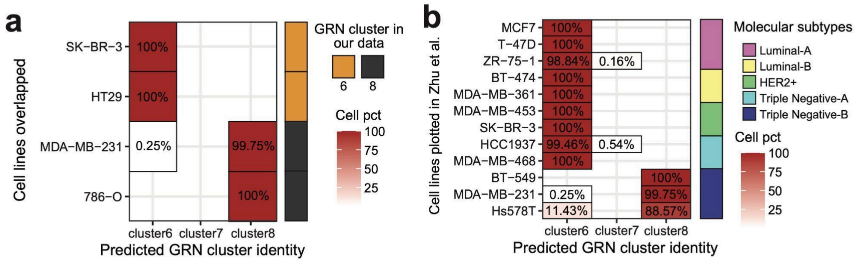

Fig. R4-1. Cross-platform validation of GRN-based phenotypes in an external pan-cancer scRNA-seq dataset.

a, Predicted GRN cluster identities of four overlapping cell lines between our dataset and Zhu et al., showing assignment to the expected epithelial cluster 6 or mesenchymal cluster 8.

b, Predicted GRN cluster identities of breast cancer cell lines from Zhu et al., showing

concordance between epithelial/stromal-EMT phenotypes and assignment to GRN cluster 6 or 8.

2. The data lack spatial transcriptomics or imaging-based validation.Without spatial data and evaluation, it is difficult to ascertain whether ligand–receptor pairs are expressed in physically proximate cells.

We thank the reviewer for this well-taken point. We agree that spatial transcriptomics or imaging-based validation would be required to directly confirm physical proximity between ligand- and receptor-expressing cells. In the current study, however, our primary goal was to establish a pan-cancer cultured cell-line atlas and use cancer cell-intrinsic regulatory programs to infer their potential retention of in vivo tumor features. Accordingly, the ligand-receptor and TME analyses were intended as complementary, hypothesis-generating analyses rather than direct evidence of physical cell-cell interactions.

To strengthen the biological relevance of these observations, we integrated multiple patient-derived resources, including TCGA bulk tumor analyses and published single-cell RNA-seq data from melanoma patient samples. These analyses provided convergent support for TME differences associated with acral and cutaneous melanoma programs, while we acknowledge that they do not replace spatial validation. We also explored available spatial transcriptomic resources for acral melanoma. Although a limited number of studies[34,49] reported spatial data from patient samples, which also supports the immunosuppression landscape of acral melanoma TME, access to the relevant datasets was restricted, and we were unable to obtain approval for data use before submission of this revision. We have therefore revised the manuscript to clarify the interpretive scope of the ligand–receptor analysis and to state that spatial transcriptomic or imaging-based validation will be an important direction for future studies (line 1072-1076):

"*Future integration with subtype-resolved single-cell, spatial transcriptomic, or imaging-based datasets will help further distinguish malignant-cell-intrinsic programs from microenvironment-associated components and strengthen mechanistic understanding of how specific TME phenotypes are established and maintained.*"

3. Copy number variations were inferred from RNA expression using InferCNV. It has variable accuracy. Should chose whole-genome sequencing (WGS) or single-cell DNA-seq.

We thank the reviewer for raising this methodological point. We agree that CNV inference from single-cell RNA-seq has lower resolution and potentially variable accuracy compared with direct DNA-based measurements such as WGS, WES, or single-cell DNA-seq. However, these DNA-based approaches remain difficult to apply comprehensively across large-scale single-cell cancer cell-line panels because of throughput and sequencing requirements. In contrast, CNV inference from single-cell RNA-seq or ATAC-seq has been widely used to estimate broad copy-number alterations of cancer cells[4–6], and to distinguish malignant from non-malignant cells based on CNV patterns[7–9] in an unsupervised manner. In response to the comment, we have

validated these inferCNV results through several orthogonal approaches (also described in detail in our response to Reviewer #3 (point 4) and in the manuscript)

In the original manuscript, we have evaluated the reliability of our inferCNV results using two orthogonal criteria: the inferred CNV profile of IMR90 as a diploid negative control, and the concordance between inferred CNV regions and ATAC-derived chromatin accessibility signals. IMR90 showed minimal inferred CNV signal (**Extended Data Fig. 5a**), supporting the reliability of the baseline. For inferred amplification events, we retained two regions with substantial overlap with elevated ATAC signals and excluded a regional amplification pattern lacking such orthogonal support (**Extended Data Fig. 6g-i**). ATAC accessibility analysis also independently highlighted elevated signals at chr11 and chr20 hotspot regions (**Fig. 2f**), as reflected by enriched signals around genes located within these regions, such as CEBPB, B4GALT5, and UBE2V1 in the chr20 hotspot and CCND1 and TPCN2 in the chr11 hotspot.

To further benchmark our CNV inference, we compared cell line-specific genome-wide inferCNV profiles with publicly available CNV profiles derived from WGS or WES. CNV profiles of 786-M1A, H2030-BrM3, MDA231-BrM2, and PC9 from WES[10] showed strong concordance with our inferCNV results (**Fig. R4-2a**), despite extensive differences in laboratory source, profiling technology, and computational method. In addition, chr20 hotspot amplifications inferred by inferCNV in the epithelial-state cell lines SK-BR-03 and HT-29 were recapitulated by independent WGS datasets[11,12] (**Fig. R4-2b-c**).

Together, these analyses support the robustness of our CNV inference strategy for identifying broad copy-number patterns within our single-cell multimodal resource. We agree that direct DNA-based profiling would provide higher-resolution validation and remains an important future direction; accordingly, we have included these additional benchmarking analyses in the revised manuscript (**Extended Data Fig. 5**).

[REDACTED]

Fig. R4-2. Validation of scRNA-seq-based CNV inference.
a, Comparison of genome-wide CNV profiles inferred from scRNA-seq in this study with published WES-derived CNV profiles from Jacob et al. for 786-M1A, H2030-BrM3,

MDA231-BrM2, and PC9.
b, Comparison of chr20 amplifications in SK-BR-03 between published WGS-derived CNV data and scRNA-seq-based inferCNV from this study.
c, Comparison of chr20 CNV profiles in HT-29 between published WGS-derived CNV data and scRNA-seq-based inferCNV from this study.

4. No validation for almost all the data. Make an example, CEBPB was proposed as an epithelial-state gate keeper based on in silico knockout. Please chose experimental perturbation methods to validate.

We thank the reviewer for this comment and fully agree that functional validation is critical for supporting the proposed role of CEBPB as an epithelial-state regulator. that functional validation is critical for supporting the proposed role of CEBPB as an epithelial-state regulator. We would like to clarify that CEBPB was not nominated solely based on in silico knockout, but through multiple concordant lines of evidence linking copy-number amplification, CEBPB regulatory activity, and epithelial-state maintenance at the pan-cancer level. This observation is also consistent with previous reports identifying CEBPB as an EMT repressor in specific cancer models.

To further test the generalizability of this conclusion to the pan-cancer context, we incorporated both external chromatin-binding data and experimental perturbation datasets. First, using publicly available transcriptomic profiles, we projected external cancer cell lines not included in our dataset onto the EMT trajectory and analyzed ENCODE CEBPB ChIP-seq data from these lines (**Fig. 3i–j**). This analysis showed progressive loss of CEBPB binding along EMT at predicted epithelial target loci, including regulatory regions near KRT8 and KRT18 identified in our study, supporting the association between CEBPB chromatin occupancy and epithelial gene regulation.

Second, we analyzed published large-scale CRISPRa[48] and CRISPRi[50] Perturb-seq datasets to evaluate the functional impact of CEBPB perturbation. In the original manuscript, we showed that CRISPRa-mediated activation of CEBPB in RPE1 cells[48] significantly increased the CEBPB regulon score (**Fig. 3k, Fig. R4-3a**) and induced canonical epithelial identity markers predicted as CEBPB targets in our pan-cancer GRN (**Fig. 3l, Fig. R4-3b**). During revision, we further analyzed an independent CRISPRi Perturb-seq dataset generated in HEK293 cells[50] and found that CEBPB knockdown significantly reduced the CEBPB regulon score (**Fig. R4-3c**). Consistently, these canonical epithelial genes were also downregulated upon CEBPB knockdown (**Fig. R4-3d**), opposite to the trend observed after CRISPRa-mediated activation.

Together, these analyses provide additional experimental support for CEBPB as an epithelial-state regulator across cancer contexts.

[REDACTED]

Fig. R4-3. CRISPRa and CRISPRi Perturb-seq analyses support the regulatory role of CEBPB in epithelial-state maintenance.
a, Box plot showing CEBPB regulon scores in NTC and CEBPB-targeting cells from the CRISPRa Perturb-seq dataset of Southard et al. P-value was calculated using a two-sided Wilcoxon test.
b, Dot plot showing expression of CEBPB and representative epithelial genes predicted as CEBPB targets in NTC and CEBPB-targeting cells from the CRISPRa Perturb-seq dataset.
c, Box plot showing CEBPB regulon scores in NTC and CEBPB-targeting cells from the CRISPRi Perturb-seq dataset of Huang et al. P-value was calculated using a two-sided Wilcoxon test.
d, Dot plot showing expression of CEBPB and representative epithelial genes predicted as CEBPB targets in NTC and CEBPB-targeting cells from the CRISPRi Perturb-seq dataset.

5. The single-cell TME data were derived from a single published study (GSE215121). This dataset includes both AM and CM patients,but the ethnicities and treatment backgrounds are unknown.

We thank the reviewer for this point regarding the patient TME data source. Due to the rarity of acral melanoma, single-cell profiling datasets of patient tumors remain largely limited. This limitation was part of our motivation for using acral melanoma-derived cell lines as a controlled and scalable resource, while using patient-derived datasets as complementary validation.

The Zhang et al. dataset (GSE215121)[36] remains, to our knowledge, one of the most comprehensive single-cell resources for acral melanoma patient tumors with TME information. We therefore reprocessed and annotated this dataset to complement the TME associations inferred from TCGA-SKCM bulk deconvolution.

To address the reviewer’s concern, we retrieved available ethnicity and clinical information from the original study and summarized these annotations in **Table R4-1**. In the original analyses, to minimize potential confounding from treatment effects, we already excluded cells from post-treatment samples or metastasis from the analysis.

We agree that larger single-cell acral melanoma cohorts with more complete clinical annotations will be important for further evaluating how ethnicity, treatment history, and other clinical variables influence TME composition and cell states. We have clarified this limitation in the revised manuscript (line 1068-1076):

"*In addition, ligand-receptor inference should be interpreted as an exploratory analysis for hypothesis nomination. Our patient-level tumor-microenvironment inferences also relied primarily on a single acral melanoma single-cell cohort with limited clinical annotation, and larger, more completely annotated cohorts will be needed to assess how ethnicity, treatment history, and other clinical variables shape TME composition and cell states. Future integration with subtype-resolved single-cell, spatial transcriptomic, or imaging-based datasets will help further distinguish malignant-cell-intrinsic programs from microenvironment-associated components and strengthen mechanistic understanding of how specific TME phenotypes are established and maintained*."

[REDACTED]

Table R4-1. Clinical meta-data of patient samples used for TME single-cell RNA analysis in this study, obtained from the original study (Zhang et al.[36]).

6. The main problem is almost all the results based on cell lines data without tumor tissue sequencing data.

We thank the reviewer for raising this important point. We agree that tumor tissue sequencing provides essential information about native tumor composition, microenvironmental interactions,

spatial organization, and clinical context. However, the primary goal of our study is to establish a large-scale single-cell multimodal resource of cancer cell lines for systematically characterizing cancer-cell-intrinsic regulatory programs under controlled conditions. Such a resource provides baseline information for evaluating the suitability of specific cancer cell-line models, assessing their representation of disease-relevant phenotypes, and nominating experimentally tractable systems for future mechanistic studies.

Our analyses indicate that cultured cancer cell lines retain biologically meaningful phenotypic and regulatory features related to their tumor origins. For example, gene program decomposition recovered melanoma phenotypic switching, a well-established axis of melanoma cell-state plasticity (**Fig. 4a-b**). In parallel, melanoma cell lines exhibited subtype-specific gene programs reflecting inflammatory signaling differences between acral and cutaneous melanoma (**Fig. 4a, c**). Thus, while the recapitulation of melanoma phenotypic switching serves as a positive control for the utility of our resource, the identification of systematic regulatory differences between acral and cutaneous melanoma highlights its potential for discovering new biology, especially given the rarity of acral melanoma and the limited availability of patient-derived resources.

Importantly, these observations were further evaluated using patient tumor cohorts and relevant clinical information. The phenotypic and signaling differences between acral and cutaneous melanoma were supported in the TMCH cohort[36] (**Fig. 4d**), which contains bulk RNA-seq profiles from 57 acral and cutaneous melanoma patients. Using TCGA-SKCM, we further observed associations between melanoma subtype-associated tumor programs and immune/stromal features (**Fig. 5a-c**), and corroborated the immunosuppressive transcriptomic phenotypes of TME cells associated with acral melanoma using patient-derived single-cell RNA-seq data[36] (**Fig. 5d-e**). In addition to the original clinical relevance analysis (**Fig. 5h-j**), this association was further supported in immunotherapy-treated melanoma cohorts (**Fig. R4-4**). In the Riaz2017 cohort[44], despite the marginal significance, possibly reflecting the limited sample size (**Fig. R4-4a**), our GRN signature ranked above other published immune signatures in relative power of predicting OS (**Fig. R4-4b**), reinforcing the importance of cancer-cell-intrinsic states. Consistently, in Campbell2023[43], an integrated resource containing more than 100 anti-PD-1-treated patients with prior anti-CTLA-4 therapy, our GRN signature significantly stratified patients for OS, with higher GRN scores associated with improved outcomes (**Fig. R4-4c**); this association remained significant after including age and sex as covariates (**Fig. R4-4d**).

Together, our analyses support that findings derived from our cell-line resource are reproducible in independent patient cohort datasets and associated with clinically relevant outcomes. We have clarified the intended scope of our study and the complementary value of tumor tissue sequencing in the revised manuscript (lines 43–57).

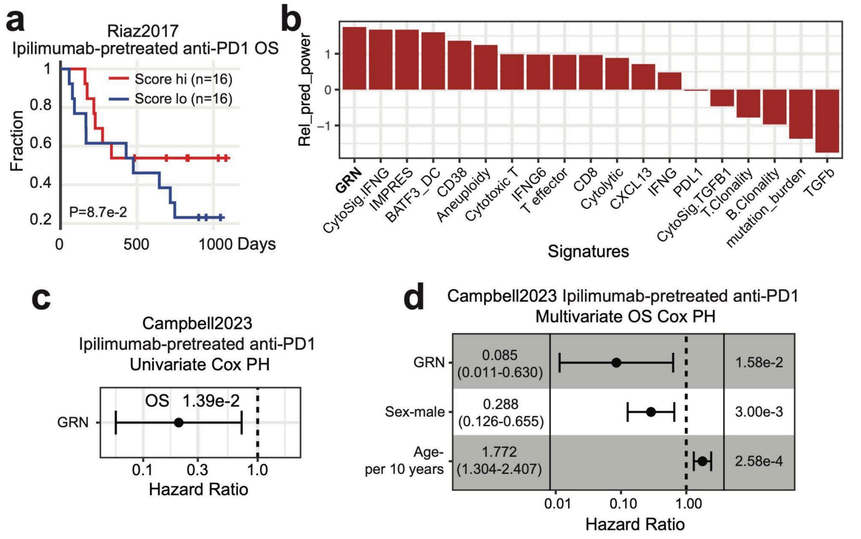

Fig. R4-4. Extended analyses using the GRN signature to stratify anti-PD-1-treated melanoma cohorts.

a, Kaplan-Meier curve of OS stratified by the GRN signature in ipilimumab-pretreated anti-PD-1-treated melanoma patients from the Riaz2017 cohort.

b, Relative predictive-power ranking of the GRN signature and published immunotherapy-associated signatures for OS in the Riaz2017 ipilimumab-pretreated anti-PD-1 cohort.

c, Univariate Cox proportional-hazards analysis showing the association between the GRN signature and OS in ipilimumab-pretreated anti-PD-1-treated patients from the Campbell2023 cohort.

d, Multivariable Cox proportional-hazards analysis of OS in ipilimumab-pretreated anti-PD-1-treated patients from the Campbell2023 cohort, including the GRN signature, sex, and age as covariates.

References:

1. Bravo González-Blas, C. *et al.* SCENIC+: single-cell multiomic inference of enhancers and gene regulatory networks. *Nat Methods* **20**, 1355–1367 (2023).
2. Kartha, V. K. *et al.* Functional inference of gene regulation using single-cell multi-omics. *Cell Genom* **2**, (2022).
3. Persad, S. *et al.* SEACells infers transcriptional and epigenomic cellular states from single-cell genomics data. *Nat Biotechnol* **41**, 1746–1757 (2023).
4. Sundaram, L. *et al.* Single-cell chromatin accessibility reveals malignant regulatory programs in primary human cancers. *Science* **385**, eadk9217 (2024).
5. Mo, C.-K. *et al.* Tumour evolution and microenvironment interactions in 2D and 3D space. *Nature* **634**, 1178–1186 (2024).
6. Wu, F. *et al.* Single-cell profiling of tumor heterogeneity and the microenvironment in advanced non-small cell lung cancer. *Nat Commun* **12**, 2540 (2021).
7. Kumar, V. *et al.* Single-Cell Atlas of Lineage States, Tumor Microenvironment, and Subtype-Specific Expression Programs in Gastric Cancer. *Cancer Discov* **12**, 670–691 (2022).
8. Sarkozy, C. *et al.* Integrated single cell analysis reveals co-evolution of malignant B cells and tumor micro-environment in transformed follicular lymphoma. *Cancer Cell* **42**, 1630 (2024).
9. Puram, S. V. *et al.* Single-Cell Transcriptomic Analysis of Primary and Metastatic Tumor Ecosystems in Head and Neck Cancer. *Cell* **171**, 1611–1624.e24 (2017).
10. Jacob, L. S. *et al.* Metastatic Competence Can Emerge with Selection of Preexisting Oncogenic Alleles without a Need of New Mutations. *Cancer Res* **75**, 3713–3719 (2015).
11. Dey, S. S., Kester, L., Spanjaard, B., Bienko, M. & van Oudenaarden, A. Integrated genome and transcriptome sequencing of the same cell. *Nat Biotechnol* **33**, 285–289 (2015).
12. Meng, X.-N. *et al.* Dynamic genomic changes in methotrexate-resistant human cancer cell

lines beyond DHFR amplification suggest potential new targets for preventing drug resistance. *Br J Cancer* **130**, 1819–1827 (2024).

13. Sziraki, A. *et al.* A global view of aging and Alzheimer's pathogenesis-associated cell population dynamics and molecular signatures in human and mouse brains. *Nat Genet* **55**, 2104–2116 (2023).
14. Sikkema, L. *et al.* An integrated cell atlas of the lung in health and disease. *Nat Med* **29**, 1563–1577 (2023).
15. Kumar, T. *et al.* A spatially resolved single-cell genomic atlas of the adult human breast. *Nature* **620**, 181–191 (2023).
16. Wang, K. *et al.* Coalescing single-cell genomes and transcriptomes to decode breast cancer progression. *Cell* (2025) doi:10.1016/j.cell.2025.08.012.
17. Cook, D. P. & Vanderhyden, B. C. Context specificity of the EMT transcriptional response. *Nat Commun* **11**, 2142 (2020).
18. Kotliar, D. *et al.* Reproducible single-cell annotation of programs underlying T cell subsets, activation states and functions. *Nat Methods* **22**, 1964–1980 (2025).
19. Caronni, N., La Terza, F., Frosio, L. & Ostuni, R. IL-1β macrophages and the control of pathogenic inflammation in cancer. *Trends Immunol* **46**, 403–415 (2025).
20. Yang, D., Liu, J., Qian, H. & Zhuang, Q. Cancer-associated fibroblasts: from basic science to anticancer therapy. *Exp Mol Med* **55**, 1322–1332 (2023).
21. Jiang, J. *et al.* D-SPIN constructs regulatory network models from scRNA-seq that reveal organizing principles of perturbation response. *Cell* (2026) doi:10.1016/j.cell.2026.04.028.
22. Xu, Z. *et al.* Mapping convergent regulators of melanoma drug resistance by PerturbFate. *Nature* (2026) doi:10.1038/s41586-026-10367-0.
23. Tsoi, J. *et al.* Multi-stage Differentiation Defines Melanoma Subtypes with Differential Vulnerability to Drug-Induced Iron-Dependent Oxidative Stress. *Cancer Cell* **33**, 890–904.e5 (2018).

24. Wouters, J. *et al.* Robust gene expression programs underlie recurrent cell states and phenotype switching in melanoma. *Nat Cell Biol* **22**, 986–998 (2020).

25. Zhu, Q. *et al.* Single cell multi-omics reveal intra-cell-line heterogeneity across human cancer cell lines. *Nat Commun* **14**, 8170 (2023).

26. Kuchur, O. A. *et al.* Targeting transcription in neuroblastoma: focus on the core regulatory circuit. *Expert Opin Ther Targets* **29**, 579–595 (2025).

27. Lupien, M. *et al.* FoxA1 translates epigenetic signatures into enhancer-driven lineage-specific transcription. *Cell* **132**, 958–970 (2008).

28. Centeno, P. P., Pavet, V. & Marais, R. The journey from melanocytes to melanoma. *Nat Rev Cancer* **23**, 372–390 (2023).

29. Chung, V. Y. *et al.* The role of GRHL2 and epigenetic remodeling in epithelial-mesenchymal plasticity in ovarian cancer cells. *Commun Biol* **2**, 272 (2019).

30. Johansson, J. *et al.* MiR-155-mediated loss of C/EBPβ shifts the TGF-β response from growth inhibition to epithelial-mesenchymal transition, invasion and metastasis in breast cancer. *Oncogene* **32**, 5614–5624 (2013).

31. Du, J. *et al.* FOXC1, a target of polycomb, inhibits metastasis of breast cancer cells. *Breast Cancer Res Treat* **131**, 65–73 (2012).

32. Lin, Y.-C. *et al.* Genome dynamics of the human embryonic kidney 293 lineage in response to cell biology manipulations. *Nat Commun* **5**, 4767 (2014).

33. GitHub - broadinstitute/infercnv: Inferring CNV from Single-Cell RNA-Seq. *GitHub* https://github.com/broadinstitute/infercnv.

34. Wei, C. *et al.* Delineating the early dissemination mechanisms of acral melanoma by integrating single-cell and spatial transcriptomic analyses. *Nat Commun* **14**, 8119 (2023).

35. Aran, D., Sirota, M. & Butte, A. J. Systematic pan-cancer analysis of tumour purity. *Nat Commun* **6**, 8971 (2015).

36. Zhang, C. *et al.* A single-cell analysis reveals tumor heterogeneity and immune

environment of acral melanoma. *Nat Commun* **13**, 7250 (2022).

37. Thakker, S. *et al.* Acral lentiginous melanoma. Part I. Epidemiology, etiology, clinical presentation, and diagnosis. *J Am Acad Dermatol* **94**, 421–432 (2026).
38. Wang, M. *et al.* The genetic evolution of acral melanoma. *Nat Commun* **15**, 6146 (2024).
39. Liu, D. *et al.* Integrative molecular and clinical modeling of clinical outcomes to PD1 blockade in patients with metastatic melanoma. *Nat Med* **25**, 1916–1927 (2019).
40. Van Allen, E. M. *et al.* Genomic correlates of response to CTLA-4 blockade in metastatic melanoma. *Science* **350**, 207–211 (2015).
41. Gong, L. *et al.* Cancer immunology data engine reveals secreted AOAH as a potential immunotherapy. *Cell* **188**, 5062–5080.e32 (2025).
42. Auslander, N. *et al.* Robust prediction of response to immune checkpoint blockade therapy in metastatic melanoma. *Nat Med* **24**, 1545–1549 (2018).
43. Campbell, K. M. *et al.* Prior anti-CTLA-4 therapy impacts molecular characteristics associated with anti-PD-1 response in advanced melanoma. *Cancer Cell* **41**, 791–806.e4 (2023).
44. Riaz, N. *et al.* Tumor and Microenvironment Evolution during Immunotherapy with Nivolumab. *Cell* **171**, 934–949.e16 (2017).
45. Gide, T. N. *et al.* Distinct Immune Cell Populations Define Response to Anti-PD-1 Monotherapy and Anti-PD-1/Anti-CTLA-4 Combined Therapy. *Cancer Cell* **35**, 238–255.e6 (2019).
46. Jang, S. R. *et al.* Association Between Sex and Immune Checkpoint Inhibitor Outcomes for Patients With Melanoma. *JAMA Netw Open* **4**, e2136823 (2021).
47. Feldker, N. *et al.* Genome-wide cooperation of EMT transcription factor ZEB1 with YAP and AP-1 in breast cancer. *EMBO J* **39**, e103209 (2020).
48. Southard, K. M. *et al.* Comprehensive transcription factor perturbations recapitulate fibroblast transcriptional states. *Nat Genet* **57**, 2323–2334 (2025).

49. Liu, H. *et al.* Integrative molecular and spatial analysis reveals evolutionary dynamics and tumor-immune interplay of in situ and invasive acral melanoma. *Cancer Cell* **42**, 1067–1085.e11 (2024).

50. Huang, A. *et al.* X-atlas/Orion: Genome-wide Perturb-seq datasets via a scalable Fix-Cryopreserve platform for training dose-dependent biological foundation models. *bioRxiv* (2025) doi:10.1101/2025.06.11.659105.

Reviewer #1 (Remarks to the Author):

I appreciate the authors' careful and comprehensive responses to the first-round comments. The revised manuscript is substantially improved, and the authors have done an excellent job addressing the prior concerns through additional clarification, analyses, and revisions.

Overall, I find the study to be rigorous, clearly presented, and of high value as a resource for the community. I have no additional major concerns and support publication of the manuscript.

Reviewer #1 (Remarks on code availability):

The authors have made the code available, and it appears to be a useful and reasonably organized resource accompanying the manuscript.

We sincerely thank the reviewer for acknowledging our study.

Reviewer #2 (Remarks to the Author):

I co-reviewed this manuscript with one of the reviewers who provided the listed reports. This is part of the Nature Communications initiative to facilitate training in peer review and to provide appropriate recognition for Early Career Researchers who co-review manuscripts.

Reviewer #2 (Remarks on code availability):

No, the code does not provide a README file. In addition, it is not clear based on the code availability how one would reproduce all analyses performed. Minimal R and python functions are currently present on the repository.

We sincerely thank the reviewer for the constructive comment. To improve the clarity and usability of the code repository, we have updated it to include a README file that describes the required inputs, expected outputs, and the purpose of each script. We have also added more comprehensive analysis scripts to better support reproduction of the analyses performed in the manuscript. We hope that the updated code repository will be more useful to readers.

Reviewer #3 (Remarks to the Author):

The authors have addressed all my comments thoroughly and effectively.
I have no further comments.

Reviewer #3 (Remarks on code availability):

The scripts are available. To improve transparency and clarity, consider providing additional information in the GitHub repository, such as indicating which scripts correspond to which figures and specifying where the source data (e.g., input files for each script) are located.

We sincerely thank the reviewer for the acknowledgement and the constructive comment. To improve the clarity and usability of the code repository, we have updated it to include a README file that describes the required inputs, expected outputs, and the purpose of each script. We have also added more comprehensive analysis scripts. We hope that the updated code repository will be more useful to readers.

Reviewer #4 (Remarks to the Author):

The revised version answer my concerns.

Reviewer #4 (Remarks on code availability):

The revised version answered my concerns.

We sincerely thank the reviewer for acknowledging our study.
